# Xuebijing Injection Maintains GRP78 Expression to Prevent *Candida albicans*–Induced Epithelial Death in the Kidney

**DOI:** 10.3389/fphar.2019.01416

**Published:** 2020-01-06

**Authors:** Ting Shang, Qilin Yu, Tongtong Ren, Xin-Tong Wang, Hongyan Zhu, Jia-Ming Gao, Guixiang Pan, Xiumei Gao, Yan Zhu, Yuxin Feng, Ming-Chun Li

**Affiliations:** ^1^Tianjin State Key Laboratory of Modern Chinese Medicine, Tianjin University of Traditional Chinese Medicine, Tianjin, China; ^2^Research and Development Center of TCM, Tianjin International Joint Academy of Biomedicine, TEDA, Tianjin, China; ^3^Key Laboratory of Molecular Microbiology and Technology for Ministry of Education, College of Life Sciences, Nankai University, Tianjin, China

**Keywords:** fungal infection, *C. albicans*, Xuebijing injection, endoplasmic reticulum stress, GRP78, Chinese medicine

## Abstract

Sepsis and septic shock threaten the survival of millions of patients in the intensive care unit. Secondary fungal infections significantly increased the risk of mortality in sepsis patients. Chinese medicine Xuebijing injection (XBJ) has been routinely used as an add-on treatment to sepsis and septic shock in China. Our network pharmacology analysis predicted that XBJ also influences fungal infection, consisting with results of pioneer clinical studies. We conducted *in vivo* and *in vitro* experiments to verify this prediction. To our surprise, XBJ rescued mice from lethal *Candida* sepsis in a disseminated *Candida albicans* infection model and abolished the colonization of *C. albicans* in kidneys. Although XBJ did not inhibit the growth and the virulence of *C. albicans in vitro*, it enhanced the viability of 293T cells upon *C. albicans* insults. Further RNA-seq analysis revealed that XBJ activated the endoplasmic reticulum (ER) stress pathway upon *C. albicans* infection. Western blot confirmed that XBJ maintained the expression of GRP78 in the presence of *C. albicans*. Interestingly, key active ingredients in XBJ (C0127) mirrored the effects of XBJ. C0127 not only rescued mice from lethal *Candida* sepsis and prevented the colonization of *C. albicans* in kidneys, but also sustained the survival of kidney epithelial cells partially by maintaining the expression of GRP78. These results suggested that XBJ may prevent fungal infection in sepsis patients. Pre-activation of ER stress pathway is a novel strategy to control *C. albicans* infection. Network pharmacology may accelerate drug development in the field of infectious diseases.

## Highlights

1. Xuebijing (XBJ) prevented *Candida* sepsis in a murine model.
2. XBJ maintained GRP78 expression to prevent *C. albicans*–induced kidney epithelial death.
3. The key compounds in XBJ suppressed *Candida* sepsis and kidney failure partially by regulating GRP78.

## Introduction

Fungal infection causes an annual mortality of 1.5 million people worldwide (Wirnsberger et al., 2016). The cost of treating invasive fungal infection is over 2 billion dollars in the US (Pfaller and Diekema, 2010). As the leading pathogen in patients suffering invasive fungal infections, *Candida albicans* fostered 50% of candida sepsis cases (Pfaller and Diekema, 2010; Brown et al., 2012). Associated with a mortality rate exceeding 40%, past decades witnessed a dramatic rise in the incidence of invasive candidiasis (Kullberg and Arendrup, 2015).

Limited choices of antifungal drugs are available to treat fungal infections with only two non-toxic antifungal classes for candidiasis (Diekema et al., 2012). Azoles are applied in clinical practice to treat *C. albicans*–related infections (Sakagami et al., 2018). Nevertheless, invasive *C. albicans* infection still claims mortality of 45% to 75% (Brown et al., 2012). Emerging drug-resistant fungal infections are also calling for novel management strategies to restrain fungal sepsis (Healey et al., 2016).

*C. albicans*–induced kidney failure is a major cause of mortality in *C. albicans* sepsis (Spellberg et al., 2005). Enhancing the function of the innate immune system rescued lethal *C. albicans* infections in murine models (Xiao et al., 2016; Dominguez-Andres et al., 2017). Other potential mechanisms remain elusive. Administrating mirR-124 and mirR-204 mimics prevented *C. albicans*–induced acute kidney injury (Li et al., 2014b; Li et al., 2018).

Secretory and membrane proteins are synthesized and modified in the endoplasmic reticulum (ER) of mammalian cells (Yu et al., 2015; Zhu and Lee, 2015). Activating ER stress signaling renders survival advantage for tissues and cells upon *C. albicans* infection. Glucose-regulated proteins (GRPs) are constitutively expressed in cells to maintain cellular homeostasis, belonging to the heat shock protein family as stress-inducible chaperones. Infections activate GRPs to translocate in the cells to assume functions such as regulating signaling transduction, proliferation and immunity (Zhu and Lee, 2015; Lewy et al., 2017). Conserved from yeast to human, GRP78 (BiP) is one of such proteins that regulate homeostasis of organs from endoderm, mesoderm, and ectoderm. Interestingly, GRP78 cross-talks with PI3K/AKT pathway, which sustains cell survival (Shani et al., 2008; Gray et al., 2013; Liu et al., 2013).

Xuebijing (XBJ) injection was prepared with extracts from five different Chinese herbs [*Carthamus tinctorius* flowers (Honghua), *Paeonia lactiflora* roots (Chishao), *Ligusticum chuanxiong* rhizomes (Chuanxiong), *Angelica sinensis* roots (Danggui), and Salvia miltiorrhiza roots (Danshen)] (Cheng et al., 2016; Li et al., 2016; Li et al., 2019; Zhang et al., 2018). Approved by the Food and Drug Administration of China in 2004, XBJ has been frequently used as an add-on therapy for multiple organ dysfunction syndromes, sepsis, and septic shock in China for over a decade (Chen et al., 2018a; Gao et al., 2015; Shi et al., 2017). It rendered a series of benefits for sepsis patients, including reducing 28-day mortality and incidence of complications, shortening dwelling time in the intensive care unit (Gao et al., 2015; Shi et al., 2017; Song et al., 2019). Pre-clinical studies indicated XBJ might be a treatment option for sepsis and septic shock individually (Jiang et al., 2013; Chen et al., 2018). Four classes of compounds from five different herbs in XBJ may be important for its antiseptic effect (Li et al., 2016). Intensive research is going on to identify major active compounds in XBJ that can effectively treat sepsis (Cheng et al., 2016; Li et al., 2016). Combining Xuebijing with anti-fungal agents or antibiotics had positive impacts on the quality of life of patients suffering invasive fungal infections in several clinical studies and may improve the survival of patients (Gao, 2010; Wang, 2010; Cao, 2017). However, it was not clear whether XBJ can influence fungal infection individually.

Our network pharmacology analysis predicted that XBJ not only affects therapeutic targets of sepsis but also influences fungal infection, suggesting XBJ may prevent fungal infection in sepsis patients. Here we reported that XBJ prevented systemic *C. albicans* infections. Notably, XBJ pretreatment protected 70% of mice from mortality after systemic *C. albicans* infection. It prevented the colonization of *C. albicans* in kidneys and enhanced the viability of kidney epithelial cells by sustaining ER stress signaling.

## Methods

### Chemicals and Reagents

Xuebijing injection (catalog number: z20039833, batch number: 1606121) was manufactured by Tianjin Chase Sun Pharmaceutical Co., Ltd (Tianjin, China). This Chinese medicine is approved by the China Food and Drug Administration (CFDA) for treating sepsis and septic shock with the CFDA ratification number of GuoYaoZhunZi-Z20039833 for market approval as a drug product. It is routinely used as an add-on to conventional therapy for treating sepsis and septic shock in China (Jiang et al., 2013; Chen et al., 2018; Li et al., 2019). This injection contains extracts of five herbs, including *Carthamus tinctorius* flowers (Honghua in Chinese), *Paeonia lactiflora* roots (Chishao in Chinese), *Ligusticum chuanxiong* rhizomes (Chuanxiong in Chinese), *Angelica sinensis* roots (Danggui in Chinese), and Salvia miltiorrhiza roots (Danshen in Chinese).

Methods of extraction, preparation, and quality control of XBJ were the same as previously reported (Huang et al., 2011; Chen et al., 2016; Li et al., 2016; Zhang et al., 2018). Briefly, ingredients from *Carthamus tinctorius flowers* (“Honghua” in Chinese) were first extracted with ethanol then with water. Ingredients from the other four herbs were extracted with water. Finally, XBJ was standardized to contain 1.0 to 1.7 mg/ml of paeoniflorin and 0.2 to 0.5 mg/ml of hydroxysafflor yellow A as described (Huang et al., 2011; Chen et al., 2016; Li et al., 2016).

GRP78 inhibitor HA15 and other chemicals used in the experiments were ordered from Sigma-Aldrich (Shanghai, China) unless indicated. Paeoniflorin (Cas #: 23180-57-6), hydroxysafflor yellow A (Cas#: 78281-02-4), ferulic acid (Cas #:537-98-4), and protocatechualdehyde (Cas#:139-85-5) were purchased from Shanghai Yuanye Biotechnology Co., Ltd. (Shanghai, China). C0127 was prepared with paeoniflorin, hydroxysafflor yellow A, ferulic acid, and protocatechualdehyde according to reported concentrations in XBJ and manufacturer’s quality control information (Liu et al., 2015; Zuo et al., 2017; Zuo et al., 2018). The structures of the four compounds in C0127 were presented in **Supplementary Figure 1**. Western blotting was performed using the GRP78 monoclonal antibody and tubulin monoclonal antibodies (Abcam, USA).

### *C. albicans* Strain and Growth Conditions

*C. albicans* strain SC5314 (ATCC, USA), which was routinely cultivated in YPD (1% yeast extract, 2% peptone, 2% glucose), was used for all experiments in this study. The *C. albicans* cells were cultured overnight at 30°C and washed twice with PBS for further use.

### Animal Experiments and Ethics Statement

This study was carried out in accordance with the recommendations of the Guide for the Care and Use of Laboratory Animals (NIH Publication No. 85-23, revised 1996, USA) and the recommendations in the Guidance for the Care and Use of Laboratory Animals issued by the Ministry of Science and Technology of China. All experiments were approved by the Institutional Animal Care and Use Committee of Nankai University and the Laboratory Animal Ethics Committee of the Tianjin University of Traditional Chinese Medicine (Tianjin, China) and were performed in accordance with its guidelines (Permit Number: TCM-LAE-20170017). Five to six week-old ICR female mice were used for *in vivo* experiments (Dong et al., 2017). The mice were provided with free access to food and sterile water and were caged under controlled temperature (23°C ± 2°C) and humidity (60% ± 5%) with an artificial 12 h light/dark cycle. The mice were randomly divided into six groups (n = 15 in each group) as follows: Control group injected with normal saline; CA group was infected with 5 × 10^6^ colony-forming units of *C. albicans via* tail-vein injection. XBJ group treated with XBJ (6 ml/kg; Chase Sun Pharmaceutical, Ltd., Tianjin, China) by subcutaneous injection; CA+XBJ group infected with *C. albicans* and co-treated with XBJ (6 ml/kg); C0127 group infected with C. *albicans* after 3 injections of C0127 (6 ml/kg). XBJ and C0127 were administered once/day from Day -3 to Day -1 before the C. albicans infection. The *C. albicans* strain SC5314 was cultivated in YPD (1% yeast extract, 2% peptone, and 2% dextrose) broth with overnight shaking at 30˚C. The systemic fungal infection and virulence assays were performed as described (Dong et al., 2017; Liang et al., 2018).

### Database Construction and Network Analysis

Fungal infection-related targets were mainly integrated from literature mining, GeneCards (Stelzer et al., 2011) and Ingenuity Pathway Analysis (IPA, http://www.ingenuity.com) database (Kramer et al., 2014). Repetitive genes were automatically identified and removed by IPA software. In addition, ingredients derived from XBJ were collected from literature mining (Huang et al., 2011; Jiang et al., 2013; Guo et al., 2014; Han et al., 2017; Zuo et al., 2017; Zuo et al., 2018) and several TCM databases, such as TCMID (Xue et al., 2013) and TCMSP (Ru et al., 2014). Compounds that had more than 10 targets in mammalians were selected for further analysis. The chemical name of each compound was transferred into PubChem CID or CAS number which could be recognized by the IPA software. Furthermore, corresponding targets of XBJ ingredients that were not recorded by the IPA database were supplemented by literature from PubChem and databases of TCMID and TCMSP. In total, three datasets including XBJ ingredients, fungal infection associated targets, and corresponding targets of XBJ’s major ingredients were constructed and then uploaded into the IPA system to visualize the discovery. The relationships between fungal infection-related targets and XBJ ingredients were discovered by “Build-Path Explorer” module. “Build-Connection” module was implemented to interpret the relationship between targets. “Overlay-Canonical Pathway” module was used to generate the resulting canonical pathways. “Build-Diseases & Functions” module was exploited to build the targets-related diseases and functions. We utilized the “Core analysis” module to analyze the correlation of the established network to acquire top diseases, top functions, top pathways, and top upstream regulators. Certain top upstream regulators were defined by the “Upstream Regulator” module. The “Path designer” module was performed to clarify the network. Upstream regulators analyses were performed to elucidate the causal inference of upstream biological causes and probable downstream effects on cellular and organismal biology (Kramer et al., 2014). “Path designer” module was used to demonstrate the network. The algorithm of the network analysis was based on Fisher’s exact test with the enrichment score of P-values in this study.

### RNA Isolation, RNA-seq, and Quantitative Real-Time Polymerase Chain Reaction

Total RNA was extracted by the phenol-chloroform method as previously described (Dong et al., 2017). The overall quality of RNA was determined by A260/A280 and analyzed by agarose gel electrophoresis. Roche GS-FLX 454 pyrosequencing was carried out using Illumina HiSeq^™^ 2000 (Oebiotech Company, China). Reverse transcriptions were conducted with an Oligo (dT)-primed RT reagent Kit (Promega, USA). Quantitative real-time polymerase chain reaction (PCR) was performed in triplicate and repeated in three independent experiments with the Mastercycler ep realplex system. Independent reaction mixtures were carried out with the same DNA template for both the genes of interest and the GAPDH reference gene using the RealMasterMix (SYBR Green) Kit (Trans-Gen Biotech, China) according to the instructions. The relative fold changes in gene expression were determined by the 2-delta delta Ct method. Data were presented as means ± SD of three independent experiments.

### Hematoxylin and Eosin Staining

Hematoxylin and eosin (H&E) staining was conducted as described (Dong et al., 2017). Briefly, kidney tissues were collected 4 days after *C. albicans* infection and were fixed with 4% formalin at room temperature for 24 h, dehydrated with alcohol, and paraffin−embedded. The tissues were then cut into 5-µm-thick sections, which were stained with H&E at room temperature for 1 to 2 min and visualized under a microscope (BX53, Olympus, Japan).

### Western Blotting

Protein was extracted from 293T cells using the RIPA solution. The protein concentration of the lysates was measured with the Bradford assay. Western blotting was conducted as described (Fu et al., 2007).

### Statistical Analysis

Data were presented as the mean ± standard deviation for each group. All statistical analyses were performed using PRISM version 5.0 (GraphPad Software, Inc., La Jolla, CA, USA). Inter-group differences were analyzed using one-way analysis of variance, followed by Tukey’s post-hoc test for multiple comparisons. The log-rank test was used to compare group survival trends. *P* < 0.05 was considered to indicate a statistically significant difference.

## Results

### Network Pharmacology Analysis Predicted That XBJ May Regulate Fungal Infection

**Figure 1:**
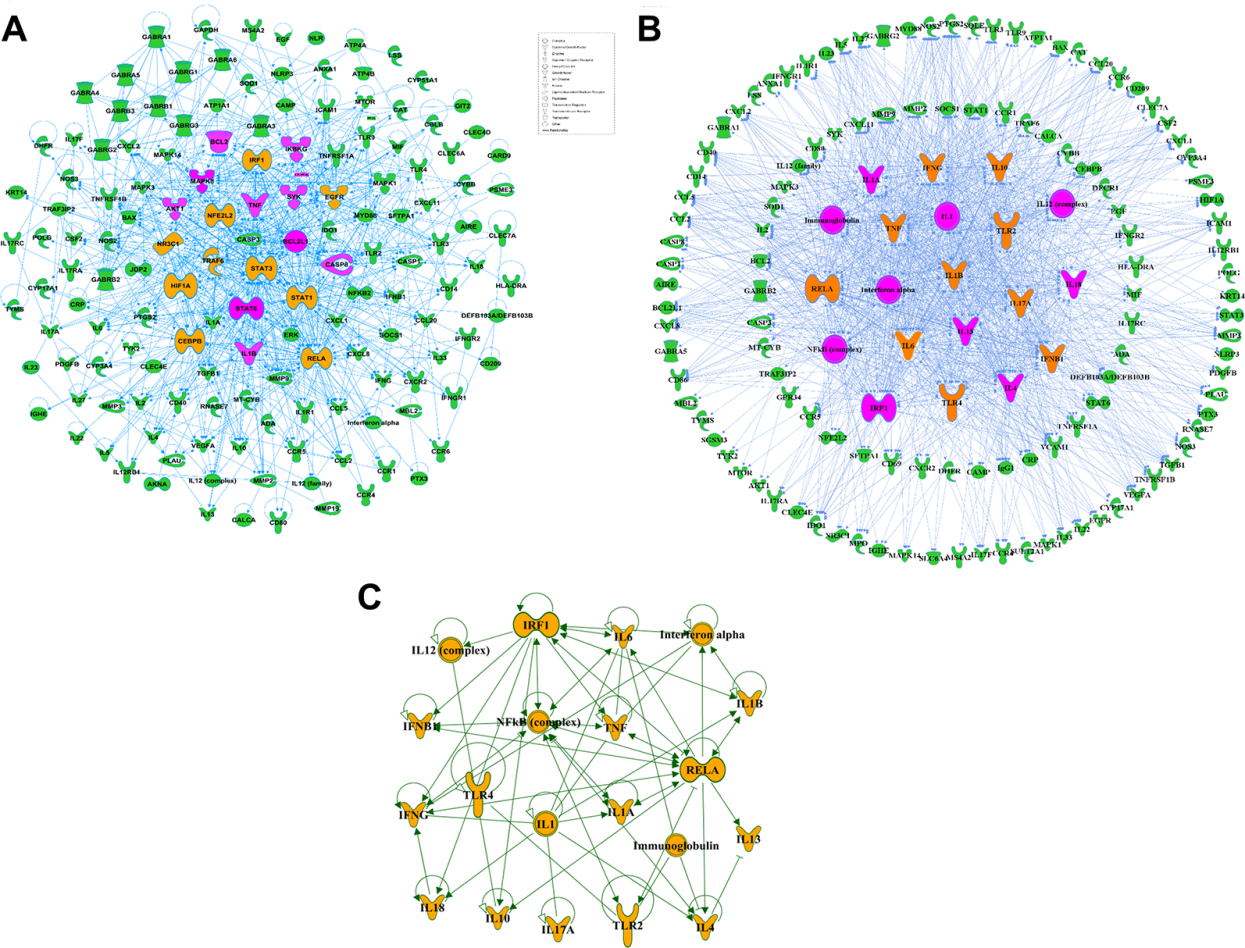
Target proteins related to fungal infection were identified in network pharmacology analysis. **(A)** Protein-protein interactions of the potential and proved therapeutic targets in fungal infection. The top ten targets predicted by IPA were marked in saffron yellow and the top 20 molecules were labeled with purple-red. **(B)** Top 20 upstream regulators of the potential and proved therapeutic targets in fungal infection and their relationships with the related targets. The top ten upstream regulators predicted by IPA were marked in saffron yellow and the top 20 targets were marked in purple-red. **(C)** The network among top 20 upstream regulators of the potential and proved therapeutic targets in fungal infection predicted by IPA.

**Figure 2:**
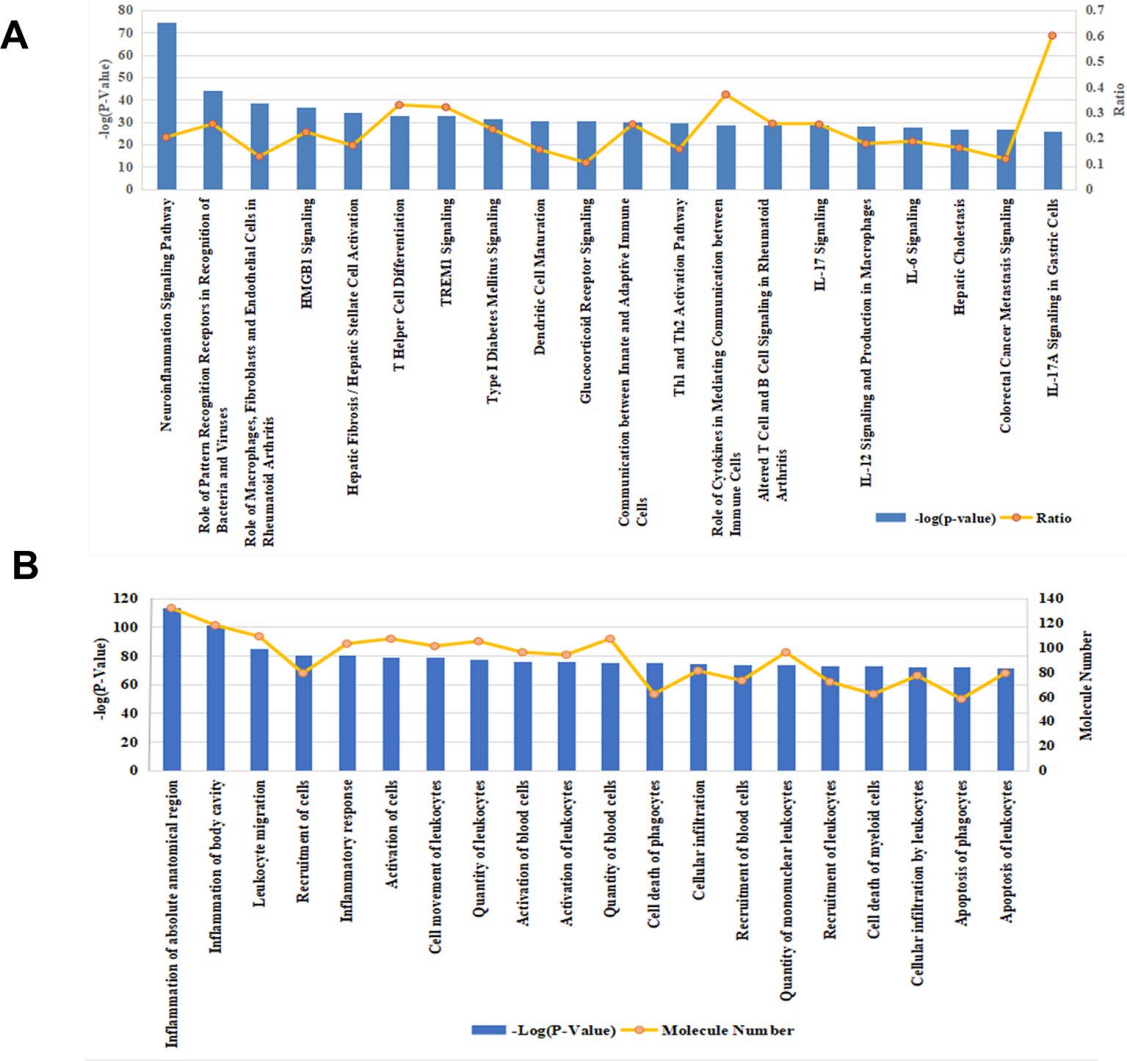
Functional analysis and pathway analysis of 170 potential therapeutic targets in fungal infection. **(A)** The top 20 related pathways among molecules relevant to fungal infection. **(B)** Functional analysis of the 170 fungal infection target proteins. The top 20 pathways/functions were ranked from left to right by –log (*P* value) and the ratio (yellow line) referred to the proportion of 170 molecules in the respective pathway.

Effective in controlling systemic bacterial infection, XBJ has potentials in treating a series of diseases related to infections and tissue injuries (Wang et al., 2015; Xu et al., 2015; Chen et al., 2018; Hu et al., 2018; Tian et al., 2018). We conducted network pharmacology analysis to explore novel applications of XBJ. A library of 170 proteins/molecules related to fungal infection was built and top upstream regulators of these proteins were identified by IPA software (**Figure 1A** and **Supplementary Table 1**). Toll-like receptors (TLR2, TLR4), pro-inflammatory cytokines [tumor necrosis factor, interleukin 6 (IL-6), IL-1, and interferons], nuclear factor κB signaling, and HMGB1 were among top upstream regulators of the targets in fungal infection (**Figures 1B, C**). Interestingly, many of these molecules are known as Xuebijing targets (Jiang et al., 2013; Chen et al., 2018). These results indicated that XBJ may impact fungal infection. Pathway analysis revealed that HMGB1 signaling is among the top 4 pathways in fungal infection (**Figure 2A**). This echoes the reports that XBJ may target HMGB1 to attenuate organ injuries (Wang et al., 2007; Wang et al., 2015; Chen et al., 2016). In addition, multiple potential targets are related to inflammation and cell survival in the functional analysis (**Figure 2B**).

**Figure 3:**
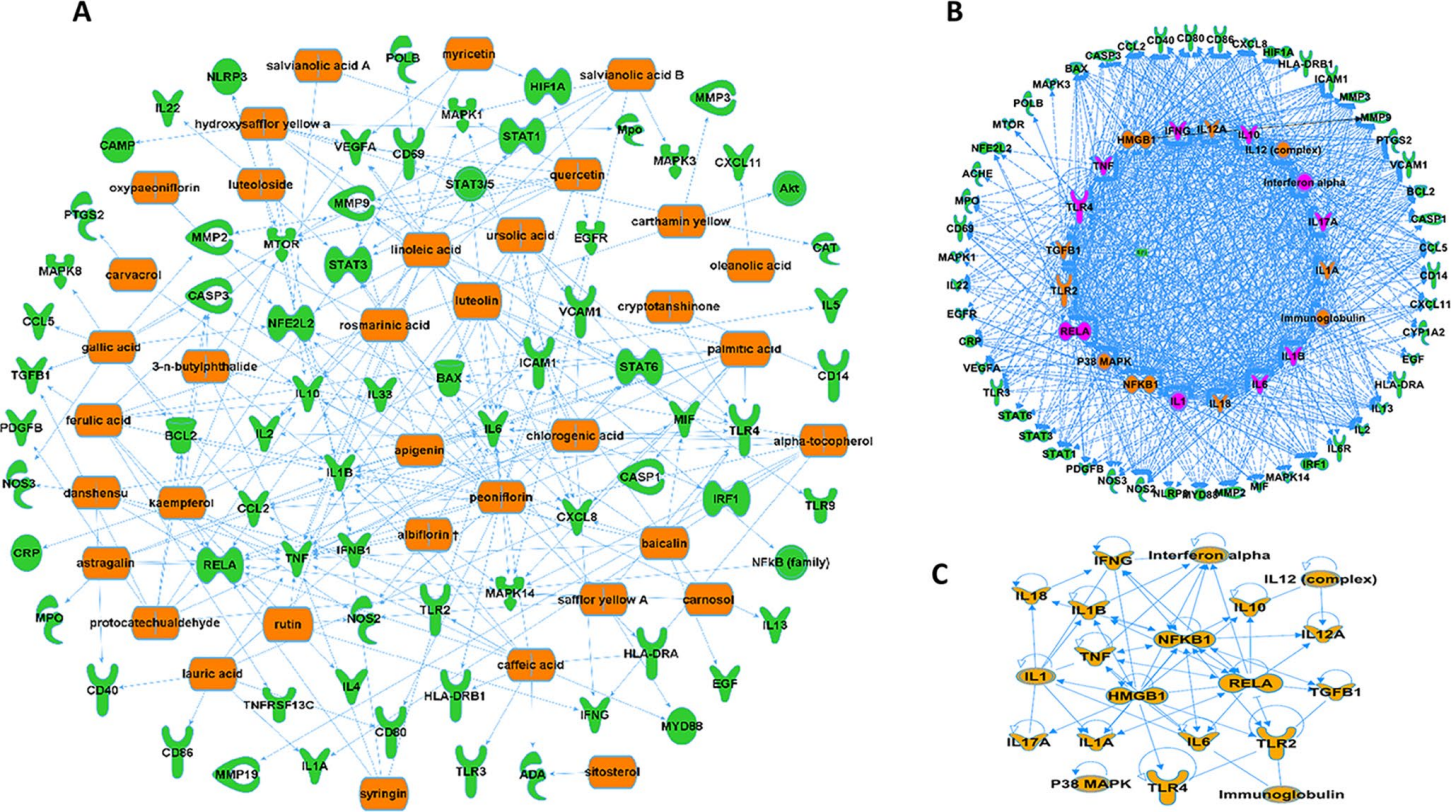
XBJ ingredients regulate potential therapeutic targets of fungal infection. **(A)** XBJ ingredient-target network predicted by IPA. Thirty-six compounds from XBJ were predicted to cooperatively modulate 72 fungal infection-related targets. **(B)** Top 20 upstream regulators of potential XBJ targets in fungal infection (top 20 regulators were marked with saffron yellow and top 10 key molecules were labeled with purple-red). **(C)** The IPA-predicted network of top 20 upstream regulators of potential XBJ targets shown in **Figure 3B**.

**Figure 4:**
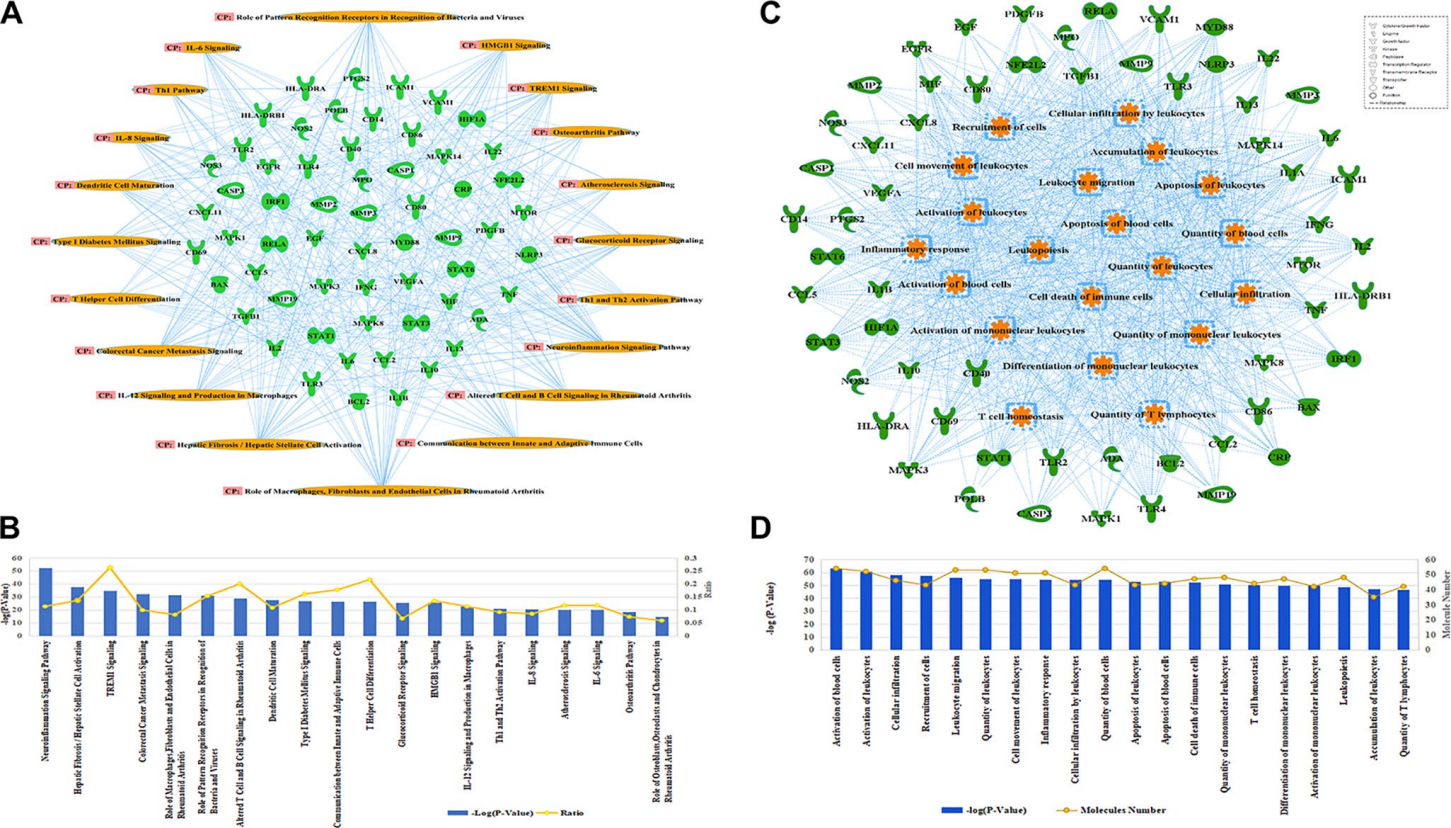
Functional and pathway analysis of potential XBJ targets in fungal infection. **(A**, **B)** Pathway analysis of XBJ targets in fungal infection. **(A)** The relationships of potential XBJ targets and top 20 related pathways. **(B)** The ranking of the top 20 signaling pathways of potential XBJ targets was presented. The top 20 signaling pathways of potential XBJ targets in fungal infection were ranked from left to right by –log (*P* value). **(C**, **D)** Functional analysis of XBJ targets in fungal infection. **(C)** The target-function network was presented with the top 20 functions marked in saffron yellow. **(D)** The ranking of the top 20 functions of potential XBJ targets was presented. The top 20 related functions were ranked from left to right by –log (*P* value).

A further analysis predicted that 36 compounds derived from five herbs in XBJ regulate over 70 molecules related to fungal infection (**Figure 3A**). Indeed, many key upstream regulators among these molecules were XBJ targets (**Figures 3B, C**). IPA pathway analysis foretold that these 36 compounds targeted HMGB1, IL-6, and signaling related to organ injuries which were among the top 20 pathways (**Figures 4A, B**). Our functional analysis predicted that XBJ targets play roles in cell activation, survival, and apoptosis (**Figures 4C, D**). Based on these results, we hypothesized that XBJ prevents fungal infection and decided to test this hypothesis in a well-established *C. albicans* sepsis model (Dominguez-Andres et al., 2017; Zhao et al., 2017).

### Xuebijing Prevented *C. albicans*–Induced Septic Shock in a Murine Model

**Figure 5:**
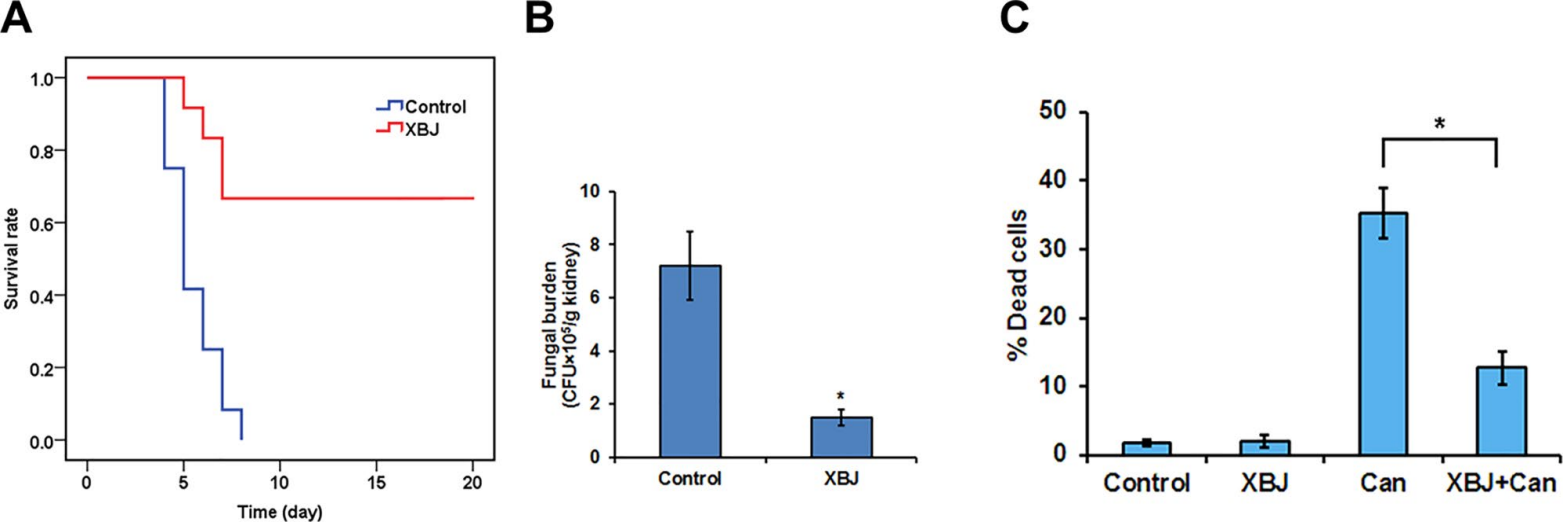
XBJ pre-treatment inhibits the colonization of *C. albicans* in the kidney. **(A)** The survival curves of XBJ pre-treated and control mice after systemic *C. albicans* infection. XBJ (6 ml/kg) or 0.9% NaCl was administered by abdominal injections once/day for 3 days before systemic *C. albicans* infection to the treatment group or the control group. **(B)** The colony-forming assay to determine the fungal loads in the kidneys of control and XBJ treated mice. Kidney tissues from control and XBJ treated kidneys were harvested and subjected to *C. albicans* culture 4 days after the infection. Colonies were counted after 48 h of culture. **(C)** PI staining was used to determine the effect of XBJ on the survival of 293T cells 8 h after the *C. albicans* infection. *P < 0.05.

All infected mice died 8 days after systemic *C. albicans* infection. 6 ml/kg XBJ pre-treatment for 3 days rescued ∼70% of *C. albicans* infected mice from acute death (**Figure 5A**), indicating that it may prevent systemic *C. albicans* infection to arrest the consequent acute death. To address the question of whether XBJ directly affects the growth of *C. albicans*, we treated *C. albicans* in culture with different dilutions of XBJ (from 1:100 to 1:1000). However, XBJ did not affect the growth of *C. albicans* and hyphal development (data not shown), indicating the mechanism is related to the defense system in hosts. The functional analysis by IPA predicted that XBJ targets signaling pathways involved in organ damage and cell death (**Figures 4C, D**). This is consistent with clinical observations (Shi et al., 2017; Song et al., 2018). Thus, we hypothesized that XBJ improves the survival of *C. albicans*–infected mice partially through protecting kidneys.

### XBJ Prevented *C. albicans* Colonization in the Kidney

To determine whether *C. albicans* colonizes kidney after XBJ intervention, we conducted a histopathological analysis of mouse kidneys, and determined the fungal load in kidneys 4 days after *C. albicans* infection. While hyphae, neutrophil penetration, and tissue damages were detected in kidneys of the control group, few hyphae were detected in the XBJ treated group. Neutrophil penetration and tissue damage were also reduced in the XBJ treated group (**Figure 7C**), suggesting XBJ inhibited *C. albicans* colonization in kidneys. Consistently, the kidney fungal burden decreased threefold in XBJ treated mice in the colony-forming assay (**Figure 5B**). No *C. albicans* was detected in blood and kidney tissues 3 weeks after the infection in XBJ treated mice (data not shown). Next, we determined the effect of XBJ on kidney epithelial cell survival in different conditions. XBJ treatment at 1:100 dilution did not affect the survival of 293T cells. However, it significantly reduced cell death from ∼35% to 15% upon *C. albicans* infection (**Figure 5C**).

### XBJ Up-Regulated the ER Stress Signaling Pathway During *C. albicans* Infection

**Figure 6:**
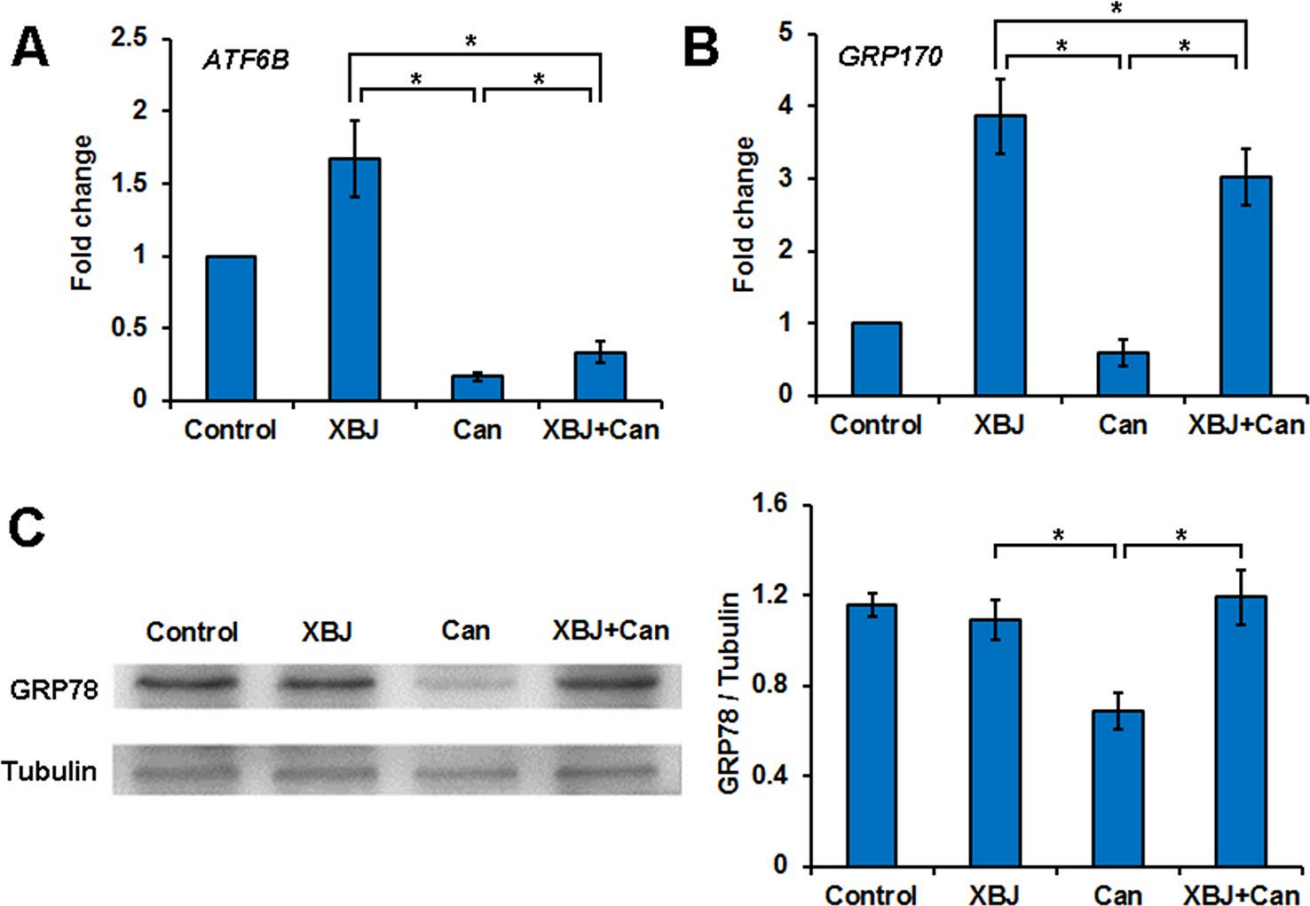
XBJ up-regulates the ER stress signaling pathway during *C. albicans* infection. Six groups of 293T cells in different conditions were subjected to RNA-seq analysis, including cells treated with XBJ only, infected with *C. albicans*, 293T cells infected with *C. albicans* in the presence of XBJ (1:100 dilution). **(A**, **B)** RNA-seq results of ATF6B and GRP170, two genes in the ER stress pathway. **(C)** Western blot to determine the expression of GRP78 expression in different conditions in 293T cells. Data were representative of at least three independent experiments. *P < 0.05.

To determine how XBJ protects kidney cells, 293T cells under different treatments were subjected to RNA-seq analysis, including cells treated with saline, cells treated with XBJ only, cells infected with *C. albicans* only, and cells infected with *C. albicans* in the presence of XBJ (1:100 dilution). While *C. albicans* infection inhibited the ER stress signaling, XBJ treatment restored the expression of ER stress signaling. XBJ up-regulated the ATF6B and GRP170 on the mRNA level (**Figures 6A, B**). This was further confirmed by Western blot of GRP78. XBJ restored GRP78 expression to a similar level as control groups (non-infected 293T cells and non-infected 293T cells treated with XBJ) (**Figure 6C**). Therefore, XBJ may maintain the expression of the key factors in the ER stress signaling pathway to improve kidney epithelial cell survival.

### Key Ingredients in XBJ Rescued Mice From Septic Shock and Prevented the Colonization of *C. albicans* in the Kidney

**Figure 7:**
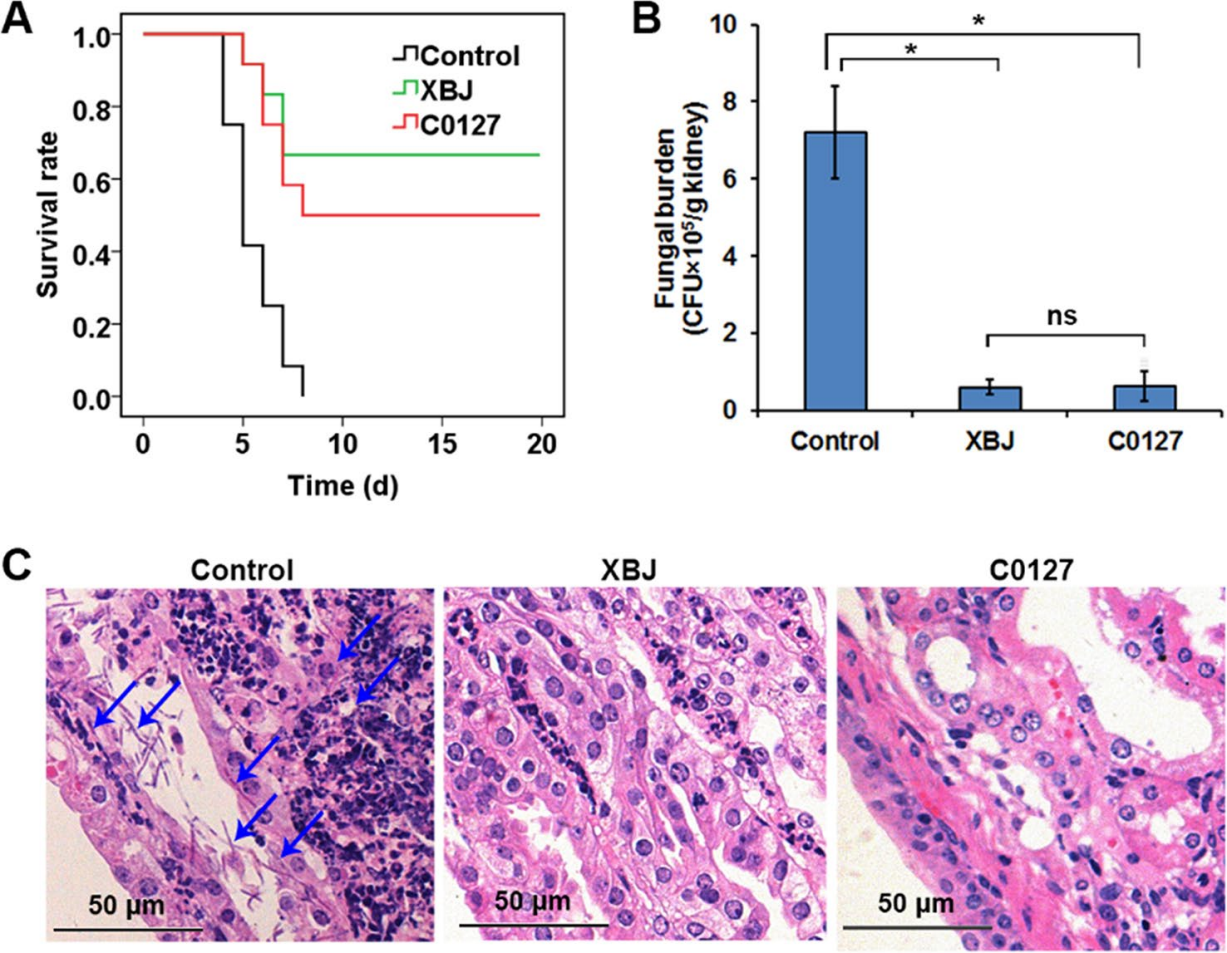
Key compounds (C0127) in XBJ rescued mice from lethal infection of *C. albicans*. **(A)** Survival curves of different groups of mice in *Candida* infected (Control) and treatment groups. Control vs. XBJ and Control vs. C0127: *P* < 0.05. Control group: N = 11; XBJ and C0127 group: N = 12. **(B)** Colony-forming assay to evaluate fungal load in kidneys 4 days after *Candida* infection. *P < 0.05. **(C)** Hematoxylin and eosin staining to determine the histology in kidneys. Arrows indicated fungal hyphae in an infected kidney.

Network pharmacology analysis suggested that four major compounds in XBJ may regulate most XBJ targets related to fungal infection (**Supplementary Figure 2**). To understand the mechanism of XBJ on improving the survival in *C. Albicans* sepsis, C0127, a formula comprised of the four major active compounds from XBJ, was used to treat mice before the systemic *C. albicans* infection. Similar to XBJ, pre-treatment with C0127 not only significantly improved the survival of *C. albicans* infected mice but also decreased fungal loads in kidneys (**Figures 7A, B**). Similarly, hyphae and neutrophils infiltration can hardly be detected 4 days after *C. albicans* infection following the C0127 pre-treatment (**Figure 7C**).

### Key Ingredients in XBJ Up-Regulated GRP78 to Improve 293T Cell Survival Upon *C. albicans* Infection

**Figure 8:**
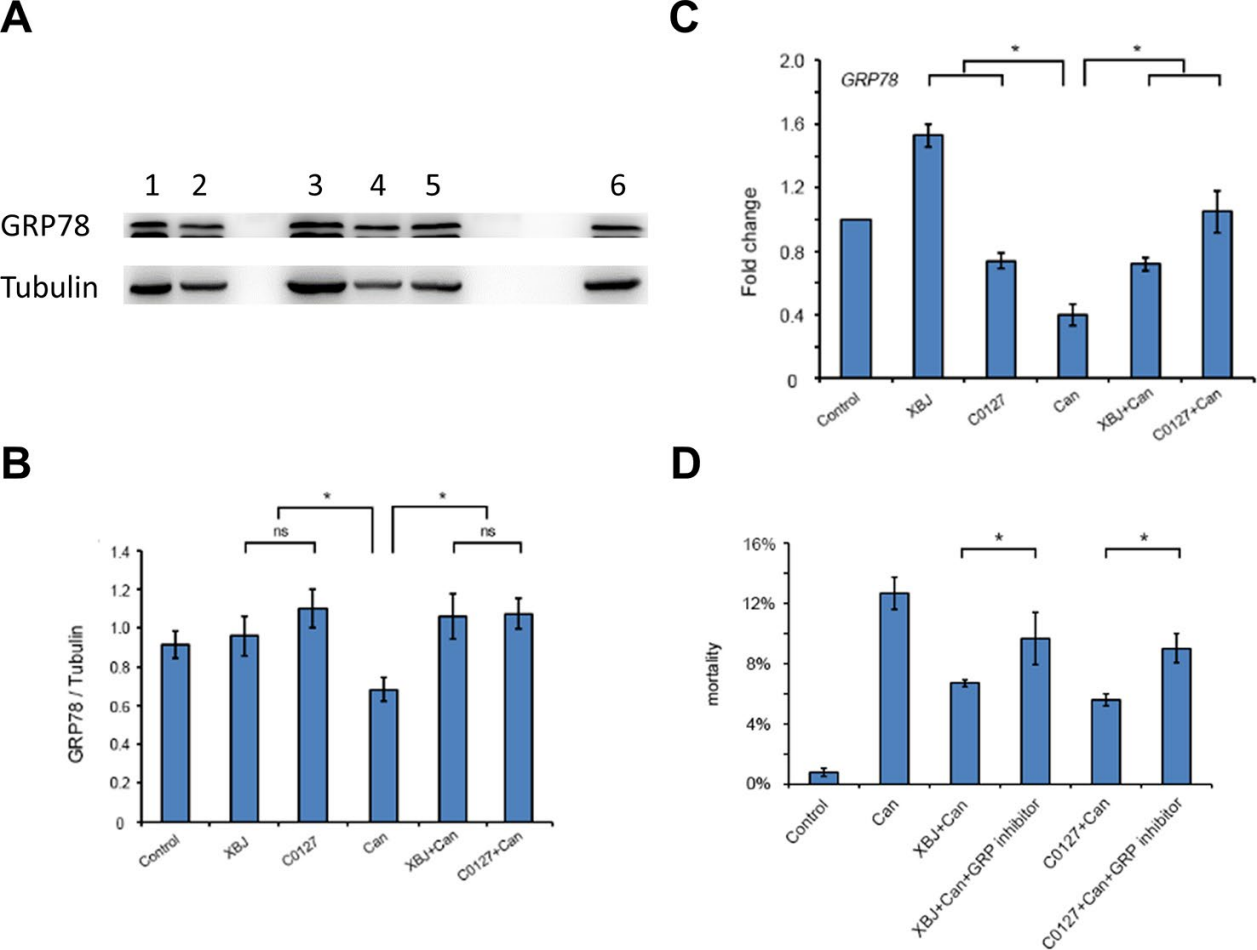
XBJ and C0127 sustained GRP78 expression to improve the survival of 293T cells. **(A)** C0127 maintained GRP78 expression on protein level in 293T cells upon *C. albicans* infection. Western blot experiments were conducted to determine the expression of GRP78. Tubulin was used as an internal control. Lane 1. Control 293T cells; lane 2. C. *Albicans* infected 293T cells; lane 3. XBJ treated 293T cells; lane 4. C. *albicans* infected 293T cells treated with C0127; lane 5. C. *albicans* infected 293T cells treated with XBJ; lane 6. C0127 treated 293T cells. **(B)** Quantification of the GRP78 protein in 293T cells upon different treatments. Data were representative of at least three independent experiments with similar results. **(C)** Real-time PCR to determine the mRNA expression of GRP78 in 293T cells upon different treatments. **(D)** The influence of HA15, a GRP78 inhibitor, on the survival of 293T cells in low serum culture under different conditions by PI staining. Data were representative of at least three independent experiments with similar results. *P < 0.05.

Like XBJ, C0127 maintained GRP78 expression on the protein level in 293T cells upon the *C. albicans* insult (**Figures 8A, B**). In our preliminary study, HA15, a GRP78 specific inhibitor (Cerezo et al., 2016; Ruggiero et al., 2018), induced apoptosis in 293T cells in low-serum culture (data not shown). To determine whether GRP78 is required for the protective effect of XBJ and C0127, 293T cells were treated with HA15 upon *C. albicans* infection. It induced cell death in the presence of XBJ and C0127, indicating GRP78 is an important downstream effector of XBJ and C0127 for the survival of kidney cells (**Figure 8D**).

## Discussion

### Summary of the Results and Significance

Our network pharmacology analysis predicted that XBJ may impact fungal infection. XBJ rescued lethal *Candida* sepsis and improved kidney epithelial cell survival by maintaining the expression of GRP78. In addition, C0127, a combination of four key compounds in XBJ also prevented *C. albicans*–induced acute death in mice and the colonization of *C. albicans* in the kidney. It improved kidney epithelial cells’ survival by up-regulating GRP78. These results revealed a novel mechanism of XBJ in preventing organ failure and cell death. GRP78 was identified as a potential novel target of XBJ in preventing *C. albicans* infection. Our results suggested that network pharmacology is beneficial to identify novel applications of XBJ and other Chinese medicine formulas.

### Mechanism of Kidney Failure in *Candida* Sepsis

During the bloodstream infection, *C. albicans* attaches to the host and then penetrates the host defense system to attack the target organs such as the kidney. It is characterized by the presence of hyphae and the damages to host cells (Brunke and Hube, 2013). The innate immune system plays an important role in controlling the progression of *Candida* sepsis. The damaged cells secrete pro-inflammation cytokines to recruit innate immune cells such as neutrophils to clear *C. albicans* (Wirnsberger et al., 2016; Dominguez-Andres et al., 2017).

Enhancing/activating the function of the innate immune system counteracts the progression of *Candida* sepsis by improving *C. albicans* clearance. Switching off a series of immune inhibitory regulators such as the E3 ubiquitin ligase CBLB, Sts, and Jnk1 significantly improved resistance to *C. albicans* in mice (Wirnsberger et al., 2016; Xiao et al., 2016; Zhao et al., 2017). The E3 ubiquitin ligase CBLB in the innate immune system negatively impacts the phagocytosis of neutrophils and macrophages (Wirnsberger et al., 2016; Xiao et al., 2016). The type I interferon-induced IL-15 production in the Ly6C^high^ monocytes is also required for *C. albicans* clearance by innate immune cells (Dominguez-Andres et al., 2017). In contrast, compromising the function of the innate immune system accelerates *C. albicans*–induced kidney failure (Dominguez-Andres et al., 2017).

Other mechanisms that enhance host defense against *C. albicans* infection were not extensively studied. Both global and local inflammation contribute to *C. albicans*–induced kidney failure. Li et al. showed the systematic expression of microRNAs prevented *C. albicans* infection (Li et al., 2014b; Li et al., 2018). They found *C. albicans* induced acute kidney injury by attenuating miR-124 expression and up-regulating MIP-1 in the kidney (Li et al., 2018), indicating local inflammation in the kidney contributed to kidney failure. Administrating miR-124 mimic inhibited MIP-1 expression in the kidney and restored kidney functions (Li et al., 2018). It is still not clear how miR-124 and miR-204 regulate innate immune system. However, global inflammation in the absence of *C. albicans* may not be sufficient to induce kidney failure. *C. albicans* kills kidney epithelial cells directly, while XBJ partially rescued 293T cell survival upon *C. albicans* exposures (**Figure 6**). This is consistent with our previous result that XBJ improved RAW264.7 cells survival upon the insult of LPS (Lyu et al., 2018).

### XBJ and Organ Failure

XBJ has been approved to treat multiple organ dysfunction syndrome and sepsis in China for over a decade (Gao et al., 2015; Shi et al., 2017). A Meta-analysis by Song et al. demonstrated that combining XBJ with conventional intervention is superior to conventional intervention alone in treating MODS (Song et al., 2018). The benefits of XBJ to different organs (including lung, kidney, heart, and liver) were reported in a series of clinical studies (Fang and Wang, 2013; Wang et al., 2015; Zhang et al., 2016; Gao et al., 2018; Song et al., 2018) (Song et al., 2019). In a small-scale prospective, single-center, randomized double-blinded trial, Gao et al. found XBJ significantly lowered IL-1β, IL-8, and C-reactive protein in blood and up-regulated IL-10 in blood and decreased adverse events in patients with lung injury (Gao et al., 2018). Zhang et al. found XBJ improved myocardial function in patients with septic myocardial injury, indicated by improving cardiac troponin I, N-terminal proB-type natriuretic peptide, and procalcitonin in blood samples of patients (Zhang et al., 2016).

The major causes of organ failure in sepsis are disseminated intravascular coagulation (DIC), dysfunction of circulation, and cytokine storm. Results from basic research confirmed clinical observations and provided clues to the working mechanism of XBJ in preventing and treating different types of organ injuries systemically. It improves microcirculation partially by inhibiting blood-clotting to prevents/treats organ injuries (Wang et al., 2015; Xu et al., 2015; Jin et al., 2018). This is reflected in reversing abnormalities of metabolic biomarkers in sepsis (Shi et al., 2017).

XBJ is also likely to prevent/reverse organ injuries by improving cell survival and regulating cell functions (Chen et al., 2013; Li et al., 2014a). Li et al. reported XBJ enhanced survival of hematopoietic stem cells and mono-nuclear cells upon radiation insults *in vitro* and *in vivo*. XBJ also regulated the secretory function of Kupffer cells in heat stroke rats (Chen et al., 2013).

At the molecular level, XBJ regulates multiple signaling pathways to combat organ injuries. It down-regulated TLR4 signaling and stimulated the expression of Toll-interacting protein to improve organ functions (Liu et al., 2014b; Liu et al., 2014c; He et al., 2018). XBJ reduced ROS production in rats to attenuate pulmonary injury (Chen et al., 2017). P38 MAPK might be a XBJ target in a lung injury model (Liu et al., 2014a). HMGB1, a nuclear protein related to organ injury, is a biomarker of organ injury and a potential therapeutic target of organ injury (Wang et al., 1999; Musumeci et al., 2014). A series of publications indicated XBJ attenuates HMGB1 expression in sepsis organ injuries (Li et al., 2007; Wang et al., 2007; Wang et al., 2015; Chen et al., 2016). However, no gain-of-function and loss-of-function study has been conducted *in vivo* to determine the influences of XBJ on these pathways.

XBJ was used to treat and prevent different types of kidney injuries in the clinic and pre-clinical studies. In a clinical study, XBJ improved clinical symptoms of sepsis-induced acute kidney injury (Yuxi et al., 2017). In addition, XBJ attenuated herbicide paraquat-induced acute kidney injury in rats (Xu et al., 2017). It also prevented paraquat-induced apoptosis in human kidney cell line HK-2 (Tian et al., 2018). However, mechanisms of these effects remain to be illustrated. In this study, we revealed that XBJ may regulate GRP78 to improve the survival of kidney epithelial cells upon *C. albicans* insult and the combination of four compounds in XBJ can maintain GRP78 expression. This mechanism may also apply to other organs and cell types.

### The Advantages of Utilizing Different Methods to Understand the Working Mechanism of XBJ in Preventing Invasive *C. albicans* Infection

Network pharmacology predictions are based on our current knowledge. The experimental pharmacology has value to supplement network pharmacology to expand our knowledge. Our results showed that combining both methods provides advantages to advance medicine. RNA-seq, real-time PCR, and Western blot were used to identify novel signaling that impacts kidney failure during invasive *C. albicans* infection. Glucose-Regulated Protein 78 (GRP78, BiP) was identified by Western Blot in 293T cells. Results of real-time PCR confirmed the influence of XBJ on GRP78 at the transcription level. However, this result remains to be confirmed *in vivo*. ATF6B and GRP170, two ER stress-related proteins were influenced by XBJ in RNA-seq. But we did not find their changes on protein level (data not shown). Whether XBJ influences the expression of GRP94, another important ER stress protein that shares a similar function with GRP78 (Zhu and Lee, 2015), is under investigation. Overall, combining multiple methods is superior to a single method approach in understanding the working mechanism of XBJ in preventing invasive *C. albicans* infection.

### ER Stress Pathway in Cell Death and Organ Failure

GRP78 is a negative regulator of the unfolded protein response (UPR). Knocking down GRP78 triggered the UPR in un-stressed cells (Pyrko et al., 2007; Li et al., 2008). GRP78 represses apoptosis by inhibiting BIK and caspase-7 activation (Reddy et al., 2003; Fu et al., 2007). The function of GRP78 was not determined in a kidney-specific knockout animal model yet. However, its liver-specific knockdown induced liver injury, suggesting GRP78 plays a role in organ protection and may render protection for the kidney upon *C. albicans* insults (Ji et al., 2011; Chen et al., 2014; Zhu and Lee, 2015). Consistent with the literature, inhibiting the function of GRP78 increased the death of 293T cells (**Figure 8**). HA15, a specific GRP78 inhibitor, did not induce a dramatic increase of 293T cell death. This may due to the redundant function of GRP78 and GRP94 (Zhu and Lee, 2015). This hypothesis remains to be tested.

### Network Pharmacology in Developing Novel Regimens to Prevent Fungal Infection

Network pharmacology may shed light on developing novel Chinese medicine (Lyu et al., 2017; Suo et al., 2017). In this study, it also provided hints for a potential application of XBJ. Our aim was to take the advantages of network pharmacology to reveal a novel mechanism of XBJ in preventing invasive fungal infection. ER stress signaling was not predicted as top signaling related to invasive fungal infection by our network pharmacology analysis. In addition, our literature mining did not retrieve strong evidence indicating an important role of ER stress signaling in invasive fungal infection. It emerged from our RNA-seq analysis using RNA extracted from 293T cells in the presence of *C. albicans* and XBJ treatments. The alteration of GRP78 was more pronounced on the protein level rather than the transcription level in *C. albicans*–infected 293T cells treated by XBJ. However, Western Blot confirmed XBJ and C0127 did sustain the expression of GRP78 (**Figures 6** and **8**). Hence, our new finding indicated network pharmacology analysis and experiments complemented each other in illustrating the mechanism of compound Chinese medicine in treating human diseases.

### C0127 Prevents *C. albicans*–Induced Kidney Failure

Our network pharmacology analysis predicted that four compounds in XBJ regulated 2/3 of predicted XBJ targets in fungal infection (**Figure 3A** and **Supplementary Figure 2**). This prediction was confirmed in our *in vivo* study (**Figure 7**). C0127 not only prevented *Candida* sepsis but also prevented *C. albicans* colonization in the kidney. Paeoniflorin and hydroxysafflor yellow A, the top two high-concentration compounds in XBJ, may play major roles in preventing kidney failure upon systemic *C. albican*s infection. Paeoniflorin which claims the highest concentration in XBJ was isolated from Chishao (Liu et al., 2015; Han et al., 2017). Several groups reported that paeoniflorin attenuates ER stress in different tissues and organs (Chen et al., 2018a; Gu et al., 2016; Jiang et al., 2014; Zhu et al., 2018). Gu et al. indicated paeoniflorin exerted protection for MCAO rats by regulating ER stress. Zhu et al. reported that paeoniflorin attenuates ER stress in retinal pigment epithelial cells *via* triggering Ca(2+)/CaMKII-dependent activation of AMPK (Zhu et al., 2018). It is likely that paeoniflorin plays a major role in regulating the expression of GRP78 in kidney epithelial cells. However, this was not verified by our *in vitro* experiments. Paeoniflorin did not significantly enhance the expression of GRP78 individually (data not shown). Consistent with our results, Gu et al. found that a combination of *L. chuanxiong* and *Radix paeoniae*, two herbs in XBJ, attenuated ER stress–dependent apoptotic signaling pathway in MCAO rats (Gu et al., 2016). Hydroxysafflor yellow A (HSYA) plays a role in preventing tissue injuries (Han et al., 2017). Bai et al. reported the protective effect of HSYA on acute kidney injury in an ischemia/reperfusion (I/R) model. They found HSYA prevented I/R induced apoptosis in kidney epithelial cells *in vivo* and *in vitro*. HSYA may attenuate TLR4 signaling to prevent apoptosis in kidney epithelial cells (Bai et al., 2018). Increasing the stability of HSYA in XBJ may further enhance the protection to the kidney (Pu et al., 2017). In our experiment, XBJ or C0127 does not inhibit *C. albicans* growth *in vitro*. This is consistent with the observation of Canturk which showed 1mg/ml ferulic acid-induced necrosis in *C. albicans* and it synergistically enhanced the anti-fungal effect of caspofungin (Canturk, 2018). Thus, low conc. of ferulic acid is unlikely to influence the survival of *C. albicans* in systematic infection. The combination of four compounds does not have a synergistic inhibition on the growth of *C. albicans in vitro* either (data not shown).

### Future Directions

Our network pharmacology analysis predicted that XBJ regulates type I interferon and its upstream regulators such as interferon α, TLR2, and TLR4 in invasive fungal infection (**Figures 3B, C**). Thus, we hypothesized that XBJ may regulate type I interferon signaling in the innate immune system to enhance the clearance of *C. albicans* in invasive *C. albicans* infection. XBJ and C0127 may regulate innate immune system to enhance the survival of *Candida* infected mice while enhancing the survival of kidney epithelial cells. We also aim to determine how XBJ and C0127 regulate the innate immune cells and whether type I interferon signaling mediates their effects.

## Conclusions

In conclusion, XBJ may prevent systemic *C. albicans* infection in sepsis patients. Compounds with higher concentrations in XBJ played major roles in preventing *Candida* induced kidney failure. GRP78 is a novel target of XBJ and C0127 in kidney epithelial cells. Members of ER stress signaling might be novel therapeutic targets in organ protection and sepsis.

## Ethics Statement

This study was carried out in accordance with the recommendations of the Guide for the Care and Use of Laboratory Animals (NIH Publication No. 85-23, revised 1996, USA) and the recommendations in the Guidance for the Care and Use of Laboratory Animals issued by the Ministry of Science and Technology of China. All experiments were approved by the Institutional Animal Care and Use Committee of Nankai University and the Laboratory Animal Ethics Committee of the Tianjin University of Traditional Chinese Medicine (Tianjin, china) and were performed in accordance with its guidelines (Permit Number: TCM-LAE-20170017).

## Author Contributions

QY, YF, and M-CL designed the study and developed the methodologies. TS, QY, YF, TR, X-TW, HZ and J-MG conducted research. QY, YF, TS, TR, HZ, X-TW, J-MG, GP, XG, YZ and M-C.L. analyzed the data and contributed critical reagents. QY, YF, YZ, TS, and M-CL wrote and revised the manuscript.

## Funding

This project was supported by the National Science Foundation of China (Grant number: 81774018, 81973581, 81873037, 31670146, 81873961); Tianjin University of Traditional Chinese Medicine (Startup Grant for Y.F.); Nankai University (The Open Fund of Ministry of Education Key Laboratory of Molecular Microbiology and Technology); Tianjin Municipal Education Commission (Grant number: TD13-5046).

## Conflict of Interest

The authors declare that the research was conducted in the absence of any commercial or financial relationships that could be construed as a potential conflict of interest.
