# Supplementary figures and images for: Xuebijing Injection Maintains GRP78 Expression to Prevent Candida albicans–Induced Epithelial Death in the Kidney
Source: Front Pharmacol. 2020 Jan 6;10:1416. doi: 10.3389/fphar.2019.01416 (PMC6956827; doi:10.3389/fphar.2019.01416)

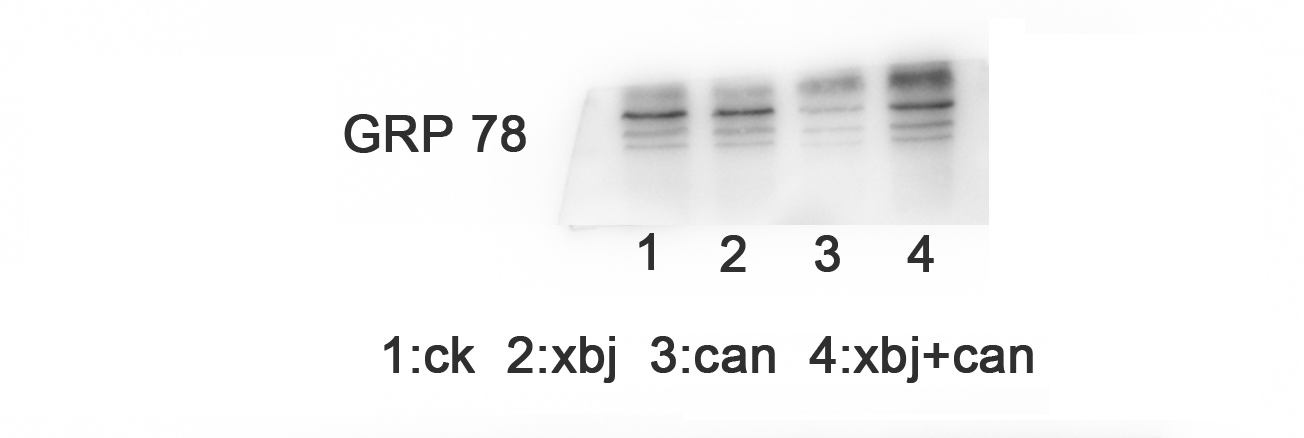

Supplement: Supplementary file 1 [file DataSheet_3.zip › Original-western/Figure6-GRP78.tif]

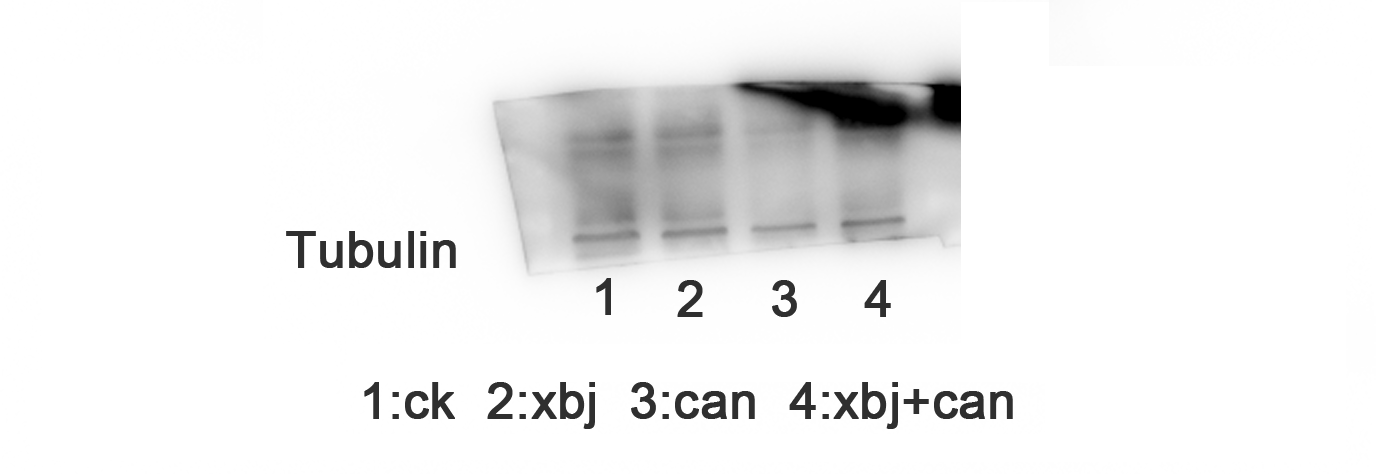

Supplement: Supplementary file 1 [file DataSheet_3.zip › Original-western/Figure6-Tubulin.tif]

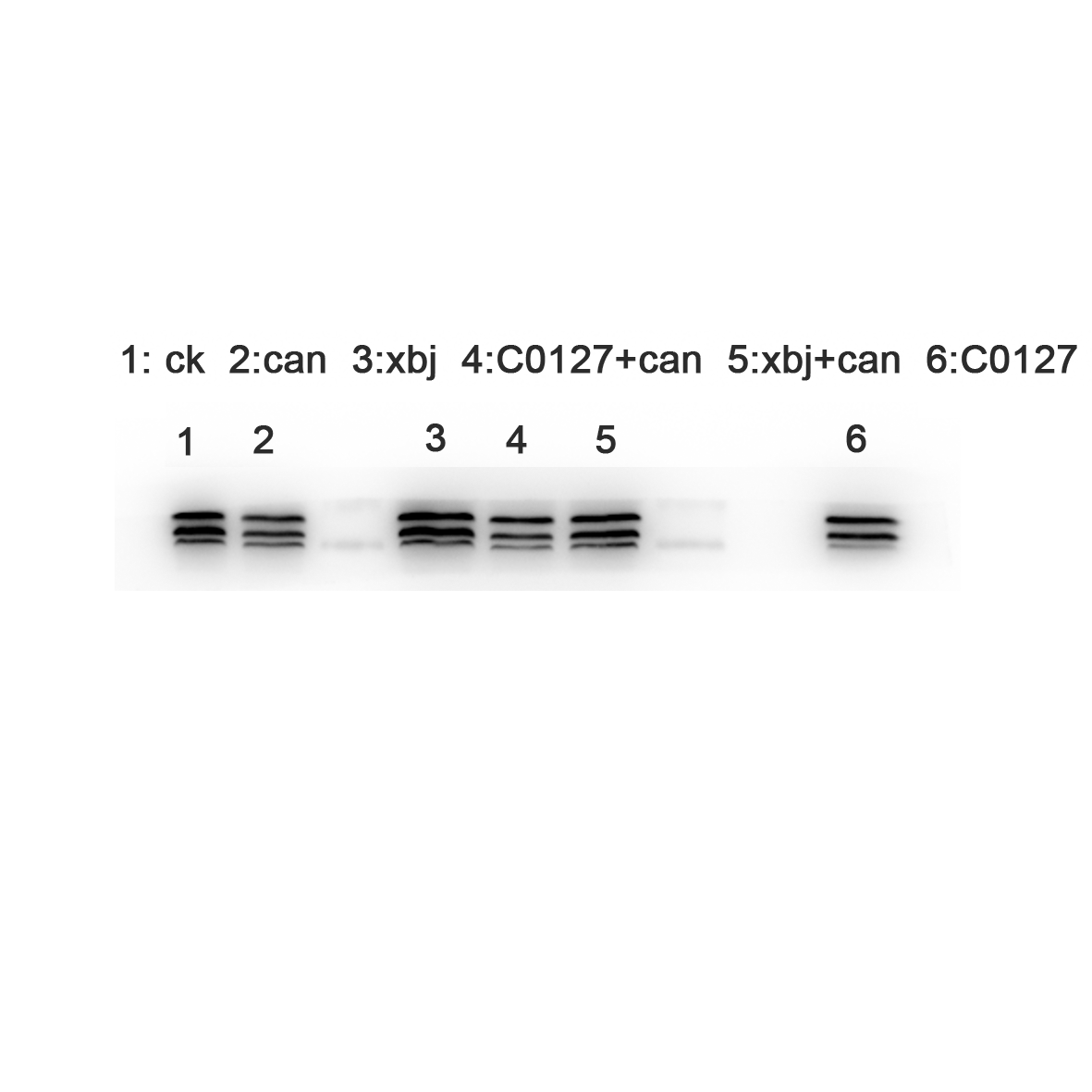

Supplement: Supplementary file 1 [file DataSheet_3.zip › Original-western/Figure8-GRP78.tif]

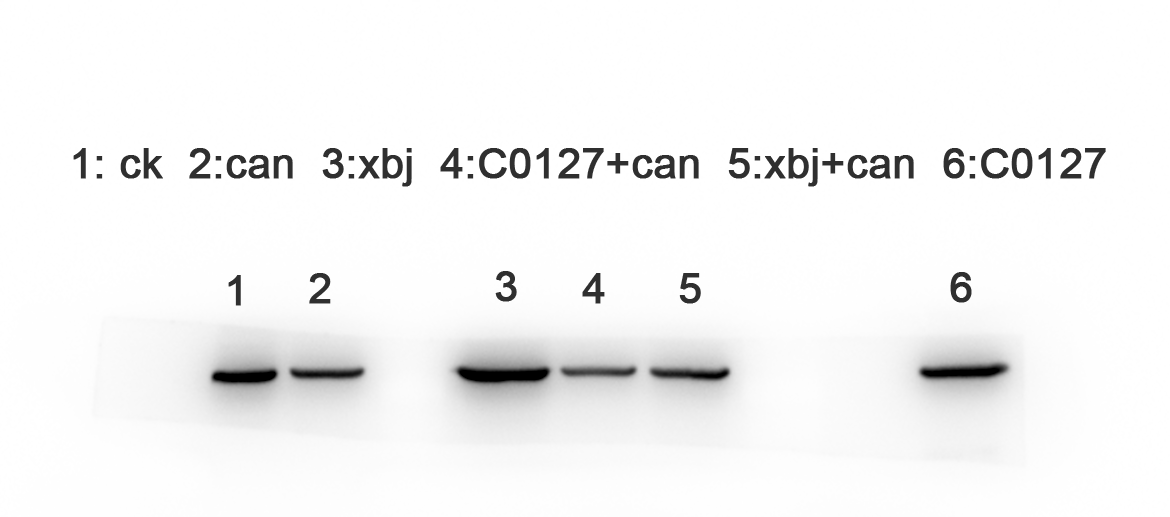

Supplement: Supplementary file 1 [file DataSheet_3.zip › Original-western/Figure8-Tubulin.tif]

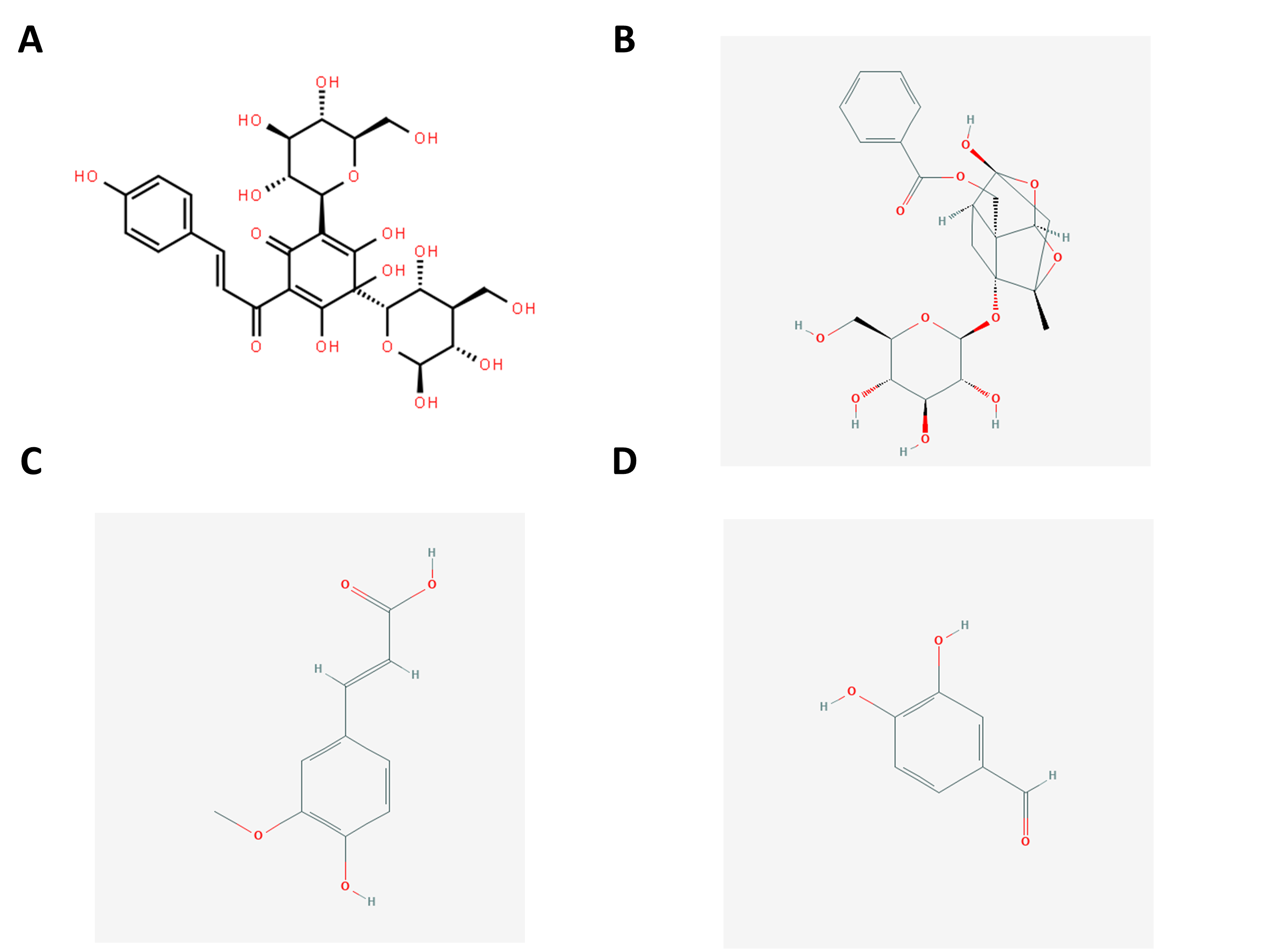

Supplement: Supplementary file 2 [file Image_1.tif]

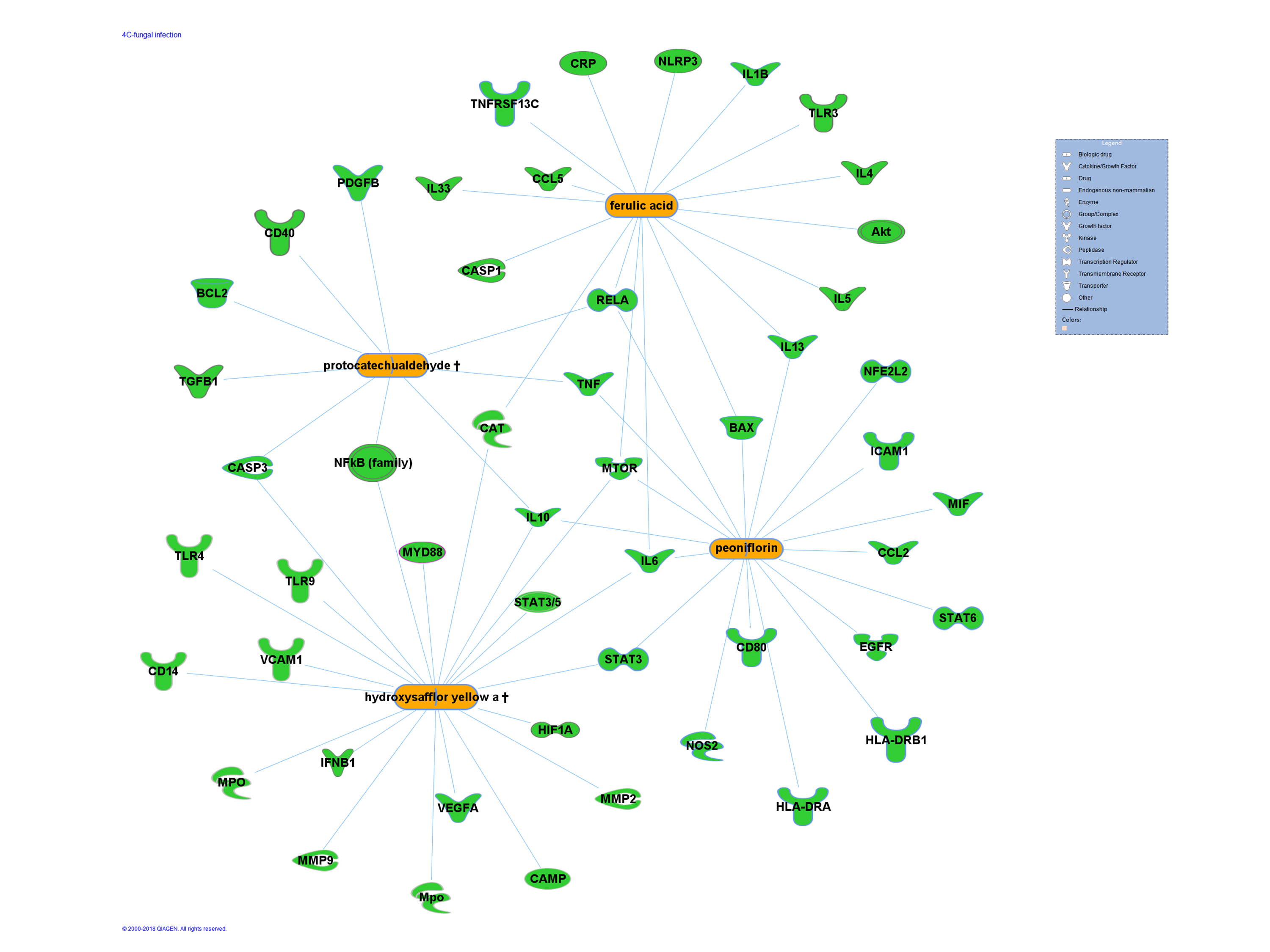

Supplement: Supplementary file 3 [file Image_2.tif]
