# Supplementary material for: Xuebijing Injection Maintains GRP78 Expression to Prevent Candida albicans–Induced Epithelial Death in the Kidney
Source: Front Pharmacol. 2020 Jan 6;10:1416. doi: 10.3389/fphar.2019.01416 (PMC6956827; doi:10.3389/fphar.2019.01416)
Supplement: Supplementary file 4 [file Table_1.pdf]

Supplementary Table 1. The list of 170 potential and proved targets of fungal infection.

ID/ Sym bol	Entrez Gene Name	Location	Type(s)	Drug(s)	Function In Fungal Infection	Related Reference
ADA	Adeno-sine deamin-ase	Cytoplas m	enzyme	pentostatin, aspirin/ dipyridamole/ telmisartan, cyclophosphamide /pentostatin/ rituximab, pentostatin/ rituximab, alemtuzumab/ pentostatin, adenosine deaminase inhibitor, dipyridamole, aspirin/ dipyridamole, pentostatin/ recombinant interferon, vidarabine	Promote inflamma-tory responses	(de Azevedo, Ferreiro et al., 2014) (Castro, Pimentel et al., 2012)
AIRE	Autoim-mune regulator	Nucleus	Transcri-ption regulator		Orchestr-ate immune response	(Cypowyj , Picard et al., 2012) (de Albuquerque et al., 2018)

AKT1	yiAKT serine/ threonine kinase 1	Cytoplas m	kinase	triciribine, GSK690693, MK2206, GSK2141795, triciribine phosphate, ipatasertib, LY2780301, ARQ092, AZD5363, BAY1125976, MPT0E028, archexin, ARQ 751, enzastaurin, perifosine	Activate Tc17 cells	(Nanjappa ,Hernande z-Santos et al., 2015) (Moyes, Shen et al., 2014)
BAX	BCL2 associate d X, apoptosis regulator	Cytoplas m	transport er		Mediate program- med cell death	(Wagner, Johnson et al., 2017) (Zhang, Huang et al., 2018)
CALC A/ PCT	Calcitoni n related polypepti de alpha	Plasma Membra ne	other		Diagnose infectious diseases	(Li, Duan et al., 2018) (Dou, Du et al., 2013)
CASP 1	Interleu- kin 1-beta convert- ase	Cytoplas m	Peptid- ase	caspase 1 inhibitor	Caspase-1 -depend- ent IL-1beta maturation	(Ganesan et al., 2014) (Ketelut- Carneiro, Ghosh et al., 2018)

CASP 3	Ice-like cysteine protease	Cytoplas m	peptidas e	caspase 3 inhibitor, emricasan	Caspase-3 dependent phagocy-tosis	(Dreschers et al., 2016) (Morais et al., 2015)
CASP 8	Caspase-8	Cytoplas m	peptidas e		Modulate dectin-1 production	(Ganesan et al., 2014) (Ketelut-Carneiro, Ghosh et al., 2018)
CAT	Catalase	Cytopla sm	enzyme	fomepizole	Upregula-tion	(Zhang, Dong et al., 2018) (Agarwal, Patel et al., 2018)
CBLB	Casitas B-lineage lymph-oma b	Nucleus	enzyme		A negative regulator for innate immunity	(Xiao et al., 2016) (Wirnsberger, Zwolanek et al., 2016)
CCL2	C-C motif chemok-ine ligand 2	Extra cellular Space	cytokine	CNTO 888, mimosine	The macropha ge-attracting chemok-ines	(Lofgren, Hullsiek et al., 2017) (Overton, Simpson et al., 2017)

CCL 20	C-C motif chemok-ine Ligand 20	Extra cellular Space	cytokine		Regulate the numbers of Th17 cells	(Hernandez-Santos, Wiesner et al., 2018) (Verma, Richardson et al., 2017)
CCL5	D-C motif chemok-ine ligand 5	Extra cellular Space	cytokine		Regulate inflamma-tory reactions	(Arango, Puerta-Arias et al., 2017) (Heung and Hohl 2016)
CCR2	C-C motif chemok-ine Receptor 2	Other	Other		Express inflamma-tory monocytes	(Hohl, Rivera et al., 2009) (Osterholzer, Chen et al., 2009)
CCR6	C-C motif chemok-ine receptor 6	Plasma Memb-rane	G-Pro-tein coupled receptor			(Verma, Richardson et al., 2017) (Alvarez, Tuen et al., 2013)
CD14	Lipopoly saccha ride(LPS) receptor	Plasma Memb-rane	Transme mbrane receptor		Recognize the Mannans	(Scriven, Graham et al., 2017) (Zawrotniak, Bochenska et al., 2017)
CD20		Plasma	other		Recognize	(Goyal,

9/ CLEC 4L/ DC-SIGN	Dendritic Cell-specific ICAM3 grabbing Non-integrin	Membr-ane			many disease-causing fungi	Castrillon-Betancur et al., 2018) (Velasquez, Stuve et al., 2018)
CD40	Tnf receptor member 5	Plasma Membr-ane	transmembrane receptor	dacetuzumab, CP-870893, RO7009789, CFZ533, FFP104, SEA-CD40, APX005M, ABBV-927	Costimul-atory molecules of dendritic cells	(Bernal, Zorro et al., 2016) (Evans, Simpson et al., 2017)
CD69/ CLEC 2C	CD69 molecule	Plasma Mem-brane	transmembrane receptor		Activation mark of CD8 T cells	(Leigh, McNulty et al., 2006) (Spec, Shindo et al., 2016)
CD80	CD80 molecule	Plasma Memb-rane	transmembrane receptor	abatacept, galiximab, belatacept, abatacept/ methotrexate	Costimu-latory molecules of dendritic cells	(Li, Fan et al., 2017) (Shi, Li et al., 2015)
CD86	CD86 molecule	Plasma Memb-rane	transmembrane receptor	abatacept, belatacept, abatacept/ methotrexate	Provide costimu-latory signals for T cell activation	(Bernal, Zorro et al., 2016) (Li, Fan et al., 2017)

CLEC 4D/ Dectin -3	C-type lectin domain family 4 member D	Plasma Memb-rane	other		Recognize alpha-mannans in the hyphae and induce NF-kappa B activation	(Zhu, Zhao et al., 2013) (Preite, Feriotti et al., 2018)
CRP	C-reactiv e protein	Extra cellular Space	other		Positive correlation with infectious degree	(Li, Duan et al., 2018) (Deng, You et al., 2013)
CSF2/ GM-C SF	Colony stimula-ting factor 2	Extra cellular Space	cytokine		Produce innate lympho-cytes	(Meester, Rosas-Tar aco et al., 2013) (Mehra, Koberle et al., 2012)
CXCL 1	D-X-C motif chemok-ine ligand 1	Extra cellular Space	cytokine		Neutrophil -attracting chemo-kines	(Supasorn ,Sringkari n et al., 2016) (Wagner, Johnson et al., 2017)
CXCL 11	D-X-C motif chemok-ine ligand 11	Extra cellular Space	cytokine		Proinfla-mmatory Reaction	(Ren, Liu et al., 2018) (Fitzpatric k, Tedrow et al.,

						2014)
IL8/ CXCL 8	C-X-C motif chemok-ine ligand 8	Extra cellular Space	cytokine		Enhance resistance to fungi	(Yeh, Horng et al., 2017) (Klaile, Muller et al., 2017)
CYP2 C19	Cytoch-rome P450 Family 2 Sub family C Member 19	Other	Other		Drug-metaboliz-ing enzyme	(Hamadeh, Klinker et al., 2017) (Lamoureux, Duflot et al., 2016)
NAD PH/ DECR 1	2,4-dienoyl-CoA reductase 1	Cytopla-sm	enzyme		Play key roles in immunity and inflamm-ation	(Hogan and Wheeler 2014) (Sanna, Caocci et al., 2017)
EGF	Epider-mal growth factor	Extra cellular Space	growth factor		Stimulate epidermal cell growth factor	(Brzozowski, Zwolinska-Wcislo et al., 2005) (Arasu, Kumaresan et al., 2016)
EGFR	Epider-mal growth factor receptor	Plasma Memb-rane	kinase	cetuximab, AEE 788, panitumumab, BMS-599626, varlitinib, tesevatinib, BMS-690514, bevacizumab/ erlotinib,	Induce human ß-Defen-sin to kill fungi	(Mehra, Koberle et al., 2012) (Wang, Wang et al., 2017)

				matuzumab, afatinib, nimotuzumab, necitumumab, BIBX 1382BS, icotinib, transforming growth factor(alpha)-pseudomonas aeruginosa exotoxin (38), MP 412, sapitinib, cetuximab/ irinotecan, CUDC 101, zalutumumab, lapatinib/ pazopanib, irinotecan/ panitumumab, erlotinib/ vismodegib, erlotinib/ gemcitabine, JNJ-26483327, lapatinib/ letrozole, capecitabine/ lapatinib, bevacizumab/ panitumumab, bevacizumab/ cetuximab, EGFR tyrosine kinase inhibitor, capecitabine/ erlotinib, lapatinib/ paclitaxel, rociletinib,		

				brigatinib,<br>MEHD7945A,<br>cabozantinib/<br>erlotinib,<br>osimertinib,<br>poziotinib,<br>olmutinib,<br>sym004,<br>IMGN289,<br>EGFR antisense<br>DNA,<br>pyrotinib,<br>EGF816,<br>naquotinib,<br>selatinib,<br>18F-PEG6-IPQA,<br>lapatinib/<br>trastuzumab,<br>ABT-414,<br>SCT200,<br>docetaxel/<br>lapatinib,<br>anti-EGFR<br>antibody,<br>AZD3759,<br>avitinib,<br>PF-06747775,<br>LY3164530,<br>RG 14620,<br>TAK-285,<br>SAR103168,<br>PF-06459988,<br>bis-tyrphostin,<br>tyrphostin A48,<br>tyrphostin A30,<br>RG 13022,<br>SYN004,<br>MM-151,<br>GC1118,<br>RM-1929,<br>hemay022,		

				hemay020, pirotinib, ABBV-221, AC0010MA, afatinib/ cetuximab, cetuximab/ encorafenib, dabrafenib/ panitumumab, BGB-283, HLX07, JNJ-61186372, cetuximab/ panitumumab, erlotinib/ gefitinib, dabrafenib/ panitumumab/ trametinib, AP32788, everolimus/ gefitinib, epitinib, depatuxizumab, CK-101, EGFR monoclonal antibody, sym013, theliatinib, doxorubicin-loaded EGFR-targeting nanocells, HS-10296, canertinib, gefitinib, cetuximab-IRDye 800CW, YH25448, BPI-15086,		

				neratinib, PD 153035, pelitinib, alflutinib, SKLB1028, lapatinib, AMG 596, vandetanib, afatinib/ paclitaxel, bevacizumab/ erlotinib/ pemetrexed, capecitabine/ cetuximab/ irinotecan, cetuximab/ vemurafenib, CPGJ602, cetuximab/ gemcitabine, doxorubicin/ lapatinib/ paclitaxel/ trastuzumab, afatinib/ osimertinib, erlotinib/		
GAP DH	Glyceraldehyde-3-phosph-ate dehydrogenase	Cytopl-asm	enzyme		Induce IL-8 and GM-CSF secretion and stimulate TLR4	(Mehra, Koberle et al., 2012) (Wagener, Schneider et al., 2013)

HIF1 A	Hypoxia inducible factor 1 alpha subunit	Nucleus	Trans-cription regulator		Regulate IL-10	(Chidambaram, Kannambath et al., 2017) (Fecher, Horwath et al., 2016)
HLA-DRA	Major histocompatibil-ity complex, class II, DR alpha	Plasma Memb-rane	Transme-mbrane receptor		Play a central role in the immune system	(Toth, Boros et al., 2017) (Elsegeiny, Marr et al.,2018)
HLA-DRB1	Major histocompatibilty complex, class II,DR beta 1	Other	Other		Play a central role in the immune system by presen-ting peptides	(Koehm, Slavin et al., 2007) (Nilsson, Lindgren et al., 2002)
HOX D13/ SPD	Homeo-box D13	Nucleus	Transcri-ption regulator		Is relevant in the host defense against fungi	(Pandit, Madhukaran et al., 2012) (Geunes-Boyer, Beers et al.,2012)
ICAM 1	Inter cellular adhesion molecule 1	Plasma Memb-rane	Transm-embrane receptor		Regulate the body's immune response	(Zhang, Sun et al., 2016) (Quaresma, Brito et al., 2015)

IDO1	Indolea-mine 2,3-diox-ygenase 1	Cytopl-asm	enzyme	1-methyl-D-2-tryptophan, 3-epacadostat, NLG919, KHK2455, PF-06840003, BMS-986205, LY3381916	IDO-AhR Axis Controls Th17/Treg Immunity	(de Araujo, Feriotti et al., 2017) (Zelante, Pieraccini et al., 2016)
IFNB/ IFNB 1	Interferon beta 1	Extra cellular Space	cytokine		Interferon-beta in dendritic cells is crucial for immunity	(Mehra, Koberle et al., 2012) (del Fresno, Soulat et al., 2013)
IL1	Interleuki n-1	Extra cellular Space	group	rilonacept	Regulate immune response	(Mehra, Koberle et al., 2012) (Smith, Hankinso n et al., 2014)
IL12R B1	Interleu-kin 12 receptor subunit beta 1	Plasma Memb-rane	Transm-embrane receptor		Is essential for protection against intra-Macroph-agic pathogens	(Lilic 2012) (Huppler, Bishu et al., 2012)

IL13	Interleu-kin 13	Extra cellular Space	cytokine	tralokinumab	Induce type 2 cytok-ines,	(Meester, Rosas-Taraco et al., 2013) (Piehler, Eschke et al., 2016)
IL15	Interle-ukin 15	Other	Other		Have potential for stimula-ting anti-tumor Tlympho-cytes and NK cells	(Dhalla, Fox et al., 2016) (Smith, Hankinson et al., 2014)
IL17A	Interle-ukin 17A	Extra cellular Space	cytokine	secukinumab, ixekizumab, CJM112, ABY-035, bimekizumab, methotrexate/ secukinumab, BCD-085, ixekizumab/ methotrexate	Innate lymphocyte-derived IL-17A enable phagocyte-driven fungal killing	(Ahlgren, Moretti et al., 2011) (Garth, Reeder et al., 2017)
IL1-α	Interle-ukin 1 alpha	Extra cellular Space	cytokine	MABp1	Regulate nTh17 cells	(Garth, Reeder et al., 2017) (Mehra, Koberle et al., 2012)

IL1B	Interle-ukin 1 beta	Extra cellular Space	cytokine	canakinumab, gevokizumab, canakinumab/INS, gallium nitrate	Innate immune inflamm-atory factor	(Zheng, van de Veerdonk et al., 2015) (Chidambaram, Kannambath et al., 2017)
IL1R1 / IL1R	Interle-ukin 1 receptor type 1	Plasma Memb-rane	Transm-embrane receptor	anakinra	Induce Th2 immune response	(Hernandez-Santos, Wiesner et al., 2018) (Nanjappa ,Hernandez-Santos et al., 2015)
IL27	Interle-ukin 27	Extra cellular Space	cytokine		Promote improved antigen processing and stimula-tion of T cells	(Patin, Jones et al., 2016) (Depner, Fuchs et al., 2016)
IL33	Interle-ukin 33	Extra cellular Space	cytokine		Is a potent inducer of Th2 immune response	(Garth, Reeder et al., 2017) (Piehler, Eschke et al., 2016)
IL5	Interle-ukin 5	Extra cellular Space	cytokine	mepolizumab, reslizumab	Is Th2 cytokines	(Scriven, Graham et al., 2017) (Wanachiwanawin, Mendoza

						et al., 2004)
IFNA	Interferon α	Extra cellular Space	group		Activate immune response	(del Fresno, Soulat et al., 2013) (Depner, Fuchs et al., 2016)
IRF1	Interferon regulat-ory factor 1	Nucleus	Transcri-ption regulator		Proinfla-mmatory transcrip-tion factor	(Radovanovic, Leung et al., 2014) (Wevers, Kaptein et al., 2014)
ERK2 / MAP K1	Mitogen-activated protein kinase 1	Cytoplasm	kinase	MAP kinase1 inhibitor, binimetinib, ulixertinib, LY-3007113, pexmetinib, LY3214996, LTT462, KO-947, ASN007, ASTX029	ERK mitogen-activated protein kinase-dependent is important for host defence	(Chen, Ji et al., 2016) (Perez-Nadales and Di Pietro 2011)
MAP K P38/14	Mitogen-activated protein kinase 14	Cytoplasm	kinase	talmapimod, RO-3201195, ralimetinib, p38 MAP kinase inhibitor	A central signaling for Th17 different-iation and inflamm-ation	(Higuchi 2015) (Bielska, Higuchi et al., 2014)
ERK1 / MAP K3	Mitogen-activated protein kinase 3	Cytoplasm	kinase	ulixertinib, LY3214996, KO-947, ASN007,	Is important for host defence	(Chen, Ji et al., 2016)

				ASTX029		(Selander, Engblom et al., 2009)
MAP K8/ JNK1	Mitogen-activated protein kinase 8	Cytoplas m	kinase	encorafenib, cetuximab/ encorafenib, binimetinib/ encorafenib, aplidine	Suppress CD23 expression	(Carpino, Naseem et al., 2017) (Zhao, Guo et al., 2017)
MBL/ MBL2	Mannose binding lectin 2	Extra cellular Space	Peptidas e		Identify various pathogenic organisms	(Osthoff, Wojtowic z et al., 2016) (Frakking , Israels et al., 2011)
MIF	Macro-phage migration inhibitory factor	Extra cellular Space	cytokine	imalumab	A cytokine with multiple -effect inflamm-atory mediation	(Hu, Hu et al., 2014) (Mirkov, Belij et al., 2012)
MMP 2	Matrix metallo peptidase 2	Extra cellular Space	peptidas e	MMP2 MMP9 inhibitor, rebimastat, marimastat, prinomastat	Modulate extracell-ular matrix turnover, inflamm-ation and immunity	(Hajari Taheri, Seyedolm ohadesin et al., 2013) (Zhan, Zhang et al., 2016)
MMP 9	Matrix metallo peptidase 9	Extra cellular Space	peptidas e	MMP2 MMP9 inhibitor, GS-5745, rebimastat, marimastat, prinomastat, glucosamine	Modulate extracell-ular matrix turnover, inflamm-ation and immunity	(Hajari Taheri, Seyedolm ohadesin et al.,2013) (Taylor,

						Roy et al., 2016)
MTOR	Mechanistic target of rapamycin kinase	Nucleus	kinase	ridaforolimus, OSI-027, methotrexate/ sirolimus/ tacrolimus, dactolisib, AZD8055, vistusertib, PF-4691502, apitolisib, PKI-179, everolimus/ exemestane, lenalidomide/ temsirolimus, gedatolisib, imatinib/ sirolimus, cyclosporine A/sirolimus/ tacrolimus, everolimus/ prednisone, everolimus/ sorafenib, everolimus/ gefitinib, everolimus/ lenvatinib, sirolimus, temsirolimus, tacrolimus, Mtor inhibitor/ tyrosine kinase inhibitor, everolimus/ tamoxifen,	Modulate CD8+ T-cell differentiation	(Cui, Wang et al., 2017) (Nanjappa ,Hernandez-Santos et al., 2015)

				everolimus, everolimus/ fulvestrant, everolimus/ letrozole, prednisone/ tacrolimus, pimecrolimus		
MUC 7	Mucin 7, secreted	Cytoplas m	other		Participate in innate immune responses	(Intini, Aguirre et al., 2003) (Satyanar ayana, Situ et al., 2000)
NRF2 / NFE2 L2	Nuclear factor, erythroid 2 like 2	Nucleus	Transcr- iption regulator	RTA 408	Have a possible role in neutrophil survival	(Araujo, Demasi et al., 2013) (Ishida, Ohta et al., 2018)
NLR	NOD-like receptors	Cytoplas m	group		Contribute to the recogn- ition of a vast range of fungi	(Plato, Hardison et al., 2015) (Malired di and Kannegan ti 2013)
NLRP 3	NLR family pyrin domain contain- ing 3	Cytoplas m	other		Trigger caspase-1- dependent IL-1beta maturation	(Ganesan, Rathinam et al., 2014) (Ketelut- Carneiro, Ghosh et al.,2018)
iNOS/ NOS2	Nitric oxide	Cytoplas m	enzyme	pimagedine, triflusal,		(Meester, Rosas-Tar

	synthase 2			GW 273629, N-methyl-L-arginine	Pro-infla-mmatory marker	aco et al., 2013) (Morgado ,Schubach et al., 2011)
NOS3	Nitric oxide synthase 3	Cytoplas m	enzyme	5,6,7,8-Tetrahydro-biopterin, GW 273629, N-methyl-L-O-arginine	A biologic mediator of antimi-crobial and antitum-oral activity	(Sellathamby, Lakshmi et al., 2012) (Liu, Spellberg et al., 2010)
PDGF B	Platelet derived growth factor subunit B	Extra cellular Space	growth factor	pegpleranib	Regulate cell migration/ prolifer-ation and survival	(Liu, Shetty et al.,2015) (Zheng, van de Veerdonk et al., 2015)
PTGS 2/ COX2	Prostag-landin-endope-roxide synthase 2	Cytoplas m	enzyme	acetaminophen/ pentazocine, acetaminophen/ clemastine/ pseudoephedrine, aspirin/ butalbital/ caffeine, acetaminophen/ caffeine/ dihydrocodeine, aspirin/ hydrocodone, aspirin/oxycodone, acetaminophen/ aspirin/caffeine, aspirin/ pravastatin,	Involve in inflamm-ation and mitogen-esis	(Hua, Chi et al., 2017) (Garth, Reeder et al., 2017)

				acetaminophen/ aspirin/ meprobamate, aspirin/ caffeine/ propoxyphene, butalbital/ caffeine/codeine, dihydrocodeine, chlorpheniramine/ ibuprofen/ pseudoephedrine, licofelone, menatetrenone, icosapent, suprofen, lornoxicam, tiaprofenic acid, lumiracoxib, tenoxicam, naproxen/ sumatriptan, apricoxib, parecoxib, ibuprofen/ phenylephrine, acetaminophen/ codeine, esomeprazole/ naproxen, famotidine/ ibuprofen, dabigatran etexilate, diclofenac/ omeprazole, chlorpheniramine/ Ibuprofen flurbiprofen, phenacetin, sulindac,		

				nabumetone, etodolac, tolmetin, ketorolac, oxaprozin, mesalamine, fenoprofen, salicylic acid, acetaminophen/ caffeine/ chlorpheniramine/ hydrocodone/ phenylephrine, bromfenac		
NFkB / RELA	RELA proto-oncogene / NF-kB subunit	Nucleus	Transcr-iption regulator	NF-kappaB decoy	Is important protective signalling pathway for C. albicans	(Radovanovic, Leung et al., 2014) (Wang, Wang et al.,2017)
RNASE7	Ribon-Uclease A family member 7	Extra cellular Space	enzyme		Have broad-spectrum antimi-crobial activity	(Mehra, Koberle et al., 2012) (Petrucelli, Peronni et al., 2018)
SFTPA1/ SP-A	Surfact-ant protein A1	Extra cellular Space	Transp-orter		Play an essential role in the defense against respirat-ory pathogens	(Saxena, Madan et al., 2003) (Pandit, Madhukaran et al., 2012)
SGSM3/ MAP	Small G protein signaling modu-lator 3	Plasma Membrane	other		Involved in ectoderm different-	(Brunke, Quintin et al., 2015) (Yamauchi,Takayan

					iation	agi et al.,2004)
SOCS 1	Suppre-ssor of cytokine signaling 1	Cytoplas m	other		Promote the maturation of DCs	(Shi, Li et al., 2018) (Shi, Li et al., 2015)
SOD1	Supero-xide dismutase 1	Cytoplas m	enzyme		Its related pathway is Amyotr-ophic lateral-sclerosis	(Zhang, Dong et al., 2018) (Hua, Chi et al., 2017)
STAT 3	Signal transd-ucer and activator of transcri-ption 3	Nucleus	Transcri-ption regulator	OPB-31121, OPB-51602, STAT3 inhibitor XIII, danvatirsen	Trigger production of Th-17 cytok-ines	(Zheng, van de Veerdonk et al., 2015) (Taylor, Roy et al., 2016)
STAT 6	Signal transdu-cer and activator of transcript ion 6	Nucleus	Transcri-ption regulator		Regulate natural helper cell prolifera-tion	(Doherty, Khorram et al., 2012) (Bloodwo rth, Newcomb et al., 2016)
SULT 2A1/ ST2	Sulfotr-ansferase family 2A member 1	Cytoplas m	enzyme		Promote T helper 2 cell activation	(Piehler, Eschke et al., 2016) (Rostan, Arshad et al., 2015)
SYK	Spleen	Cytoplas	kinase	R-348,		

	associa-ted tyrosine kinase	m		fostamatinib, cerdulatinib, TAK-659, entospletinib, HMPL-523, GSK2646264, GS-9876	Involve in M.canis-induced IL-1beta secretion	(Mao, Zhang et al., 2014) (Kottom, Hebrink et al., 2018)
TGFB 1/ TGFB	Transf-orming growth factor beta 1	Extra cellular Space	growth factor	dalantercept, fresolimumab, LY3200882, MSB0011359C	Anti-infla mmatory factor	(Morais, Martins et al., 2015) (Sawada, Nakamura et al., 2012)
TLR3	Toll like receptor 3	Plasma Membr-ane	Transm-embrane receptor	rintatolimod	Pathogen recognitio n receptor on the surfaces of immune cells	(Overton, Simpson et al., 2017) (Smith, Hankinso n et al., 2014)
TLR9	Toll like receptor 9	Plasma Membr-ane	Transm-embrane receptor	agatolimod, ISS-1018, HYB-2055, GNKG168, SD-101, MGN1703, DUK-CPG-001, hydroxychlor-oquine, IMO-2125, DV281, CPG 10104	Pathogen recogn-ition receptor on the surfaces of immune cells	(Mehra, Koberle et al., 2012) (Vieira, Fernandes et al., 2017)
TRAF 6	TNF receptor associa-ted factor 6	Cytoplas m	enzyme		Play essential roles in the CLRs	(Smith, Hankinso n et al., 2014) (Gorjesta ni, Darnay

						et al.,2012)
VEGF A	Vascular endothe-lial growth factor A	Extra cellular Space	growth factor	dalteparin, bevacizumab, ranibizumab, aflibercept, bevacizumab/ erlotinib, bevacizumab/ sorafenib, bevacizumab/ 5-fluorouracil, bevacizumab/ temozolomide, bevacizumab/ irinotecan, bevacizumab /carmustine/ lomustine, bevacizumab/ paclitaxel, Bevacizumab /irinotecan/ oxaliplatin, aflibercept/ irinotecan, bevacizumab/ capecitabine/ oxaliplatin, bevacizumab/ panitumumab, bevacizumab/ cetuximab, bevacizumab/ pemetrexed, bevacizumab/ gemcitabine, bevacizumab/ capecitabine/ irinotecan/ oxaliplatin,	A key mediator of angiogen-esis	(Lupianez , Canet et al., 2015) (Smith, Hankinso n et al., 2014)

				bevacizumab/ capecitabine, bevacizumab/ paclitaxel/ topotecan, bevacizumab/ oxaliplatin, bevacizumab/ doxorubicin/ bevacizumab/ doxorubicin, vanucizumab, pegaptanib, bevacizumab/ Capecitabine /irinotecan, bevacizumab/ erlotinib/ pemetrexed, bevacizumab/ paclitaxel/ pemetrexed, bevacizumab/ etoposide, aflibercept/ bevacizumab, bevacizumab/ bevacizumab/ cetuximab/ erlotinib, atezolizumab/ bevacizuma, chelerythrine		
VCAM1	Vascular cell adhesion molecule 1	Plasma Memb-rane	Transm-embrane receptor		TNF-alpha Increase expression of VCAM-1	(Hajari Taheri, Seyedolmohadesin et al., 2013) (Qureshi, Cook-Mil

						ls et al., 2003)
MMP 3	Matrix metallopeptidase 3	Extra cellular Space	peptidase	marimastat	Promote effective host immun-ity	(Supasorn ,Sringkarin et al., 2016) (Hajari Taheri, Seyedolmohadesin et al., 2013)
CAMP	Cathelic-idin antimi-crobial peptide	Cytoplasm	other		Encode a member of an antimicro-bial peptide	(Liu, Yang et al., 2018) (Mehra, Koberle et al., 2012)
Mincle/ CLEC4E	C-type lectin domain family 4 member E	Plasma Membr-ane	other		Play a role in the recognit-ion of pathogenic fungi	(Xiao, Tang et al., 2016) (Zhao, Xu et al., 2017)
MPO	Myeloperoxidase	Cytoplasm	enzyme		Produce hypohal-ous acids central to the microbici-dal activity of neutroph-ils	(Hu et al., 2014) (Mirkov et al., 2012)
MYD88	Myeloid different-iation primary	Plasma Memb-rane	other	IMO-8400	An essential signal transducer	(Arnold-Schrauf et al., 2015) (Nanjappa

	response 88				in the IL1 and Toll-like receptor signaling pathways	et al., 2015)
BCL2	B cell leukae-mia/ lympho-ma 2	Cytoplas m	transport er	oblimersen, rasagiline, (-)-gossypol, obatoclax, BCL-2 blocker, navitoclax, gemcitabine /paclitaxel, bortezomib/ paclitaxel, venetoclax, paclitaxel/ trastuzumab, paclitaxel/ pertuzumab/ trastuzumab, lapatinib/ paclitaxel, doxorubicin/ paclitaxel, epirubicin/ paclitaxel, paclitaxel/ ramucirumab, paclitaxel/ topotecan, BCL201, S 055746, APG-1252, rituximab/ venetoclax, paclitaxel/ rituximab, afatinib/ paclitaxel,	Block the apoptotic death of some cells such as lymphoc-ytes	(Nanjappa et al., 2015) (Wagner et al., 2017)

				doxorubicin/ lapatinib/ paclitaxel/ trastuzumab, paclitaxel, chelerythrine		
CARD9	Caspase recruit-ment domain family member 9	Cytoplasm	other		Involve in M. canis-induced IL-1beta secretion	(Ishii and Kawakami, 2014) (Mao et al., 2014)
Dectin-2/ CLEC 6A	C-type lectin domain containing 6A	Plasma Membrane	Transm-embrane receptor		Participate in the initial innate immune signaling response	(Nakamura et al., 2015) (Kottom et al., 2018)
Dectin-1/ CLEC 7A	D-type lectin domain contain-ing 7A	Plasma Memb-rane	transmembrane receptor		Participate in the initial innate immune signaling response	(Mao et al., 2014) (Overton et al., 2017)
IFNG	Interferon gamma	Extra cellular Space	cytokine		Secreted by cells of both the innate and adaptive immune systems	(Bernal et al., 2016) (de Azevedo et al., 2014)
IL10	Interleu-kin 10	Extra cellular Space	cytokine		Immuno-regulation	(Zheng et al., 2015) (Fecher et al., 2016)

IL12 (complex)	Interleukin-12	Extra cellular Space	complex	ustekinumab, methotrexate/ ustekinumab	Promote the different-iation of T lympho-cytes and NK cells	(Bernal et al., 2016) (Li et al., 2017)
IL12 (family)	Interleukin 12	Extra cellular Space	group		Regulate cellular immunity	(Hu et al., 2014) (Lupianez et al., 2015)
IL2	Interleukin 2	Extra cellular Space	cytokine		Important for the proliferat-ion of T and B lymphoc-ytes	(Schulze et al., 2016) (Michelsen et al., 2017)
IL22	Interleukin 22	Extra cellular Space	cytokine		IL-10-Related T-Cell-Derived inducible factor	(Ahlgren et al., 2011) (Dhalla et al., 2016)
IL6	Interleukin 6	Extra cellular Space	cytokine	tocilizumab, siltuximab, clazakizumab	Involve in inflamm-ation and maturation of B cells	(Bernal et al., 2016) (Garth et al., 2017)
IL4	Interleukin 4	Extra cellular Space	cytokine		Related pathwa-ys are Akt and T cell receptor	(Hu et al., 2014) (Ishii and Kawakami, 2014)

JDP2	Jun dimeri-zation protein 2	Nucleus	Transcri-ption regulator		Its related pathways are Tacrolimus/Cyclo-sporine Pathway	(Maruyama et al., 2017) (Maruyama et al., 2012)
STAT 1	Signal transd-ucer and activator of transcrip-tion 1	Nucleus	Transcri-ption regulator		Regulate immune polarizat-ion and activate macroph-ages	(Zheng et al., 2015) (Radovanovic et al., 2014)
TLR2	Toll like receptor 2	Plasma Memb-rane	Transm-embrane receptor	OM 174 lipid	Pathogen recogni-tion receptor on the surfaces of immune cells	(Chidambaram et al., 2017) (Dreschers et al., 2016)
TLR4	Toll like receptor 4	Plasma Memb-rane	Transm-embrane receptor	eritoran, resatorvid, OM 174 lipid	Pathogen recognit-ion receptor on the surfaces of immune cells	(Chidambaram et al., 2017) (Ketelut-Carneiro et al., 2015)
TNF/ TNF-α	Tumor necrosis factor	Extra cellular Space	cytokine	adalimumab, etanercept, infliximab, certolizumab, golimumab, tumor necrosis factor receptor antagonist, infliximab/ methotrexate,	A multifun-ctional proinfla-mmatory cytokine	(Chidambaram et al., 2017) (Qureshi et al., 2003)

				dexamethasone/ thalidomide, dexamethasone/ pomalidomide, cyclophosphamide /dexamethasone/ thalidomide, golimumab/ methotr-exate, bortezomib/ dexamethasone/ thalidomide, rituximab /thalidomide, bortezomib/ thalidomide, prednisone/ thalidomide, adalimumab/ methotrexate, etanercept/ methotrexate, pomalidomide, bortezomib/ dexamethasone/ pomalidomide, thalidomide, infliximab/ Methylpredniso-lone		
CXCL 2	C-X-C motif chemok-ine ligand 2	Extra cellular Space	cytokine		Involve in immuno-regulatory and inflamm-atory	(Dong et al., 2017) (Zhao et al., 2017)
BCL-XL/ BCL2 L1	BCL2 like 1	Cytoplas m	other		anti-apoptotic molecule	(Wang et al., 2016) (Zhou, Shen et al., 2010)

CCR4	D-C motif chemok-ine receptor 4	Plasma Memb-rane	G-protein coupled receptor	mogamulizumab	Control the Effects of regulat-ory T cells	(Bao, Jin et al., 2013) (Havel, Wool et al., 2011)
CYP17A1	Cytoch-rome P450 family 17 subfamily A member 1	Cytoplasm	enzyme	abiraterone acetate, orteronel, abiraterone, CFG-920, seviteronel, abiraterone/ prednisolone, ketoconazole, CYP17A1 inhibitor	Involve in drug metabo-lism and synthesis of cholesterol	(http://www.ingenuity.com)
DHFR	Dihydro-folate reductase	Nucleus	enzyme	pyrimethamine, trimethoprim, iclaprim, proguanil, methotrexate/ ofatumumab, methotrexate/ sirolimus/ tacrolimus, pralatrexate, abatacept/ methotrexate, infliximab/ methotrexate, piritrexim, methotrexate/ rituximab, golimumab/ methotrexate, cisplatin/ doxorubicin/ methotrexate, cytarabine/ methotrexate,	Have a key role in cell growth and prolifer-ation	(Mane, Gujar et al., 2015) (Munoz, Zuluaga et al., 2012)

				cisplatin/ doxorubicin/ ifosfamide/ methotrexate, methotrexate/ rituximab/ temozolomide, ifosfamide/ methotrexate, bevacizumab/ pemetrexed, cytarabine/ dexamethasone/ methotrexate, leucovorin/ methotrexate, cyclophosphamide /methotrexate, adalimumab/ methotrexate, gemcitabine/ pemetrexed, epinephrine/ methotrexate, dihydrofolate reductase inhibitor, ABTL0812, methotrexate/ tofacitinib, etanercept/ methotrexate, methotrexate/ ustekinumab, baricitinib/ methotrexate, aminopterin, methotrexate/ tocilizumab, methotrexate/ sarilumab,		

				cyclophosphamide /methotrexate/ trastuzumab, methotrexate, atovaquone/ proguanil, bevacizumab/ erlotinib/ pemetrexed, bevacizumab/ paclitaxel/ pemetrexed, arsenictrioxide/ cytarabine/ methotrexate, 6-mercaptopurine/ methotrexate/ tretinoin, methotrexate/ secukinumab, cytarabine/ dasatinib/ dexamethasone/ methotrexate, cytarabine/ dexamethasone/ imatinib/ methotrexate, cytarabine/ methotrexate/ mitoxantrone, Cytarabine/ imatinib/ methotrexate, ixekizumab/ methotrexate, sulfisoxazole, triamterene, pembrolizumab/ pemetrexed, folic acid,		

				trimetrexate, pemetrexed, talotrexin		
GABRA3	Gamma-Aminobutyric acid type A receptor alpha3 subunit	Plasma Membrane	ion channel	methohexital, primidone, meprobamate, aspirin/ butalbital/ caffeine,aspirin/ butalbital/ caffeine/ codeine, hexobarbital, pagoclone, alphadolone, SEP 174559, heptabarbital, zopiclone, clobazam, nitrazepam, adinazolam, butobarbital, metyrapone/ oxazepam, acetaminophen/ butalbital/ caffeine, sevoflurane, isoflurane, gaboxadol, isoniazid, dexamethasone/ olanzapine, felbamate, etomidate, muscimol, halothane, fluoxetine/ olanzapine, amobarbital, estazolam,	The major inhibitory neurotransmitter in the mammalian brain	(http://www.ingenuity.com)

				atropine/ hyoscyamine/ phenobarbital/ scopolamine, clorazepate, acetaminophen/ butalbital, eszopiclone, quazepam, mephobarbital, hyoscyamine/ Phenobarbital, amitriptyline/ chlordiazepoxide, acetaminophen/ butalbital/caffeine/ codeine, butabarbital, diazepam, temazepam, zolpidem, chlordiazepoxide, lorazepam, olanzapine, triazolam, clonazepam, flurazepam, midazolam, flunitrazepam, oxazepam, alprazolam, zaleplon, thiamylal, secobarbital, barbital, butalbital, phenobarbital, pentobarbital, thiopental, ezogabine, desflurane,		

				methoxyflurane, enflurane, pregnenolone		
GABRA6	Gamma-Aminobu-tyric acid type A receptor alpha6 subunit	Plasma Memb-rane	ion channel	methohexital, primidone, meprobamate, aspirin/ butalbital/ caffeine,aspirin/ meprobamate, aspirin/butalbital/ caffeine/codeine, hexobarbital, pagoclone, alphadolone, SEP 174559, heptabarbital, clobazam, nitrazepam, butobarbital, metyrapone/ oxazepam, acetaminophen/ butalbital/caffeine, sevoflurane, isoflurane, gaboxadol, isoniazid, dexamethasone/ olanzapine, felbamate, etomidate, muscimol, halothane, fluoxetine/ olanzapine, amobarbital, atropine/ hyoscyamine/ phenobarbital/	Related pathways are Akt Signalinga ndGABA receptor activation	(http://www.ingenuity.com)

				scopolamine, acetaminophen/ butalbital, eszopiclone, mephobarbital, hyoscyamine/ phenobarbital, acetaminophen/ butalbital/caffeine/ codeine, butabarbital, diazepam, temazepam, zolpidem, lorazepam, olanzapine, triazolam, clonazepam, flurazepam, midazolam, flunitrazepam, oxazepam, zaleplon, thiamylal, secobarbital, barbital, butalbital, phenobarbital, thiopental, ezogabine, desflurane, methoxyflurane, enflurane, pregnenolone		
GABRB1	Gamma-Aminobutyric acid type A receptor beta1	Plasma Membrane	ion channel	methohexital, aspirin/butalbital/ caffeine, aspirin/butalbital/ caffeine/codeine, pagoclone, alphadolone,	Mediate the fastest inhibitory synaptic transmission in the central	(http://www.ingenuity.com)

	subunit			SEP 174559, nitrazepam, adinazolam, metyrapone/ oxazepam, acetaminophen/ butalbital/caffeine, sevoflurane, 4-hydroxybut-anoic acid, isoflurane, gaboxadol, lindane, isoniazid, dexamethasone/ olanzapine, felbamate, etomidate, muscimol, halothane, fluoxetine/ olanzapine, amobarbital, estazolam, atropine/ hyoscyamine/ phenobarbital/ scopolamine, acetaminophen/ butalbital, eszopiclone, quazepam, mephobarbital, hyoscyamine/ phenobarbital, acetaminophen/ butalbital/caffeine/ codeine, butabarbital, diazepam, temazepam,	nervous system	

				zolpidem,<br>lorazepam,<br>olanzapine,<br>triazolam,<br>clonazepam,<br>flurazepam,<br>midazolam,<br>oxazepam,<br>zaleplon,<br>secobarbital,<br>butalbital,<br>phenobarbital,<br>pentobarbital,<br>thiopental,<br>ezogabine,<br>desflurane,<br>methoxyflurane,<br>enflurane,<br>pregnenolone		
GABRE	Gamma-Aminobutyric acid type A receptor epsilon subunit	Plasma Membrane	ion channel	pagoclone,<br>alphadolone,<br>SEP 174559,<br>nitrazepam,<br>tracazolate,<br>adinazolam,<br>metyrapone/<br>oxazepam,<br>sevoflurane,<br>isoflurane,<br>gaboxadol,<br>isoniazid,<br>dexamethasone/<br>olanzapine,<br>felbamate,<br>etomidate,<br>muscimol,<br>halothane,<br>fluoxetine/<br>olanzapine,<br>estazolam,<br>eszopiclone,	Its related pathways are Akt signaling and GABAergic synapse	(http://www.ingenuity.com)

				quazepam, diazepam, temazepam, zolpidem, lorazepam, olanzapine, triazolam, clonazepam, flurazepam, midazolam, oxazepam, zaleplon, secobarbital, phenobarbital, pentobarbital, ezogabine, desflurane, methoxyflurane, enflurane, pregnenolone		
GIT2	G Protein-Coupled Receptor Kinase-Interactor 2	Nucleus	other		Regulate cell motility	(http://www.ingenuity.com)
GPR34	G protein-coupled receptor 34	Plasma Memb-rane	G-protein coupled receptor		Result in physio-logic response	(http://www.ingenuity.com)
IFNGR1	Interferon gamma receptor 1	Plasma Memb-rane	Transm-embrane receptor	interferon gamma-1b	Involve in Bacterial infections in CF airways and HIF-1 signaling pathway	(http://www.ingenuity.com)
IFNG	Interferon	Plasma	Transm-	interferon	Involve in	(http://ww

R2	gamma receptor 2	Memb-rane	embrane receptor	gamma-1b	Bacterial infectiona nd HIF-1 signaling pathway	w.ingenui ty.com)
IgG1	Immuno-globulin	Other	complex		Produce by lympho-cytes of the immune system	(Ribeiro, Souza et al., 2013) (Casadeva ll, Pirofski et al., 2012)
KRT1 4	50KDA type I epidermal keratin	Cytoplas m	other		Related pathways are Glucocorti coid receptor regulatory network	(Chang, van der et al., 2012) (http://ww w.ingenui ty. com)
MMP 19	Matrix metallope ptidase 19	Extra cellular Space	peptidas e	marimastat	Related pathways are Integrin Pathway and Degrada-tion of the extra cellular matrix	(Knorr, Schmidtb erg et al., 2009) (http://ww w.ingenui ty. com)

TRAF 3IP2	TRAF3 interact-ing protein 2	Cytoplas m	other		Play a central role in innate immunity in response to pathogens	(http://www.ingenuity.com)
TYK2	Tyrosine kinase 2	Plasma Membra ne	kinase	ruxolitinib, PF-06700841	Play a role in anti-viral immun-ity	(Conti, Whibley et al., 2015) Dejima, Shibata et al., 2011)
AKN A	AT-hook transcri-ption factor	Nucleus	Transcri-ption regulator		Activate the express-ion of the CD40 receptor	(http://www.ingenuity.com)
ANX A1	Annexin A1	Plasma Memb-rane	enzyme	hydrocortisone, hydrocortisone/ prednisone, hydrocortisone/ mitoxantrone	Have anti-infla mmatory activity	(Higurashi, Arai et al., 2007) (http://www.ingenuity.com)
ATP1 A1	ATPase Na+/K+ transport-ing subunit alpha 1	Plasma Memb-rane	transport er	digoxin, acetyldigitoxin derivative, ethacrynic acid, reserpine/trichloro methiazide, bretylium, perphenazine, ouabain, digitoxin	Establish and maintain the electro-chemical gradients of Na and K ions	(Conti, Whibley et al., 2015) (Milner, Sandler et al., 2010)

ATP4 A	ATPase H+/K+ transporting alpha subunit	Plasma Memb- rane	Transp- orter	ilaprazole, tenatoprazole, AGN201904, AR-H047108, dexlansoprazole, magnesium hydroxide/ omeprazole/ sodium bicarbonate, omeprazole/ sodium bicarbonate, esomeprazole/ naproxen, aspirin/ esomeprazole, diclofenac/ omeprazole, aspirin/ omeprazole, clarithromycin/ omeprazole, esomeprazole magnesium, omeprazole, lansoprazole, rabeprazole, pantoprazole	Encode a catalytic alpha subunit of the gastric H+, K+- ATPase	(http://www.ingenuity.com)
CCR1	C-C motif chemokine receptor 1	Plasma Memb- rane	protein coupled receptor		Is critical for the recruit- ment of effector immune cells	(Carpenter, Ewing et al., 2005) (Lionakis, Fischer et al., 2012)

CCR5	C-C motif chemokine receptor 5	Plasma Membrane	G-protein coupled receptor	maraviroc, vicriviroc, ancriviroc, BMS-813160	An important co-receptor for macrophage tropic virus	(Kroetz, Deepe et al., 2010). (Kroetz, Deepe et al., 2011)
CEBPB	CCAAT/ enhancer binding protein beta	Nucleus	Transcription regulator		Is important in the regulation of genes involved in immune and inflammatory response	(Hirai, Zhang et al., 2006) (Hsu, Sampaio et al., 2011)
CERS3	Ceramide synthase 3	Nucleus	Transcription regulator		Maintain epidermal lipid homeostasis and terminal differentiation	(http://www.ingenuity.com)
CXCR2	E-X-C motif chemokine receptor 2	Plasma Membrane	G-protein coupled receptor	repertaxin, SB-265610, navarixin, AZD5069, 2'-((4'-trifluoromethanesulfonyloxy)phenyl)-N-methanesulfonylpropionamide	Mediate neutrophil migration to sites of inflammation	(http://www.ingenuity.com)

CYB B	Cytoch-rome b-245 beta chain	Cytoplas m	enzyme		A primary compon-ent of the microbi-cidal oxidase system of phago-cytes	(Bustamante, Boisson-Dupuis et al., 2014) (Watkins, Saleh et al., 2012)
CYP3 A4	Cytoch-rome P450 family 3 subfamily A member 4	Cytoplas m	enzyme	cobicistat, cobicistat/ elvitegravir/ emtricitabine/ tenofovir disoproxil, atazanavir/ cobicistat, cobicistat/ darunavir, ketoconazole	Involve in the metabo-lism of approxi-mately half the drugs	(Hoekstra, Garvey et al., 2014) (Lestner, Hope et al., 2013).
CYP5 1A1	Cytoch-rome P450 family 51 subfamily A member 1	Cytoplas m	enzyme	posaconazole, luliconazole, efinaconazole, betamethasone/ clotrimazole, econazole, itraconazole, clotrimazole, fluconazole, miconazole, bifonazole, ketoconazole, sertaconazole, oxiconazole, terconazole, voriconazole	Catalyze many reactions involved in drug metabo-lism and synthesis of cholesterol	(Khan, Sabbah et al., 2018) (Morio, Loge et al., 2010)
Defb3 /DEF B103 A(B)	Defensin beta 103B	Extra cellular Space	other		Form a family of microbi-cidal and cytotoxic	(Fukuda, 2013) (Fernandez-Silva,

					peptides made by neutro-phils	Capilla et al., 2014)
GAB RA1	Gamma-Aminob-utyric acid type A receptor alpha1 subunit	Plasma Memb-rane	ion channel	methohexital, primidone, meprobamate, aspirin/ butalbital/ caffeine,aspirin/ meprobamate, aspirin/butalbital/ caffeine/codeine, hexobarbital, pagoclone, ethchlorvynol, alphadolone, SEP 174559, heptabarbital, zopiclone, glutethimide, clobazam, nitrazepam, phentermine/ topiramate, caffeine/ethanol, adinazolam, butobarbital, metyrapone/ oxazepam, acetaminophen/ butalbital/caffeine, sevoflurane, isoflurane, gaboxadol, isoniazid, dexamethasone/ olanzapine, felbamate,ethanol, etomidate, muscimol,	Its related pathways are Akt Signaling and SIDS suscepti-bility Pathways	(http://www.ingenuity.com)

				halothane, fluoxetine/ olanzapine, amobarbital, estazolam, atropine/ Hyoscyamine /phenobarbital/ scopolamine, clorazepate, acetaminophen/ butalbital, eszopiclone, quazepam, mephobarbital, hyoscyamine/ phenobarbital, amitriptyline/ chlordiazepoxide, acetaminophen/ butalbital/ caffeine/codeine, butabarbital, diazepam, temazepam, zolpidem, chlordiazepoxide, lorazepam, olanzapine, triazolam, flumazenil, clonazepam, flurazepam, midazolam, oxazepam, alprazolam, zaleplon, thiamylal, secobarbital, barbital, butalbital,		

				phenobarbital, pentobarbital, thiopental, propofol, ezogabine, acamprosate, desflurane, methoxyflurane, enflurane, topiramate, pregnenolone		
GABRA4	Gamma-Aminob-utyric acid type A receptor alpha4 subunit	Plasma Memb-rane	ion channel	methohexital, primidone, meprobamate, aspirin/ butalbital/ caffeine, aspirin/ meprobamate, aspirin/ butalbital/ caffeine/ codeine, hexobarbital, pagoclone, alphadolone, SEP 174559, heptabarbital, clobazam, nitrazepam, butobarbital, metyrapone/ oxazepam, acetaminophen/ butalbital/ caffeine, sevoflurane, isoflurane, gaboxadol, isoniazid, dexamethasone/	Its related pathways are Akt Signaling and GABA receptor activation	(http://www.ingenuity.com)

				olanzapine, felbamate, etomidate, muscimol, halothane, fluoxetine/ olanzapine, amobarbital, atropine/ hyoscyamine/ phenobarbital/ scopolamine, acetaminophen/ butalbital, eszopiclone, mephobarbital, hyoscyamine/ phenobarbital, acetaminophen/ butalbital/ caffeine/codeine, butabarbital, diazepam, temazepam, zolpidem, lorazepam, olanzapine, triazolam, clonazepam, flurazepam, midazolam, flunitrazepam, oxazepam, zaleplon, thiamylal, secobarbital, barbital,butalbital, phenobarbital, pentobarbital, thiopental, ezogabine,		

				desflurane, methoxyflurane, enflurane, pregnenolone		
GABRA5	Gamma-aminobutyric acid type A receptor alpha5 subunit	Plasma Membrane	ion channel	methohexital, primidone, meprobamate, aspirin/butalbital/caffeine,aspirin/meprobamate, aspirin/butalbital/caffeine/codeine, hexobarbital, pagoclone, alphadolone, SEP 174559, heptabarbital, zopiclone, clobazam, nitrazepam, adinazolam, butobarbital, metyrapone/oxazepam, acetaminophen/butalbital/caffeine, sevoflurane, isoflurane, gaboxadol, isoniazid, dexamethasone/olanzapine, felbamate, etomidate, muscimol, halothane, fluoxetine/olanzapine, amobarbital, estazolam, atropine/	Its related pathways are Akt Signaling and GABA receptor activation	(http://www.ingenuity.com)

				hyoscyamine/ phenobarbital/ scopolamine, clorazepate, acetaminophen/ butalbital, eszopiclone, quazepam, mephobarbital, hyoscyamine/ phenobarbital, amitriptyline/ chlordiazepoxide, acetaminophen/ butalbital/caffeine/ codeine, butabarbital, diazepam, temazepam, zolpidem, chlordiazepoxide, lorazepam, olanzapine, triazolam, flumazenil, clonazepam, flurazepam, midazolam, flunitrazepam, oxazepam, alprazolam, zaleplon, thiamylal, secobarbital, barbital,butalbital, phenobarbital, pentobarbital, thiopental, ezogabine, desflurane, methoxyflurane,		

				enflurane, pregnenolone		
GABRB2	Gamma-aminobutyric acid type A receptor beta2 subunit	Plasma Membrane	ion channel	methohexital, aspirin/ butalbital/ caffeine, aspirin/ butalbital/ caffeine/codeine, fospropofol, pagoclone, alphadolone, SEP 174559, nitrazepam, adinazolam, metyrapone/ oxazepam, acetaminophen/ butalbital/caffeine, sevoflurane, isoflurane, gaboxadol, isoniazid, dexamethasone/ olanzapine, felbamate, etomidate, muscimol, halothane, fluoxetine/ olanzapine, amobarbital, estazolam, atropine/ hyoscyamine/ phenobarbital/ scopolamine, acetaminophen/ butalbital, eszopiclone, mephobarbital,	Its related pathways are Akt Signaling and Neurophysiological process Glutamate regulation of Dopamine D1A receptor signaling	(http://www.ingenuity.com)

				hyoscyamine/ phenobarbital, acetaminophen/ butalbital/caffeine/ codeine, butabarbital, diazepam, temazepam, zolpidem, lorazepam, olanzapine, triazolam, clonazepam, flurazepam, midazolam, oxazepam, zaleplon, secobarbital, butalbital, phenobarbital, pentobarbital, thiopental, propofol, ezogabine, desflurane, methoxyflurane, enflurane, pregnenolone		
GAB RB3	Gamma-Aminobu -tyric acid type A receptor beta3 subunit	Plasma Memb-rane	ion channel	methohexital, aspirin/butalbital/ caffeine, aspirin/butalbital/ caffeine/codeine, fospropofol, pagoclone, alphadolone, SEP 174559, nitrazepam, adinazolam, piperazine, metyrapone/	Its related pathways are Akt Signaling and Neuroph-ysiological process Glutamate regulation of Dopamine D1A	(http://www.ingenuity.com)

				oxazepam, acetaminophen/ butalbital/caffeine, sevoflurane, isoflurane, gaboxadol, isoniazid, dexamethasone/ olanzapine, felbamate, etomidate, muscimol, halothane, fluoxetine/ olanzapine, amobarbital, estazolam, atropine/ hyoscyamine/ phenobarbital/ scopolamine, acetaminophen/ butalbital, eszopiclone, quazepam, mephobarbital, hyoscyamine/ phenobarbital, acetaminophen/ butalbital/caffeine/ codeine, butabarbital, diazepam, temazepam, zolpidem, lorazepam, olanzapine, triazolam, clonazepam, flurazepam, midazolam,	receptor signaling	

				oxazepam, zaleplon, secobarbital, butalbital, phenobarbital, pentobarbital, thiopental, propofol, ezogabine, desflurane, methoxyflurane, enflurane, pregnenolone		
GAB RG1	Gamma-aminobutyric acid type A receptor gamma1 subunit	Plasma Memb-rane	ion channel	pagoclone, alphadolone, SEP 174559, clobazam, nitrazepam, tracazolate, adinazolam, metyrapone/ oxazepam, sevoflurane, isoflurane, gaboxadol, isoniazid, dexamethasone/ olanzapine, felbamate, etomidate, muscimol, halothane, fluoxetine/ olanzapine, estazolam, clorazepate, eszopiclone, quazepam, amitriptyline/ chlordiazepoxide, diazepam,	Its related pathways are Akt Signaling and GABA ergic synapse	(http://www.ingenuity.com)

GABRG2	Gamma-Aminobu-tyric acid type A receptor gamma2 subunit	Plasma Memb-rane	ion channel	pagoclone, alphadolone, SEP 174559, clobazam, nitrazepam, tracazolate, adinazolam, metyrapone/ oxazepam, sevoflurane, isoflurane, gaboxadol, isoniazid, dexamethasone/ olanzapine, felbamate, etomidate, muscimol, halothane, fluoxetine/ olanzapine, estazolam, clorazepate, eszopiclone, quazepam, amitriptyline/ chlordiazepoxide, diazepam, temazepam, zolpidem, chlordiazepoxide, lorazepam, olanzapine, triazolam, flumazenil, clonazepam, flurazepam, midazolam, oxazepam, alprazolam, zaleplon,	Its related pathways are Akt Signaling and GABA ergic synapse	(http://www.ingenuity.com)

GABRG3	Gamma-Aminob-utyric acid type A receptor gamma3 subunit	Plasma Memb-rane	ion channel	pagoclone, alphadolone, SEP 174559, clobazam, nitrazepam, tracazolate, adinazolam, metyrapone/ oxazepam, sevoflurane, isoflurane, gaboxadol, isoniazid, dexamethasone/ olanzapine, felbamate, etomidate, muscimol, halothane, fluoxetine/ olanzapine, estazolam, eszopiclone, quazepam, diazepam, temazepam, zolpidem, lorazepam, olanzapine, triazolam, clonazepam, flurazepam, midazolam, oxazepam, zaleplon, secobarbital, phenobarbital, pentobarbital, ezogabine, desflurane, methoxyflurane,	Its related pathways are Akt Signaling and GABA ergic synapse	(http://www.ingenuity.com)

				enflurane, pregnenolone		
GABRP	Gamma-Aminobu-tyric acid type A receptor pi subunit	Plasma Memb-rane	ion channel	alphadolone, nitrazepam, adinazolam, metyrapone/ oxazepam, sevoflurane, isoflurane, isoniazid, dexamethasone/ olanzapine, felbamate, etomidate, halothane, fluoxetine/ olanzapine, estazolam, eszopiclone, quazepam, diazepam, temazepam, zolpidem, lorazepam, olanzapine, triazolam, flurazepam, midazolam, oxazepam, zaleplon, secobarbital, phenobarbital, pentobarbital, desflurane, methoxyflurane, enflurane	Alter the sensitivity of recombi-nant receptors to modulat-ory agents	(http://www.ingenuity.com)
IGHE	Immun-oglobulin heavy	Extra cellular Space	other	omalizumab	Trigger the	(http://ww

	constant epsilon				differen-tiation of B lympho-cytes into immuno-globulins-secreting plasma cells	w.ingenui ty. com)
IKBKG	Inhibitor of nuclear factor kappa B kinase subunit gamma	Nucleus	kinase		A mediator for TAX activation of NF-kappa-B	(Salt, Niemela et al., 2008) (http://www.ingenuity.com)
IL17F	Interleu-kin 17F	Extra cellular Space	cytokine	bimekizumab	Is expressed by activated T cells	(Gladiator, Wangler et al., 2013) (Trautwein-Weidner ,Gladiator et al., 2015)
IL17RA	Interleu-kin 17 receptor A	Plasma Memb-rane	Transm-embrane receptor	brodalumab	IL17A and its receptor play a pathogenic role in inflamm-atory and autoimm-une diseases	(Trautwein-Weidner ,Gladiator et al., 2015) (Cypowyj, Picard et al., 2012)

IL17RC	Interleukin 17 receptor C	Plasma Membrane	Transmembrane receptor	brodalumab	IL17A and its receptor play a pathogenic role in inflammation	(Eddens, Elsegeiny et al., 2017) (Veverka, Feldman et al., 2018)
IL18	Interleukin 18	Extra cellular Space	cytokine		Augment natural killer cell activity in spleen cells, stimulate IFNG production	(Ketelut-Carneiro, Silva et al., 2015) (Man, Karki et al., 2017)
IL23	Interleukin 23	Extra cellular Space	complex	ustekinumab, methotrexate/ ustekinumab, guselkumab	Mainly secreted by antigen presenting cells	(Angkasekwinai, Sringkarin et al., 2014) (Werner, Gessner et al., 2011)
KCNN4	Potassium calcium-activated channel subfamily N member 4	Plasma Membrane	ion channel	betamethasone/ clotrimazole, clotrimazole, senicapoc	part of the predominant calcium-activated potassium channel in T-lymphocytes	(http://www.ingenuity.com)
LSS	Lanosterol synthase	Cytoplasm	enzyme	oxiconazole	Its related pathways are Regulation of cholesterol biosynth-	(http://www.ingenuity.com)

					esis and Terpenoid backbone biosynth-esis	
MS4A 2	Membr-ane spanning 4-domains A2	Plasma Memb-rane	Transm-embrane receptor	omalizumab	Its related pathways are Immune response Fc epsilon RI pathway and Allograft rejection	(http://www.ingenuity.com)
MT-CYB	Mitoch-ondrial cytochr-ome B	Cytoplasm	enzyme	atovaquone, atovaquone/ proguanil	Its related pathways are Metabo-lism and Respirat-ory electron transport	(http://www.ingenuity.com)
NR3C1	Nuclear receptor subfamily 3 group C member 1	Nucleus	ligand-Depend-ent nuclear receptor	rimexolone, medrysone, clocortolone pivalate, diflorasone diacetate, fluorometholone, dexamethasone phosphate, cortisone acetate, halcinonide, flurandrenolide, desoximetasone, desonide, prednisolone, clobetasol	Involve in inflamm-atory responses, cellular prolifera-tion, and different-iation in target tissues	(http://www.ingenuity.com)

				propionate, fluocinolone acetonide, prednisone, hydrocortisone, triamcinolone, dexamethasone 21-acetate, betamethasone, dexamethasone, budesonide, fluticasone propionate, beclomethasone dipropionate, betamethasone acetate/ betametha-sone phosphate, triamcinolone acetonide, ciprofloxacin/ hydrocortisone, ciprofloxacin/ dexamethasone, ORG 34517, ciclesonide, betamethasone dipropionate/ calcipotriene, fluticasone furoate, budesonide/ formoterol, deacylcortivazol, difluprednate, formoterol/ Mometasone-furoate, beclomethasone, fluticasone		

				furoate/vilanterol, azelastine/fluticasone propionate, dexamethasone/lenalidomide/sorafenib, docetaxel/prednisone, carmustine/prednisone, cabazitaxel/prednisone, dexamethasone/lenalidomide, hydrocortisone/prednisone, dexamethasone/thalidomide, cyclophosphamide/prednisone/vincristine, hydrocortisone/mitoxantrone, mitoxantrone/prednisone, docetaxel/hydrocortisone, cytarabine/dexamethasone, dexamethasone/pomalidomide, bortezomib/dexamethasone, cyclophosphamide/dexamethasone/thalidomide, bortezomib/dexamethasone/doxorubicin, bortezomib/		

				dexamethasone/ lenalidomide,		
PLAU	Plasmin-ogen activator, urokinase	Extra cellular Space	peptidase		Its related pathways are Cell adhesion ECM remode-ling and NF-kappa B signaling pathway	(Sneideris, M. 1964) (http://www.ingenuity.com)
POLB	DNA polyme-rase beta	Nucleus	enzyme	nelarabine, cytarabine/ fludarabine phosphate, lamivudine/ nelfinavir/ stavudine, cytarabine/ Daunorubicin, lamivudine/ stavudine, lamivudine/ nevirapine/ stavudine, cladribine/ cytarabine/ daunorubicin, cytarabine/ daunorubicin/ tretinoin, cytarabine/ idarubicin, cytarabine/ etoposide, cytarabine/ etoposide/ mitoxantrone, cytarabine/	Its related pathways are Telomere C-strand Synthesis and HTLV-I infection	(http://www.ingenuity.com)

				dexamethasone, 5-azacytidine/ cytarabine/ decitabine, cytarabine/ mitoxantrone, cytarabine/ methotrexate, cladribine/ cytarabine/ filgrastim, clofarabine/ cyclophosphamide /etoposide, cytarabine/ filgrastim/ fludarabine phosphate, cladribine/ cytarabine/ filgrastim/ mitoxantrone, clofarabine/ cytarabine/ filgrastim, cytarabine/ filgrastim/ fludarabine phosphate/ idarubicin, cladribine/ cytarabine/ filgrastim/ idarubicin, cytarabine/ dexamethasone/ methotrexate, cladribine/ cytarabine/ filgrastim/ idarubicin/		

				plerixafor, cytarabine/ doxorubicin, clofarabine/ cytarabine/ filgrastim/ idarubicin, clofarabine/ filgrastim, cytarabine/ topotecan, cytarabine/ idarubicin/ sorafenib, cladribine/ cytarabine/ decitabine, cytarabine/ rituximab, clofarabine, cytarabine/ daunorubicin/ etoposide/ gemtuzumab ozogamicin, cytarabine/ recombinant interferon, arsenic trioxide/ cytarabine/ methotrexate, cytarabine/ idarubicin/ imatinib, cytarabine/ etoposide/ idarubicin, cytarabine/ daunorubicin/ etoposide		
POLG	DNA	Cytoplas	enzyme	lamivudine/	Its related	

	polymer-ase gamma, catalytic subunit	m		nelfinavir/ stavudine, lamivudine/ stavudine, lamivudine/ Nevirapine /stavudine, stavudine, vidarabine, zalcitabine	pathways are Cell Cycle Control of Chromo-somal Replicat-ion and Metabo-lism	(http://www.ingenuity.com)
PSME 3	Protea-some activator subunit 3	Cytoplas m	peptidas e		Its related pathways are Cell Cycle, Mitotic and RET signaling	(http://www.ingenuity.com)
PTX3	Pentraxin 3	Extra cellular Space	other		Involve in regulating inflammat -ion and comple-ment activation	(Bozza, Campo et al., 2014) (Marra, Sousa et al., 2014)
SLC6 A4	Solute carrier family 6 member 4	Plasma Memb-rane	Transpor -ter	acetaminophen/ clemastine/ pseudoephedrine, acetaminophen/ dexbrompheniramine/pseudoephedrine,dexbrompheniramine/pseudoephedrine,chlorpheniramine/ibuprofen/pseudoephedrine, cetirizine/pseudoephedrine, bicifadine, DOV-102, SLV-314, desvenlafaxine,	play a role in sudden infant death syndrome	(http://www.ingenuity.com)

				milnacipran, vortioxetine, phentermine/ topiramate, hydrocodone/ pseudoephedrine, levomilnacipran, amitriptyline/ ketamine, amitriptyline/ melatonin, fexofenadine/ pseudoephedrine, fenfluramine/ phentermine, acetaminophen/ tramadol, loratadine/ pseudoephedrine, methamphetamine, vilazodone, trazodone, sibutramine, ibuprofen/ pseudoephedrine, fluoxetine, imipramine, doxepin, sertraline, fluvoxamine, paroxetine, desipramine, clomipramine, fluoxetine/ olanzapine, amitriptyline/ perphenazine, amoxapine, amitriptyline/ chlordiazepoxide, naproxen/ pseudoephedrine,		

				(S)-duloxetine, fenfluramine, citalopram, phentermine, D-pseudoephe-drine, tramadol, trimipramine, nefazodone, venlafaxine, protriptyline, amitriptyline, nortriptyline, bupropion, N-methyl-3,4-met hylenedioxyamph-etamine, dexfenfluramine, escitalopram, mazindol, cocaine, desloratadine/pse-udoephedrine, carbinoxamine/ pseudoephedrine, pseudoephedrine/ triprolidine,		
SQLE	Squalene epoxidase	Cytoplas m	enzyme	tolnaftate, terbinafine, butenafine, naftifine	Its related pathways are Regulation of cholesterol biosynth-esis by SREBP and Terpenoid backbone biosynth-esis	(Bozza, Campo et al., 2014) (http://www.ingenuity.com)

TNFR SF1A	TNF receptor superfam -ily member 1A	Plasma Memb- rane	Transm- embrane receptor	GSK2862277	Play a role in cell survival, apoptosis, and inflamm- ation	(Deepe, Jr et al., 2012) (Pryhuber , Huyck et al., 2008)
TNFR SF1B	TNF receptor super- family member 1B	Plasma Memb- rane	Transm- embrane receptor		Mediate the recruit- ment of two anti- apoptotic proteins	(Deepe, Jr et al., 2012) (Pryhuber , Huyck et al., 2008)
ATP4 B	ATPase H+/K+ transport- ing beta subunit	Plasma Memb- rane	Transpor -ter	ilaprazole, tenatoprazole, AGN 201904, AR-H047108, dexlansoprazole, magnesium hydroxide/ omeprazole/ sodium bicarbonate, omeprazole/ sodium bicarbonate, esomeprazole/ naproxen, diclofenac/ omeprazole, clarithromycin/ omeprazole, esomeprazole magnesium, omeprazole, lansoprazole, rabeprazole, pantoprazole	Catalyze the hydrolysis of ATP coupled with the exchange of H(+) and K(+) ions	(http://www.ingenuity.com)
				flucytosine,		

TYMS	Thymidylate synthetase	Nucleus	enzyme	5-fluorouracil, tegafur, trimethoprim, cyclophosphamide/epirubicin, pralatrexate, tegafur/uracil, 5-fluorouracil/oxaliplatin, capecitabine/irinotecan, 5-fluorouracil/irinotecan, capecitabine/docetaxel, capecitabine/lapatinib, capecitabine/ixabepilone, bevacizumab/5-fluorouracil, bevacizumab/capecitabine/oxaliplatin, cyclophosphamide/docetaxel/epirubicin/5-fluorouracil/trastuzumab, capecitabine/oxaliplatin, bevacizumab/pemetrexed, capecitabine/trastuzumab, capecitabine/gemcitabine, capecitabine/docetaxel/gemcitabine, capecitabine/		

				epirubicin/ oxaliplatin, capecitabine/ erlotinib, capecitabine/ irinotecan/ oxaliplatin, bevacizumab/ capecitabine/ irinotecan/ oxaliplatin, bevacizumab/ capecitabine, capecitabine/ temozolomide, 5-fluorouracil/ irinotecan/ oxaliplatin, leucovorin/ methotrexate, 5-fluorouracil/ gemcitabine, 5-fluorouracil/ imiquimod, gimeracil/ Oxonic acid/ tegafur, gemcitabine/ pemetrexed, capecitabine/ thymidylate synthase inhibitor, DFP-11207, BGC945, capecitabine/ pertuzumab/ trastuzumab, raltitrexed, plevitrexed, nolatrexed, bevacizumab/		

References:

de Azevedo, M.I., Ferreiro, L., Da Silva, A.S., Tonin, A.A., Ruchel, J.B., Rezer, J.F., Franca, R.T., Zimmermann, C.E., Leal, D.B., Duarte, M.M., et al. (2014). E-NTPDase and E-ADA activities in rats experimental infected by Cryptococcus neoformans. Veterinary microbiology 174, 206-213.

Castro, V.S., Pimentel, V.C., Da Silva, A.S., Thome, G.R., Wolkmer, P., Castro, J.L., Costa, M.M., da Silva, C.B., Oliveira, D.C., Alves, S.H., et al. (2012). Adenosine deaminase activity in serum and lymphocytes of rats infected with Sporothrix schenckii. Mycopathologia 174, 31-39.

Cypowyj, S., Picard, C., Marodi, L., Casanova, J.L., and Puel, A. (2012). Immunity to infection in IL-17-deficient mice and humans. European journal of immunology 42, 2246-2254.

de Albuquerque, J.A.T., Banerjee, P.P., Castoldi, A., Ma, R., Zurro, N.B., Ynoue, L.H., Arslanian, C., Barbosa-Carvalho, M.U.W., Correia-Deur, J.E.M., Weiler, F.G., et al. (2018). The Role of AIRE in the Immunity Against Candida Albicans in a Model of Human Macrophages. Frontiers in immunology 9, 567.

Nanjappa, S.G., Hernandez-Santos, N., Galles, K., Wuthrich, M., Suresh, M., and Klein, B.S. (2015). Intrinsic MyD88-Akt1-mTOR Signaling Coordinates

Disparate Tc17 and Tc1 Responses during Vaccine Immunity against Fungal Pneumonia. PLoS pathogens 11, e1005161.

Moyes, D.L., Shen, C., Murciano, C., Runglall, M., Richardson, J.P., Arno, M., Aldecoa-Otalora, E., and Naglik, J.R. (2014). Protection against epithelial damage during Candida albicans infection is mediated by PI3K/Akt and mammalian target of rapamycin signaling. The Journal of infectious diseases 209, 1816-1826.

Wagner, R.D., Johnson, S.J., Danielsen, Z.Y., Lim, J.H., Mudalige, T., and Linder, S. (2017). Polyethylene glycol-functionalized poly (Lactic Acid-co-Glycolic Acid) and graphene oxide nanoparticles induce pro-inflammatory and apoptotic responses in Candida albicans-infected vaginal epithelial cells. PLoS One 12, e0175250.

Zhang, L., Huang, X., He, C., Zhang, Q.Y., Zou, X., Duan, K., and Gao, Q. (2018). Novel Fungal Pathogenicity and Leaf Defense Strategies Are Revealed by Simultaneous Transcriptome Analysis of Colletotrichum fructicola and Strawberry Infected by This Fungus. Frontiers in plant science 9, 434.

Li, N., Duan, Q., and Zhang, W. (2018). Risk factors and coping strategies of severe community-acquired pneumonia in chemotherapy induction period of acute leukemia. Oncology letters 15, 3566-3571.

Dou, Y.H., Du, J.K., Liu, H.L., and Shong, X.D. (2013). The role of

procalcitonin in the identification of invasive fungal infection-a systemic review and meta-analysis. Diagnostic microbiology and infectious disease 76, 464-469.

Ganesan, S., Rathinam, V.A.K., Bossaller, L., Army, K., Kaiser, W.J., Mocarski, E.S., Dillon, C.P., Green, D.R., Mayadas, T.N., Levitz, S.M., et al. (2014). Caspase-8 modulates dectin-1 and complement receptor 3-driven IL-1beta production in response to beta-glucans and the fungal pathogen, Candida albicans. Journal of immunology (Baltimore, Md : 1950) 193, 2519-2530.

Ketelut-Carneiro, N., Ghosh, S., Levitz, S.M., Fitzgerald, K.A., and da Silva, J.S. (2018). A Dectin-1-Caspase-8 Pathway Licenses Canonical Caspase-1 Inflammasome Activation and Interleukin-1beta Release in Response to a Pathogenic Fungus. The Journal of infectious diseases 217, 329-339.

Dreschers, S., Saupp, P., Hornef, M., Prehn, A., Platen, C., Morschhauser, J., and Orlikowsky, T.W. (2016). Reduced PICD in Monocytes Mounts Altered Neonate Immune Response to Candida albicans. PLoS One 11, e0166648.

Morais, E.A., Martins, E.M., Boelone, J.N., Gomes, D.A., and Goes, A.M. (2015). Immunization with recombinant Pb27 protein reduces the levels of pulmonary fibrosis caused by the inflammatory response against Paracoccidioides brasiliensis. Mycopathologia 179, 31-43.

Zhang, W., Dong, C., Zhang, Y., Zhu, J., Dai, H., and Bai, S. (2018). An

apple cyclic nucleotide-gated ion channel gene highly responsive to Botryosphaeria dothidea infection enhances the susceptibility of Nicotiana benthamiana to bacterial and fungal pathogens. Plant science : an international journal of experimental plant biology 269, 94-105.

Agarwal, P., Patel, K., and Agarwal, P.K. (2018). Ectopic Expression of JcWRKY Confers Enhanced Resistance in Transgenic Tobacco Against Macrophomina phaseolina. DNA and cell biology 37, 298-307.

Xiao, Y., Tang, J., Guo, H., Zhao, Y., Tang, R., Ouyang, S., Zeng, Q., Rappleye, C.A., Rajaram, M.V., Schlesinger, L.S., et al. (2016). Targeting CBLB as a potential therapeutic approach for disseminated candidiasis. Nature medicine 22, 906-914.

Wirnsberger, G., Zwolanek, F., Asaoka, T., Kozieradzki, I., Tortola, L., Wimmer, R.A., Kavirayani, A., Fresser, F., Baier, G., Langdon, W.Y., et al. (2016). Inhibition of CBLB protects from lethal Candida albicans sepsis. 22, 915-923.

Lofgren, S., Hullsiek, K.H., Morawski, B.M., Nabeta, H.W., Kiggundu, R., Taseera, K., Musubire, A., Schutz, C., Abassi, M., Bahr, N.C., et al. (2017). Differences in Immunologic Factors Among Patients Presenting with Altered Mental Status During Cryptococcal Meningitis. The Journal of infectious diseases 215, 693-697.

Overton, N.L., Simpson, A., Bowyer, P., and Denning, D.W. (2017). Genetic

susceptibility to severe asthma with fungal sensitization. International journal of immunogenetics 44, 93-106.

Hernandez-Santos, N., Wiesner, D.L., Fites, J.S., McDermott, A.J., Warner, T., Wuthrich, M., and Klein, B.S. (2018). Lung Epithelial Cells Coordinate Innate Lymphocytes and Immunity against Pulmonary Fungal Infection. Cell host & microbe 23, 511-522.e515.

Verma, A.H., Richardson, J.P., and Zhou, C. (2017). Oral epithelial cells orchestrate innate type 17 responses to Candida albicans through the virulence factor candidalysin. 2.

Arango, J.C., Puerta-Arias, J.D., Pino-Tamayo, P.A., Arboleda-Toro, D., and Gonzalez, A. (2017). Bone marrow-derived mesenchymal stem cells transplantation alters the course of experimental paracoccidioidomycosis by exacerbating the chronic pulmonary inflammatory response. Medical mycology.

Heung, L.J., and Hohl, T.M. (2016). DAP12 Inhibits Pulmonary Immune Responses to Cryptococcus neoformans. Infection and immunity 84, 1879-1886.

Hohl, T.M., Rivera, A., Lipuma, L., Gallegos, A., Shi, C., Mack, M., and Pamer, E.G. (2009). Inflammatory monocytes facilitate adaptive CD4 T cell responses during respiratory fungal infection. Cell host & microbe 6, 470-481.

Osterholzer, J.J., Chen, G.H., Olszewski, M.A., Curtis, J.L., Huffnagle, G.B., and Toews, G.B. (2009). Accumulation of CD11b+ lung dendritic cells in response to fungal infection results from the CCR2-mediated recruitment and differentiation of Ly-6Chigh monocytes. Journal of immunology (Baltimore, Md : 1950) 183, 8044-8053.

Alvarez, Y., Tuen, M., Shen, G., Nawaz, F., Arthos, J., Wolff, M.J., Poles, M.A., and Hioe, C.E. (2013). Preferential HIV infection of CCR6+ Th17 cells is associated with higher levels of virus receptor expression and lack of CCR5 ligands. Journal of virology 87, 10843-10854.

Scriven, J.E., Graham, L.M., Schutz, C., Scriba, T.J., Wilkinson, K.A., Wilkinson, R.J., Boulware, D.R., Urban, B.C., Meintjes, G., and Lalloo, D.G. (2017). The CSF Immune Response in HIV-1-Associated Cryptococcal Meningitis: Macrophage Activation, Correlates of Disease Severity, and Effect of Antiretroviral Therapy. Journal of acquired immune deficiency syndromes (1999) 75, 299-307.

Zawrotniak, M., Bochenska, O., Karkowska-Kuleta, J., Seweryn-Ozog, K., Aoki, W., Ueda, M., Kozik, A., and Rapala-Kozik, M. (2017). Aspartic Proteases and Major Cell Wall Components in Candida albicans Trigger the Release of Neutrophil Extracellular Traps. Frontiers in cellular and infection microbiology 7, 414.

Goyal, S., Castrillon-Betancur, J.C., Klaile, E., and Slevogt, H. (2018). The

Interaction of Human Pathogenic Fungi With C-Type Lectin Receptors. Frontiers in immunology 9, 1261.

Velasquez, L.N., Stuve, P., Gentilini, M.V., Swallow, M., Bartel, J., Lycke, N.Y., Barkan, D., Martina, M., Lujan, H.D., Kalay, H., et al. (2018). Targeting Mycobacterium tuberculosis Antigens to Dendritic Cells via the DC-Specific-ICAM3-Grabbing-Nonintegrin Receptor Induces Strong T-Helper 1 Immune Responses. Frontiers in immunology 9, 471.

Bernal, C.E., Zorro, M.M., Sierra, J., Gilchrist, K., Botero, J.H., Baena, A., and Ramirez-Pineda, J.R. (2016). Encephalitozoon intestinalis Inhibits Dendritic Cell Differentiation through an IL-6-Dependent Mechanism. Frontiers in cellular and infection microbiology 6, 4.

Evans, H.M., Simpson, A., Shen, S., Stromberg, A.J., Pickett, C.L., and Garvy, B.A. (2017). The Trophic Life Cycle Stage of the Opportunistic Fungal Pathogen Pneumocystis murina Hinders the Ability of Dendritic Cells To Stimulate CD4(+) T Cell Responses. Infection and immunity 85.

Fachin, A.L. Genes.

Leigh, J.E., McNulty, K.M., and Fidel, P.L., Jr. (2006). Characterization of the immune status of CD8+ T cells in oral lesions of human immunodeficiency virus-infected persons with oropharyngeal Candidiasis. Clinical and vaccine immunology : CVI 13, 678-683.

Spec, A., Shindo, Y., Burnham, C.A., Wilson, S., Ablordeppey, E.A., Beiter,

E.R., Chang, K., Drewry, A.M., and Hotchkiss, R.S. (2016). T cells from patients with Candida sepsis display a suppressive immunophenotype. Critical care 20, 15.

Li, Y., Fan, Y., Xia, B., Xiao, Q., Wang, Q., Sun, W., Zhang, H., and He, C. (2017). The immunosuppressive characteristics of FB1 by inhibition of maturation and function of BMDCs. International immunopharmacology 47, 206-211.

Zhu, L.L., Zhao, X.Q., Jiang, C., You, Y., Chen, X.P., Jiang, Y.Y., Jia, X.M., and Lin, X. (2013). C-type lectin receptors Dectin-3 and Dectin-2 form a heterodimeric pattern-recognition receptor for host defense against fungal infection. Immunity 39, 324-334.

Preite, N.W., Feriotti, C., Souza de Lima, D., da Silva, B.B., Condino-Neto, A., Pontillo, A., Calich, V.L.G., and Loures, F.V. (2018). The Syk-Coupled C-Type Lectin Receptors Dectin-2 and Dectin-3 Are Involved in Paracoccidioides brasiliensis Recognition by Human Plasmacytoid Dendritic Cells. Frontiers in immunology 9, 464.

Deng, H., You, Y., Liu, P., Zhao, H., Zhou, Y., Xie, Y., and Zuo, X. (2013). [Clinical study of invasive fungal infection secondary to systemic lupus erythematosus]. Zhong nan da xue xue bao Yi xue ban = Journal of Central South University Medical sciences 38, 182-185.

Meester, I., Rosas-Taraco, A.G., and Salinas-Carmona, M.C. (2013). Retnla

down-regulation and IL-13-rich environment correlate with inflammation severity in experimental actinomycetoma by Nocardia brasiliensis. Pathogens and disease 67, 214-220.

Mehra, T., Koberle, M., Braunsdorf, C., Mailander-Sanchez, D., Borelli, C., and Schaller, M. (2012). Alternative approaches to antifungal therapies. Experimental dermatology 21, 778-782.

Supasorn, O., Sringkarin, N., Srimanote, P., and Angkasekwinai, P. (2016). Matrix metalloproteinases contribute to the regulation of chemokine expression and pulmonary inflammation in Cryptococcus infection. Clinical and experimental immunology 183, 431-440.

Ren, X., Liu, W., and Liu, Y. (2018). Effects of fluconazole on the clinical outcome and immune response in fungal co-infected tuberculosis patients. Microbial pathogenesis 117, 148-152.

Fitzpatrick, M.E., Tedrow, J.R., Hillenbrand, M.E., Lucht, L., Richards, T., Norris, K.A., Zhang, Y., Sciurba, F.C., Kaminski, N., and Morris, A. (2014). Pneumocystis jirovecii colonization is associated with enhanced Th1 inflammatory gene expression in lungs of humans with chronic obstructive pulmonary disease. Microbiology and immunology 58, 202-211.

Yeh, C.C., Horng, H.C., Chou, H., Tai, H.Y., Shen, H.D., Hsieh, S.L., and Wang, P.H. (2017). Dectin-1-Mediated Pathway Contributes to Fusarium proliferatum-Induced CXCL-8 Release from Human Respiratory Epithelial

Cells. International journal of molecular sciences 18.

Klaile, E., Muller, M.M., Schafer, M.R., Clauder, A.K., Feer, S., Heyl, K.A., Stock, M., Klassert, T.E., Zipfel, P.F., Singer, B.B., et al. (2017). Binding of Candida albicans to Human CEACAM1 and CEACAM6 Modulates the Inflammatory Response of Intestinal Epithelial Cells. mBio 8.

Hamadeh, I.S., Klinker, K.P., Borgert, S.J., Richards, A.I., Li, W., Mangal, N., Hiemenz, J.W., Schmidt, S., Langaee, T.Y., Peloquin, C.A., et al. (2017). Impact of the CYP2C19 genotype on voriconazole exposure in adults with invasive fungal infections. Pharmacogenetics and genomics 27, 190-196.

Lamoureux, F., Duflot, T., Woillard, J.B., Metsu, D., Pereira, T., Compagnon, P., Morisse-Pradier, H., El Kholy, M., Thiberville, L., Stojanova, J., et al. (2016). Impact of CYP2C19 genetic polymorphisms on voriconazole dosing and exposure in adult patients with invasive fungal infections. International journal of antimicrobial agents 47, 124-131.

Hogan, D., and Wheeler, R.T. (2014). The complex roles of NADPH oxidases in fungal infection. Cellular microbiology 16, 1156-1167.

Sanna, M., Caocci, G., Ledda, A., Orru, F., Fozza, C., Deias, P., Tidore, G., Dore, F., and La Nasa, G. (2017). Glucose-6-phosphate dehydrogenase deficiency and risk of invasive fungal disease in patients with acute myeloid leukemia. Leukemia & lymphoma 58, 2558-2564.

Brzozowski, T., Zwolinska-Wcislo, M., Konturek, P.C., Kwiecien, S.,

Drozdowicz, D., Konturek, S.J., Stachura, J., Budak, A., Bogdal, J., Pawlik, W.W., et al. (2005). Influence of gastric colonization with Candida albicans on ulcer healing in rats: effect of ranitidine, aspirin and probiotic therapy. Scandinavian journal of gastroenterology 40, 286-296.

Arasu, A., Kumaresan, V., Sathyamoorthi, A., Arasu, M.V., Al-Dhabi, N.A., and Arockiaraj, J. (2016). Coagulation profile, gene expression and bioinformatics characterization of coagulation factor X of striped murrel Channa striatus. Fish & shellfish immunology 55, 149-158.

Wagener, J., Schneider, J.J., Baxmann, S., Kalbacher, H., Borelli, C., Nuding, S., Kuchler, R., Wehkamp, J., Kaeser, M.D., Mailander-Sanchez, D., et al. (2013). A peptide derived from the highly conserved protein GAPDH is involved in tissue protection by different antifungal strategies and epithelial immunomodulation. The Journal of investigative dermatology 133, 144-153.

Chidambaram, J.D., Kannambath, S., Srikanthi, P., Shah, M., Lalitha, P., Elakkiya, S., Bauer, J., Prajna, N.V., Holland, M.J., and Burton, M.J. (2017). Persistence of Innate Immune Pathways in Late Stage Human Bacterial and Fungal Keratitis: Results from a Comparative Transcriptome Analysis. Frontiers in cellular and infection microbiology 7, 193.

Fecher, R.A., Horwath, M.C., and Friedrich, D. (2016). Inverse Correlation between IL-10 and HIF-1alpha in Macrophages Infected with Histoplasma capsulatum. 197, 565-579.

Toth, E.J., Boros, E., Hoffmann, A., Szebenyi, C., Homa, M., Nagy, G., Vagvolgyi, C., Nagy, I., and Papp, T. (2017). Interaction of THP-1 Monocytes with Conidia and Hyphae of Different Curvularia Strains. Frontiers in immunology 8, 1369.

Elsegeiny, W., Marr, K.A., and Williamson, P.R. (2018). Immunology of Cryptococcal Infections: Developing a Rational Approach to Patient Therapy. Frontiers in immunology 9, 651.

Koehm, S., Slavin, R.G., Hutcheson, P.S., Trejo, T., David, C.S., and Bellone, C.J. (2007). HLA-DRB1 alleles control allergic bronchopulmonary aspergillosis-like pulmonary responses in humanized transgenic mice. The Journal of allergy and clinical immunology 120, 570-577.

Nilsson, O.G., Lindgren, A., Brandt, L., and Saveland, H. (2002). Prediction of death in patients with primary intracerebral hemorrhage: a prospective study of a defined population. Journal of neurosurgery 97, 531-536.

Pandit, H., Madhukaran, S.P., Nayak, A., and Madan, T. (2012). SP-A and SP-D in host defense against fungal infections and allergies. Frontiers in bioscience (Elite edition) 4, 651-661.

Geunes-Boyer, S., Beers, M.F., Perfect, J.R., Heitman, J., and Wright, J.R. (2012). Surfactant protein D facilitates Cryptococcus neoformans infection. Infection and immunity 80, 2444-2453.

Goodrich, Julia K., Di Rienzi, Sara C., Poole, Angela C., Koren, O., Walters,

William A., Caporaso, J.G., Knight, R., and Ley, Ruth E. (2014). Conducting a Microbiome Study. Cell 158, 250-262.

Zhang, M., Sun, D., Liu, G., Wu, H., Zhou, H., and Shi, M. (2016). Real-time in vivo imaging reveals the ability of neutrophils to remove Cryptococcus neoformans directly from the brain vasculature. Journal of leukocyte biology 99, 467-473.

Quaresma, J.A., Brito, M.V., Sousa, J.R., Silva, L.M., Hirai, K.E., Araujo, R.S., de Brito, A.C., Carneiro, F.R., Fuzii, H.T., Pagliari, C., et al. (2015). Analysis of microvasculature phenotype and endothelial activation markers in skin lesions of lacaziosis (Lobomycosis). Microbial pathogenesis 78, 29-36.

de Araujo, E.F., Feriotti, C., Galdino, N.A.L., Preite, N.W., Calich, V.L.G., and Loures, F.V. (2017). The IDO-AhR Axis Controls Th17/Treg Immunity in a Pulmonary Model of Fungal Infection. Frontiers in immunology 8, 880.

Zelante, T., Pieraccini, G., Scaringi, L., Aversa, F., and Romani, L. (2016). Learning from other diseases: protection and pathology in chronic fungal infections. Seminars in immunopathology 38, 239-248.

del Fresno, C., Soulat, D., Roth, S., Blazek, K., Udalova, I., Sancho, D., Ruland, J., and Ardavin, C. (2013). Interferon-beta production via Dectin-1-Syk-IRF5 signaling in dendritic cells is crucial for immunity to C. albicans. Immunity 38, 1176-1186.

Smith, N.L., Hankinson, J., Simpson, A., Denning, D.W., and Bowyer, P. (2014). Reduced expression of TLR3, TLR10 and TREM1 by human macrophages in Chronic cavitary pulmonary aspergillosis, and novel associations of VEGFA, DENND1B and PLAT. Clinical microbiology and infection : the official publication of the European Society of Clinical Microbiology and Infectious Diseases 20, O960-968.

Lilic, D. (2012). Unravelling fungal immunity through primary immune deficiencies. Current opinion in microbiology 15, 420-426.

Huppler, A.R., Bishu, S., and Gaffen, S.L. (2012). Mucocutaneous candidiasis: the IL-17 pathway and implications for targeted immunotherapy. Arthritis research & therapy 14, 217.

Piehler, D., Eschke, M., Schulze, B., Protschka, M., Muller, U., Grahnert, A., Richter, T., Heyen, L., Kohler, G., Brombacher, F., et al. (2016). The IL-33 receptor (ST2) regulates early IL-13 production in fungus-induced allergic airway inflammation. Mucosal immunology 9, 937-949.

Dhalla, F., Fox, H., Davenport, E.E., Sadler, R., Anzilotti, C., van Schouwenburg, P.A., Ferry, B., Chapel, H., Knight, J.C., and Patel, S.Y. (2016). Chronic mucocutaneous candidiasis: characterization of a family with STAT-1 gain-of-function and development of an ex-vivo assay for Th17 deficiency of diagnostic utility. Clinical and experimental immunology 184, 216-227.

Smith, N.L., Hankinson, J., Simpson, A., Bowyer, P., and Denning, D.W. (2014). A prominent role for the IL1 pathway and IL15 in susceptibility to chronic cavitary pulmonary aspergillosis. Clinical microbiology and infection : the official publication of the European Society of Clinical Microbiology and Infectious Diseases 20, O480-488.

Ahlgren, K.M., Moretti, S., Lundgren, B.A., Karlsson, I., Ahlin, E., Norling, A., Hallgren, A., Perheentupa, J., Gustafsson, J., Rorsman, F., et al. (2011). Increased IL-17A secretion in response to Candida albicans in autoimmune polyendocrine syndrome type 1 and its animal model. European journal of immunology 41, 235-245.

Garth, J.M., Reeder, K.M., Godwin, M.S., Mackel, J.J., Dunaway, C.W., Blackburn, J.P., and Steele, C. (2017). IL-33 Signaling Regulates Innate IL-17A and IL-22 Production via Suppression of Prostaglandin E2 during Lung Fungal Infection. Journal of immunology (Baltimore, Md : 1950) 199, 2140-2148.

Zheng, J., van de Veerdonk, F.L., Crossland, K.L., Smeekens, S.P., Chan, C.M., Al Shehri, T., Abinun, M., Gennery, A.R., Mann, J., Lendrem, D.W., et al. (2015). Gain-of-function STAT1 mutations impair STAT3 activity in patients with chronic mucocutaneous candidiasis (CMC). European journal of immunology 45, 2834-2846.

Patin, E.C., Jones, A.V., and Thompson, A. (2016). IL-27 Induced by Select

Candida spp. via TLR7/NOD2 Signaling and IFN-beta Production Inhibits Fungal Clearance. 197, 208-221.

Depner, M., Fuchs, S., Raabe, J., Frede, N., Glocker, C., Doffinger, R., Gkrania-Klotsas, E., Kumararatne, D., Atkinson, T.P., Schroeder, H.W., Jr., et al. (2016). The Extended Clinical Phenotype of 26 Patients with Chronic Mucocutaneous Candidiasis due to Gain-of-Function Mutations in STAT1. Journal of clinical immunology 36, 73-84.

Wanachiwanawin, W., Mendoza, L., Visuthisakchai, S., Mutsikapan, P., Sathapatayavongs, B., Chaiprasert, A., Suwanagool, P., Manuskiatti, W., Ruangsetakit, C., and Ajello, L. (2004). Efficacy of immunotherapy using antigens of Pythium insidiosum in the treatment of vascular pythiosis in humans. Vaccine 22, 3613-3621.

Radovanovic, I., Leung, V., Iliescu, A., Bongfen, S.E., Mullick, A., Langlais, D., and Gros, P. (2014). Genetic control of susceptibility to Candida albicans in SM/J mice. Journal of immunology (Baltimore, Md : 1950) 193, 1290-1300.

Wevers, B.A., Kaptein, T.M., Zijlstra-Willems, E.M., Theelen, B., Boekhout, T., Geijtenbeek, T.B., and Gringhuis, S.I. (2014). Fungal engagement of the C-type lectin mincle suppresses dectin-1-induced antifungal immunity. Cell host & microbe 15, 494-505.

Chen, R., Ji, G., Wang, L., Ren, H., and Xi, L. (2016). Activation of ERK1/2

and TNF-alpha production are regulated by calcium/calmodulin signaling pathway during Penicillium marneffei infection within human macrophages. Microbial pathogenesis 93, 95-99.

Perez-Nadales, E., and Di Pietro, A. (2011). The membrane mucin Msb2 regulates invasive growth and plant infection in Fusarium oxysporum. The Plant cell 23, 1171-1185.

Higuchi, Y. (2015). Initial fungal effector production is mediated by early endosome motility. Communicative & integrative biology 8, e1025187.

Bielska, E., Higuchi, Y., Schuster, M., Steinberg, N., Kilaru, S., Talbot, N.J., and Steinberg, G. (2014). Long-distance endosome trafficking drives fungal effector production during plant infection. Nature communications 5, 5097.

Selander, C., Engblom, C., Nilsson, G., Scheynius, A., and Andersson, C.L. (2009). TLR2/MyD88-dependent and -independent activation of mast cell IgE responses by the skin commensal yeast Malassezia sympodialis. Journal of immunology (Baltimore, Md : 1950) 182, 4208-4216.

Carpino, N., Naseem, S., Frank, D.M., and Konopka, J.B. (2017). Modulating Host Signaling Pathways to Promote Resistance to Infection by Candida albicans. Frontiers in cellular and infection microbiology 7, 481.

Zhao, G., Xu, Q., Lin, J., Chen, W., Cui, T., Hu, L., and Jiang, N. (2017). The role of Mincle in innate immune to fungal keratitis. Journal of infection in developing countries 11, 89-97.

Osthoff, M., Wojtowicz, A., Tissot, F., Jorgensen, C., Thiel, S., Zimmerli, S., Marchetti, O., Khanna, N., Bochud, P.Y., and Trendelenburg, M. (2016). Association of lectin pathway proteins with intra-abdominal Candida infection in high-risk surgical intensive-care unit patients. A prospective cohort study within the fungal infection network of Switzerland. The Journal of infection 72, 377-385.

Frakking, F.N., Israels, J., Kremer, L.C., Kuijpers, T.W., Caron, H.N., and van de Wetering, M.D. (2011). Mannose-binding lectin (MBL) and the risk for febrile neutropenia and infection in pediatric oncology patients with chemotherapy. Pediatric blood & cancer 57, 89-96.

Hu, J., Hu, Y., Chen, S., Dong, C., Zhang, J., Li, Y., Yang, J., Han, X., Zhu, X., and Xu, G. (2014). Role of activated macrophages in experimental Fusarium solani keratitis. Experimental eye research 129, 57-65.

Mirkov, I., Belij, S., Kataranovski, M., Zolotarevski, L., Glamoclija, J., Stojanovic, I., and Stosic-Grujicic, S. (2012). The relevance of the migration inhibitory factor (MIF) for peripheral tissue response in murine sublethal systemic Aspergillus fumigatus infection. Medical mycology 50, 476-487.

Hajari Taheri, F., Seyedolmohadesin, M., Bayat, M., Mahdavi, M., Yazdi, M.H., Eslamifar, A., and Abolhasani, M. (2013). The effect of Candida albicans systemic infection on matrix metalloproteinases in breast cancer bearing BALB/c mice. Iranian journal of allergy, asthma, and immunology

12, 81-85.

Zhan, Y., Zhang, H., Liu, R., Wang, W., Qi, J., and Zhang, Y. (2016). Eupolyphaga sinensis Walker Ethanol Extract Suppresses Cell Growth and Invasion in Human Breast Cancer Cells. Integrative cancer therapies 15, 102-112.

Taylor, P.R., Roy, S., Meszaros, E.C., Sun, Y., Howell, S.J., Malemud, C.J., and Pearlman, E. (2016). JAK/STAT regulation of Aspergillus fumigatus corneal infections and IL-6/23-stimulated neutrophil, IL-17, elastase, and MMP9 activity. Journal of leukocyte biology 100, 213-222.

Cui, N., Wang, H., Su, L.X., Zhang, J.H., Long, Y., and Liu, D.W. (2017). Role of Triggering Receptor Expressed on Myeloid Cell-1 Expression in Mammalian Target of Rapamycin Modulation of CD8(+) T-cell Differentiation during the Immune Response to Invasive Pulmonary Aspergillosis. Chinese medical journal 130, 1211-1217.

Intini, G., Aguirre, A., and Bobek, L.A. (2003). Efficacy of human salivary mucin MUC7-derived peptide and histatin 5 in a murine model of candidiasis. International journal of antimicrobial agents 22, 594-600.

Satyanarayana, J., Situ, H., Narasimhamurthy, S., Bhayani, N., Bobek, L.A., and Levine, M.J. (2000). Divergent solid-phase synthesis and candidacidal activity of MUC7 D1, a 51-residue histidine-rich N-terminal domain of human salivary mucin MUC7. The journal of peptide research : official

journal of the American Peptide Society 56, 275-282.
Araujo, V.C., Demasi, A.P., Soares, A.B., Passador-Santos, F., Napimoga, M.H., Martinez, E.F., Freitas, N.S., and Araujo, N.S. (2013). Neutrophils in oral paracoccidioidomycosis and the involvement of Nrf2. PLoS One 8, e76976.
Ishida, Y., Ohta, K., Naruse, T., Kato, H., Fukui, A., Shigeishi, H., Nishi, H., Tobiume, K., and Takechi, M. (2018). Candida albicans beta-Glucan -Containing Particles Increase HO-1 Expression in Oral Keratinocytes via a Reactive Oxygen Species/p38 Mitogen-Activated Protein Kinase/Nrf2 Pathway. Infection and immunity 86.
Plato, A., Hardison, S.E., and Brown, G.D. (2015). Pattern recognition receptors in antifungal immunity. Seminars in immunopathology 37, 97-106.
Malireddi, R.K., and Kanneganti, T.D. (2013). Role of type I interferons in inflammasome activation, cell death, and disease during microbial infection. Frontiers in cellular and infection microbiology 3, 77.
Morgado, F.N., Schubach, A.O., Barros, M.B., and Conceicao-Silva, F. (2011). The in situ inflammatory profile of lymphocutaneous and fixed forms of human sporotrichosis. Medical mycology 49, 612-620.
Sellathamby, S., Lakshmi, K.M., Busson, M., Viswabandya, A., George, B., Mathews, V., Chandy, M., Charron, D., Krishnamoorthy, R., Tamouza, R., et al. (2012). Polymorphisms in the immunoregulatory genes are associated

with hematopoietic recovery and increased susceptibility to bacterial infections in patients with thalassaemia major undergoing matched related hematopoietic stem cell transplantation. Biology of blood and marrow transplantation : journal of the American Society for Blood and Marrow Transplantation 18, 1219-1226.

Liu, Y., Shetty, A.C., Schwartz, J.A., Bradford, L.L., Xu, W., Phan, Q.T., Kumari, P., Mahurkar, A., Mitchell, A.P., Ravel, J., et al. (2015). New signaling pathways govern the host response to C. albicans infection in various niches. Genome research 25, 679-689.

Hua, X., Chi, W., Su, L., Li, J., Zhang, Z., and Yuan, X. (2017). ROS-induced Oxidative Injury involved in Pathogenesis of Fungal Keratitis via p38 MAPK Activation. Sci Rep 7, 10421.

Wang, M., Wang, S., Wang, W., Wang, Y., Wang, H., and Zhu, W. (2017). Inhibition effects of novel polyketide compound PPQ-B against influenza A virus replication by interfering with the cellular EGFR pathway. Antiviral research 143, 74-84.

Petrucelli, M.F., Peronni, K., and Sanches, P.R. (2018). Dual RNA-Seq Analysis of Trichophyton rubrum and HaCat Keratinocyte Co-Culture Highlights Important Genes for Fungal-Host Interaction. 9.

Saxena, S., Madan, T., Shah, A., Muralidhar, K., and Sarma, P.U. (2003). Association of polymorphisms in the collagen region of SP-A2 with

increased levels of total IgE antibodies and eosinophilia in patients with allergic bronchopulmonary aspergillosis. The Journal of allergy and clinical immunology 111, 1001-1007.

Brunke, S., Quintin, J., Kasper, L., Jacobsen, I.D., Richter, M.E., Hiller, E., Schwarzmuller, T., d'Enfert, C., Kuchler, K., Rupp, S., et al. (2015). Of mice, flies--and men? Comparing fungal infection models for large-scale screening efforts. Disease models & mechanisms 8, 473-486.

Yamauchi, J., Takayanagi, N., Komeda, K., Takano, Y., and Okuno, T. (2004). cAMP-pKA signaling regulates multiple steps of fungal infection cooperatively with Cmk1 MAP kinase in Colletotrichum lagenarium. Molecular plant-microbe interactions : MPMI 17, 1355-1365.

Shi, D., Li, D., Wang, Q., Kong, X., Mei, H., Shen, Y., and Liu, W. (2018). Silencing SOCS1 in dendritic cells promote survival of mice with systemic Candida albicans infection via inducing Th1-cell differentiation. Immunology letters 197, 53-62.

Shi, D., Li, D., Yin, Q., Qiu, Y., Yan, H., Shen, Y., Lu, G., and Liu, W. (2015). Silenced suppressor of cytokine signaling 1 (SOCS1) enhances the maturation and antifungal immunity of dendritic cells in response to Candida albicans in vitro. Immunologic research 61, 206-218.

Doherty, T.A., Khorram, N., Chang, J.E., Kim, H.K., Rosenthal, P., Croft, M., and Broide, D.H. (2012). STAT6 regulates natural helper cell proliferation

during lung inflammation initiated by Alternaria. American journal of physiology Lung cellular and molecular physiology 303, L577-588.

Bloodworth, M.H., Newcomb, D.C., Dulek, D.E., Stier, M.T., Cephus, J.Y., Zhang, J., Goleniewska, K., Kolls, J.K., and Peebles, R.S., Jr. (2016). STAT6 Signaling Attenuates Interleukin-17-Producing gammadelta T Cells during Acute Klebsiella pneumoniae Infection. Infection and immunity 84, 1548-1555.

Rostan, O., Arshad, M.I., Piquet-Pellorce, C., Robert-Gangneux, F., Gangneux, J.P., and Samson, M. (2015). Crucial and diverse role of the interleukin-33/ST2 axis in infectious diseases. Infection and immunity 83, 1738-1748.

Mao, L., Zhang, L., Li, H., Chen, W., Wang, H., Wu, S., Guo, C., Lu, A., Yang, G., An, L., et al. (2014). Pathogenic fungus Microsporum canis activates the NLRP3 inflammasome. Infection and immunity 82, 882-892.

Kottom, T.J., Hebrink, D.M., Jenson, P.E., Marsolek, P.L., Wuthrich, M., Wang, H., Klein, B., Yamasaki, S., and Limper, A.H. (2018). Dectin-2 Is a C-Type Lectin Receptor that Recognizes Pneumocystis and Participates in Innate Immune Responses. American journal of respiratory cell and molecular biology 58, 232-240.

Sawada, Y., Nakamura, M., Kabashima-Kubo, R., Shimauchi, T., Kobayashi, M., and Tokura, Y. (2012). Defective epidermal innate immunity and

resultant superficial dermatophytosis in adult T-cell leukemia/lymphoma. Clinical cancer research : an official journal of the American Association for Cancer Research 18, 3772-3779.

Gorjestani, S., Darnay, B.G., and Lin, X. (2012). Tumor necrosis factor receptor-associated factor 6 (TRAF6) and TGFbeta-activated kinase 1 (TAK1) play essential roles in the C-type lectin receptor signaling in response to Candida albicans infection. The Journal of biological chemistry 287, 44143-44150.

Lupianez, C.B., Canet, L.M., Carvalho, A., Alcazar-Fuoli, L., Springer, J., Lackner, M., Segura-Catena, J., Comino, A., Olmedo, C., Rios, R., et al. (2015). Polymorphisms in Host Immunity-Modulating Genes and Risk of Invasive Aspergillosis: Results from the AspBIOmics Consortium. Infection and immunity 84, 643-657.

Qureshi, M.H., Cook-Mills, J., Doherty, D.E., and Garvy, B.A. (2003). TNF-alpha-dependent ICAM-1- and VCAM-1-mediated inflammatory responses are delayed in neonatal mice infected with Pneumocystis carinii. Journal of immunology (Baltimore, Md : 1950) 171, 4700-4707.

Liu, Y., Yang, K., Qin, Q., Lin, G., Hu, T., Xu, Z., and Wang, S. (2018). G Protein alpha Subunit GpaB is Required for Asexual Development, Aflatoxin Biosynthesis and Pathogenicity by Regulating cAMP Signaling in Aspergillus flavus. Toxins 10.

Zhao, X., Guo, Y., and Jiang, C. (2017). JNK1 negatively controls antifungal innate immunity by suppressing CD23 expression. 23, 337-346.

Arnold-Schrauf, C., Berod, L., and Sparwasser, T. (2015). Dendritic cell specific targeting of MyD88 signalling pathways in vivo. European journal of immunology 45, 32-39.

Ishii, K., and Kawakami, K. (2014). [Up-to-date findings in the host defence mechanism to cryptococcus infection]. Medical mycology journal 55, J107-114.

Nakamura, Y., Sato, K., Yamamoto, H., Matsumura, K., Matsumoto, I., Nomura, T., Miyasaka, T., Ishii, K., Kanno, E., Tachi, M., et al. (2015). Dectin-2 deficiency promotes Th2 response and mucin production in the lungs after pulmonary infection with Cryptococcus neoformans. Infection and immunity 83, 671-681.

Bernal, C.E., Zorro, M.M., Sierra, J., Gilchrist, K., Botero, J.H., Baena, A., and Ramirez-Pineda, J.R. (2016). Encephalitozoon intestinalis Inhibits Dendritic Cell Differentiation through an IL-6-Dependent Mechanism. Frontiers in cellular and infection microbiology 6, 4.

Li, X., Kang, Y.Q., Luo, Y.L., Zhang, K.Q., Zou, C.G., and Liang, L.M. (2017). The NADPH oxidase AoNoxA in Arthrobotrys oligospora functions as an initial factor in the infection of Caenorhabditis elegans. Journal of microbiology (Seoul, Korea) 55, 885-891.

Schulze, B., Piehler, D., Eschke, M., Heyen, L., Protschka, M., Kohler, G., and Alber, G. (2016). Therapeutic expansion of CD4+FoxP3+ regulatory T cells limits allergic airway inflammation during pulmonary fungal infection. Pathogens and disease 74, ftw020.

Michelsen, M.M., Mygind, N.D., Pena, A., Olsen, R.H., Christensen, T.E., Ghotbi, A.A., Hasbak, P., Kjaer, A., Gustafsson, I., Hansen, P.R., et al. (2017). Transthoracic Doppler echocardiography compared with positron emission tomography for assessment of coronary microvascular dysfunction: The iPOWER study. International journal of cardiology 228, 435-443.

Maruyama, K., Fukasaka, M., Vandenbon, A., Saitoh, T., Kawasaki, T., Kondo, T., Yokoyama, K.K., Kidoya, H., Takakura, N., Standley, D., et al. (2012). The transcription factor Jdp2 controls bone homeostasis and antibacterial immunity by regulating osteoclast and neutrophil differentiation. Immunity 37, 1024-1036.

Maruyama, K., Takayama, Y., Kondo, T., Ishibashi, K.I., Sahoo, B.R., Kanemaru, H., Kumagai, Y., Martino, M.M., Tanaka, H., Ohno, N., et al. (2017). Nociceptors Boost the Resolution of Fungal Osteoinflammation via the TRP Channel-CGRP-Jdp2 Axis. Cell reports 19, 2730-2742.

Dong, C., Gao, N., Ross, B.X., and Yu, F.X. (2017). ISG15 in Host Defense Against Candida albicans Infection in a Mouse Model of Fungal Keratitis. Investigative ophthalmology & visual science 58, 2948-2958.

Wang, H., and Li, M. (2016). MyD88 Shapes Vaccine Immunity by Extrinsically Regulating Survival of CD4+ T Cells during the Contraction Phase. 12, e1005787.

Bao, W., Jin, L., Fu, H.J., Shen, Y.N., Lu, G.X., Mei, H., Cao, X.Z., Wang, H.S., and Liu, W.D. (2013). Interleukin-22 mediates early host defense against Rhizomucor pusilluscan pathogens. PLoS One 8, e65065.

Havel, V.E., Wool, N.K., Ayad, D., Downey, K.M., Wilson, C.F., Larsen, P., Djordjevic, J.T., and Panepinto, J.C. (2011). Ccr4 promotes resolution of the endoplasmic reticulum stress response during host temperature adaptation in Cryptococcus neoformans. Eukaryotic cell 10, 895-901.

Zhou, H., Shen, T., Luo, Y., Liu, L., Chen, W., Xu, B., Han, X., Pang, J., Rivera, C.A., and Huang, S. (2010). The antitumor activity of the fungicide ciclopirox. International journal of cancer 127, 2467-2477.

Mane, A., Gujar, P., Chandra, J., Lokhande, R., Dhamgaye, T., Ghorpade, S., and Risbud, A. (2015). Pneumocystis jirovecii infection and the associated dihydropteroate synthase (DHPS) and dihydrofolate reductase (DHFR) mutations in HIV-positive individuals from Pune, India. Mycopathologia 179, 141-145.

Munoz, C., Zuluaga, A., Restrepo, A., Tobon, A., Cano, L.E., and Gonzalez, A. (2012). Molecular diagnosis and detection of Pneumocystis jirovecii DHPS and DHFR genotypes in respiratory specimens from Colombian

patients. Diagnostic microbiology and infectious disease 72, 204-213.

Casadevall, A., and Pirofski, L.A. (2012). Immunoglobulins in defense, pathogenesis, and therapy of fungal diseases. Cell host & microbe 11, 447-456.

Ribeiro, A.M., Souza, A.C., Amaral, A.C., Vasconcelos, N.M., Jeronimo, M.S., Carneiro, F.P., Faccioli, L.H., Felipe, M.S., Silva, C.L., and Bocca, A.L. (2013). Nanobiotechnological approaches to delivery of DNA vaccine against fungal infection. Journal of biomedical nanotechnology 9, 221-230.

Chang, Y.Y., van der Velden, J., van der Wier, G., Kramer, D., Diercks, G.F., van Geel, M., Coenraads, P.J., Zeeuwen, P.L., and Jonkman, M.F. (2012). Keratolysis exfoliativa (dyshidrosis lamellosa sicca): a distinct peeling entity. The British journal of dermatology 167, 1076-1084.

Knorr, E., Schmidtberg, H., Vilcinskas, A., and Altincicek, B. (2009). MMPs regulate both development and immunity in the tribolium model insect. PLoS One 4, e4751.

Conti, H.R., Whibley, N., Coleman, B.M., Garg, A.V., Jaycox, J.R., and Gaffen, S.L. (2015). Signaling through IL-17C/IL-17RE is dispensable for immunity to systemic, oral and cutaneous candidiasis. PLoS One 10, e0122807.

Dejima, T., Shibata, K., Yamada, H., Hara, H., Iwakura, Y., Naito, S., and Yoshikai, Y. (2011). Protective role of naturally occurring interleukin-17A-

producing gammadelta T cells in the lung at the early stage of systemic candidiasis in mice. Infection and immunity 79, 4503-4510.

Higurashi, H., Arai, M., Watanabe, A., Igari, H., Seki, N., Kamei, K., and Kuriyama, T. (2007). Gene expression profiling of polymorphonuclear leukocytes treated with the culture filtrate of Aspergillus fumigatus and gliotoxin. Microbiology and immunology 51, 407-419.

Conti, H.R., Whibley, N., Coleman, B.M., Garg, A.V., Jaycox, J.R., and Gaffen, S.L. (2015). Signaling through IL-17C/IL-17RE is dispensable for immunity to systemic, oral and cutaneous candidiasis. PLoS One 10, e0122807.

Milner, J.D., Sandler, N.G., and Douek, D.C. (2010). Th17 cells, Job's syndrome and HIV: opportunities for bacterial and fungal infections. Current opinion in HIV and AIDS 5, 179-183.

Carpenter, K.J., Ewing, J.L., Schuh, J.M., Ness, T.L., Kunkel, S.L., Aparici, M., Miralpeix, M., and Hogaboam, C.M. (2005). Therapeutic targeting of CCR1 attenuates established chronic fungal asthma in mice. British journal of pharmacology 145, 1160-1172.

Lionakis, M.S., Fischer, B.G., Lim, J.K., Swamydas, M., Wan, W., Richard Lee, C.C., Cohen, J.I., Scheinberg, P., Gao, J.L., and Murphy, P.M. (2012). Chemokine receptor Ccr1 drives neutrophil-mediated kidney immuno-pathology and mortality in invasive candidiasis. PLoS pathogens 8,

e1002865.

Kroetz, D.N., and Deepe, G.S., Jr. (2010). CCR5 dictates the equilibrium of proinflammatory IL-17+ and regulatory Foxp3+ T cells in fungal infection. Journal of immunology (Baltimore, Md : 1950) 184, 5224-5231.

Kroetz, D.N., and Deepe, G.S., Jr. (2011). An aberrant thymus in CCR5-/- mice is coupled with an enhanced adaptive immune response in fungal infection. Journal of immunology (Baltimore, Md : 1950) 186, 5949-5955.

Hirai, H., Zhang, P., Dayaram, T., Hetherington, C.J., Mizuno, S., Imanishi, J., Akashi, K., and Tenen, D.G. (2006). C/EBPbeta is required for 'emergency' granulopoiesis. Nature immunology 7, 732-739.

Hsu, A.P., Sampaio, E.P., Khan, J., Calvo, K.R., Lemieux, J.E., Patel, S.Y., Frucht, D.M., Vinh, D.C., Auth, R.D., Freeman, A.F., et al. (2011). Mutations in GATA2 are associated with the autosomal dominant and sporadic monocytopenia and mycobacterial infection (MonoMAC) syndrome. Blood 118, 2653-2655.

Bustamante, J., Boisson-Dupuis, S., Abel, L., and Casanova, J.L. (2014). Mendelian susceptibility to mycobacterial disease: genetic, immunological, and clinical features of inborn errors of IFN-gamma immunity. Seminars in immunology 26, 454-470.

Watkins, C., Saleh, H., Song, E., Jaishankar, G.B., Chi, D.S., Misran, N., Peiris, E., Altrich, M.L., Barklow, T., and Krishnaswamy, G. (2012).

Concomitant gene mutations of MBL and CYBB in chronic granulomatous disease: implications for host defense. Inflammation & allergy drug targets 11, 222-226.

Hoekstra, W.J., Garvey, E.P., Moore, W.R., Rafferty, S.W., Yates, C.M., and Schotzinger, R.J. (2014). Design and optimization of highly-selective fungal CYP51 inhibitors. Bioorganic & medicinal chemistry letters 24, 3455-3458.

Lestner, J., and Hope, W.W. (2013). Itraconazole: an update on pharmacology and clinical use for treatment of invasive and allergic fungal infections. Expert opinion on drug metabolism & toxicology 9, 911-926.

Khan, H., Sabbah, D.A., Zafar, M., and Mubarak, M.S. (2018). Molecular modeling studies of coruscanone (A) core nucleus as potential antifungal agents. Life sciences 209, 332-340.

Morio, F., Loge, C., Besse, B., Hennequin, C., and Le Pape, P. (2010). Screening for amino acid substitutions in the Candida albicans Erg11 protein of azole-susceptible and azole-resistant clinical isolates: new substitutions and a review of the literature. Diagnostic microbiology and infectious disease 66, 373-384.

Fukuda, T. (2013). [Prophylaxis and treatment for invasive fungal infection: update]. [Rinsho ketsueki] The Japanese journal of clinical hematology 54, 1932-1939.

Fernandez-Silva, F., Capilla, J., Mayayo, E., Sutton, D., and Guarro, J.

(2014). Experimental murine acremoniosis: an emerging opportunistic human infection. Medical mycology 52, 29-35.

Salt, B.H., Niemela, J.E., Pandey, R., Hanson, E.P., Deering, R.P., Quinones, R., Jain, A., Orange, J.S., and Gelfand, E.W. (2008). IKBKG (nuclear factor-kappa B essential modulator) mutation can be associated with opportunistic infection without impairing Toll-like receptor function. The Journal of allergy and clinical immunology 121, 976-982.

Gladiator, A., Wangler, N., Trautwein-Weidner, K., and LeibundGut-Landmann, S. (2013). Cutting edge: IL-17-secreting innate lymphoid cells are essential for host defense against fungal infection. Journal of immunology (Baltimore, Md : 1950) 190, 521-525.

Trautwein-Weidner, K., Gladiator, A., Nur, S., Diethelm, P., and Leibund-Gut-Landmann, S. (2015). IL-17-mediated antifungal defense in the oral mucosa is independent of neutrophils. Mucosal immunology 8, 221-231.

Trautwein-Weidner, K., Gladiator, A., Nur, S., Diethelm, P., and LeibundGut-Landmann, S. (2015). IL-17-mediated antifungal defense in the oral mucosa is independent of neutrophils. Mucosal immunology 8, 221-231.

Cypowyj, S., Picard, C., Marodi, L., Casanova, J.L., and Puel, A. (2012). Immunity to infection in IL-17-deficient mice and humans. European journal of immunology 42, 2246-2254.

Eddens, T., Elsegeiny, W., Garcia-Hernadez, M.L., Castillo, P., Trevejo-Nunez, G., Serody, K., Campfield, B.T., Khader, S.A., Chen, K., Rangel-Moreno, J., et al. (2017). Pneumocystis-Driven Inducible Bronchus-Associated Lymphoid Tissue Formation Requires Th2 and Th17 Immunity. The Journal of dermatological treatment 18, 3078-3090.

Veverka, K.K., and Feldman, S.R. (2018). Chronic mucocutaneous candidiasis: what can we conclude about IL-17 antagonism? 29, 475-480.

Ketelut-Carneiro, N., Silva, G.K., Rocha, F.A., Milanezi, C.M., Cavalcanti-Neto, F.F., Zamboni, D.S., and Silva, J.S. (2015). IL-18 triggered by the Nlrp3 inflammasome induces host innate resistance in a pulmonary model of fungal infection. Immunological reviews 194, 4507-4517.

Man, S.M., Karki, R., and Kanneganti, T.D. (2017). Molecular mechanisms and functions of pyroptosis, inflammatory caspases and inflammasomes in infectious diseases. 277, 61-75.

Angkasekwinai, P., Sringkarin, N., Supasorn, O., Fungkrajai, M., Wang, Y.H., Chayakulkeeree, M., Ngamskulrungroj, P., Angkasekwinai, N., and Pattanapanyasat, K. (2014). Cryptococcus gattii infection dampens Th1 and Th17 responses by attenuating dendritic cell function and pulmonary chemokine expression in the immunocompetent hosts. Infection and immunity 82, 3880-3890.

Werner, J.L., Gessner, M.A., Lilly, L.M., Nelson, M.P., Metz, A.E., Horn, D.,

Dunaway, C.W., Deshane, J., Chaplin, D.D., Weaver, C.T., et al. (2011). Neutrophils produce interleukin 17A (IL-17A) in a dectin-1- and IL-23-dependent manner during invasive fungal infection. Infection and immunity 79, 3966-3977.

Sneideris, M. (1964). [ON PULMONARY CANDIDIASIS]. Sveikatos apsauga 9, 19-23.

Marra, E., Sousa, V.L., Gaziano, R., Pacello, M.L., Arseni, B., Aurisicchio, L., De Santis, R., and Salvatori, G. (2014). Efficacy of PTX3 and posaconazole combination in a rat model of invasive pulmonary aspergillosis. Antimicrobial agents and chemotherapy 58, 6284-6286.

Bozza, S., Campo, S., Arseni, B., Inforzato, A., Ragnar, L., Bottazzi, B., Mantovani, A., Moretti, S., Oikonomous, V., De Santis, R., et al. (2014). PTX3 binds MD-2 and promotes TRIF-dependent immune protection in aspergillosis. Journal of immunology (Baltimore, Md : 1950) 193, 2340 -2348.

Deepe, G.S., Jr., and Buesing, W.R. (2012). Deciphering the pathways of death of Histoplasma capsulatum-infected macrophages: implications for the immunopathogenesis of early infection. Journal of immunology (Baltimore, Md : 1950) 188, 334-344.

Pryhuber, G.S., Huyck, H.L., Bhagwat, S., O'Reilly, M.A., Finkelstein, J.N., Gigliotti, F., and Wright, T.W. (2008). Parenchymal cell TNF receptors

contribute to inflammatory cell recruitment and respiratory failure in Pneumocystis carinii-induced pneumonia. Journal of immunology(Baltimore, Md : 1950) 181, 1409-1419.
