# Supplementary material for: Xuebijing Injection Maintains GRP78 Expression to Prevent Candida albicans–Induced Epithelial Death in the Kidney
Source: Front Pharmacol. 2020 Jan 6;10:1416. doi: 10.3389/fphar.2019.01416 (PMC6956827; doi:10.3389/fphar.2019.01416)
Supplement: Supplementary file 5 [file Table_2.pdf]

Supplementary Table 2. The list of 36 compounds in XBJ that influence potential and proved targets of fungal infection.

Herb	Symbol	Synonym(s)	Family	PubChem ID	HMDB ID	CAS ID
1	3-n-butylphthalide	1(3H)-isobenzofuranone, 3-butyl-, 3-butylphthalide, butylphthalide, N-butylphthalide	chemical drug	--	--	
2	albiflorin		biologic drug			
3,5	alpha-tocopherol	2H-1-benzopyran-6-ol, 3,4-dihydro-2,5,7,8-tetramethyl-2-((4R,8R)-4,8,12-trimethyltridecyl)-, (2R)-, 59-02-9, (2R)-2,5,7,8-tetramethyl-2-[(4R,8R)-4,8,12-trimethyltridecyl]-3,4-dihydrochromen-6-ol, C29H50O2, D-alpha-tocopherol, D-α-tocopherol, α-tocopherol	chemical drug	14985	HMDB0001893	59-02-9
3,5	apigenin	4H-1-benzopyran-4-one, 5,7-dihydroxy-2-(4-hydroxyphenyl)-, 4H-1-benzopyran-4-one, 5,7-dihydroxy-2-(4-hydroxyphenyl)- (9CI), 5,7,4'-trihydroxyflavone, 5,7-dihydroxy-2-(4-hydroxyphenyl)chromen-4-one, 520-36-5, C15H10O5, flavone, 4',5,7-trihydroxy-	chemical - endogenous non-mammalian	5280443	HMDB0002124	520-36-5
5	astragalin	3,4',5,7-tetrahydroxyflavone-3-glucoside, 5,7-dihydroxy-2-(4-hydroxyphenyl)-3-[(2S,3R,4S,5S,6R)-3,4,5-trihydroxy-6-(hydroxymethyl)oxan-2-yl]oxychromen-4-one, 480-10-4, C21H20O11,	chemical - endogenous non-mammalian	5282102	--	480-10-4

		kaempferol 3-O-beta-D-glucopyranoside, kaempferol 3-O-β-D-glucopyranoside, kaempferol-3-O-glucoside, trifolin				
3	baicalin	7-D-glucuronic acid-5,6-dihydroxy-flavone, 21967-41-9, (2S,3S,4S,5R,6S)-6-(5,6-dihydroxy-4-oxo-2-phenylchromen-7-yl)oxy-3,4,5-trihydroxyoxane-2-carboxylic acid, baicalein 7-O-glucuronide, baicalein-7-D-glucuronide, beta-D-glucopyranosiduronic acid, 5,6-dihydroxy-4-oxo-2-phenyl-4H-1-benzopyran-7-yl, $C_{21}H_{18}O_{11}$, β-D-glucopyranosiduronic acid, 5,6-dihydroxy-4-oxo-2-phenyl-4H-1-benzopyran-7-yl	chemical - endogenous non-mammalian	64982	HMDB0041832	21967-41-9
3,5	caffeic acid	2-propenoic acid, 3-(3,4-dihydroxyphenyl)-, 2-propenoic acid, 3-(3,4-dihydroxyphenyl)- (9CI), 3,4-dihydroxybenzeneacrylic acid, 3,4-dihydroxycinnamic acid, 3-(3,4-dihydroxyphenyl)prop-2-enoic acid, 4-(2'-carboxyvinyl)-1,2-dihydroxybenzene, 4-(2-carboxyethenyl)-1,2-dihydroxybenzene, 331-39-5, $C_9H_8O_4$	chemical drug	2518	HMDB0001964	331-39-5

3	carnosol	2H-9,4a-(epoxymethano)phenanthren-12-one, 1,3,4,9,10,10a-hexahydro-5,6-dihydroxy-1,1-dimethyl-7-(1-methylethyl)-, (4aR-(4aalpha,9alpha,10abeta))-, 5957-80-2, 93241-27-1, C20H26O4	chemical - endogenous non-mammalian	442009	HMDB0002121	5957-80-2
5	carthamin yellow		biologic drug			
4	carvacrol	2-hydroxy-p-cymene, 2-methyl-5-propan-2-ylphenol, 2-p-cymenol, 5-isopropyl-2-methylphenol, 499-75-2, antioxine, C10H14O, isopropyl-o-cresol, isothymol, karvakrol, o-thymol, p-cymen-2-ol	chemical - endogenous non-mammalian	10364	HMDB0035770	499-75-2
3,5	chlorogenic acid	3-(3,4-dihydroxycinnamoyl)quinic acid, 3-caffeoylquinic acid, 327-97-9, (1S,3R,4R,5R)-3-[(E)-3-(3,4-dihydroxyphenyl)prop-2-enoyl]oxy-1,4,5-trihydroxycyclohexane-1-carboxylic acid, C16H18O9, chlorogenate, cyclohexanecarboxylic acid, 3-((3-(3,4-dihydroxyphenyl)-1-oxo-2-propenyl)oxy)-1,4,5-trihydroxy-, (1S-(1-alpha,3-beta,4-alpha,5-alpha))-, cyclohexanecarboxylic acid, 3-((3-(3,4-dihydroxyphenyl)-1-oxo-2-propenyl)oxy)-1,4,5-trihydroxy-, (1S-(1-α,3-β,4-α,5-α))-, trans-5-O-caffeoyl-D-qu	chemical drug	1794427	HMDB0003164	327-97-9

		inate, trans-5-O-caffeoyl-D-quinic acid				
3	cryptotanshinone	35825-57-1, (1R)-1,6,6-trimethyl-2,7,8,9-tetrahydro-1H-naphtho[1,2-g][1]benzofuran-10,11-dione, C19H20O3, phenanthro(1,2-b)furan-10,11-dione, 1,2,6,7,8,9-hexahydro-1,6,6-trimethyl-, (R)-	chemical drug	160254	HMDB0035220	35825-57-1
3	danshensu		biologic drug			
3	ferulic acid	2-Propenoic acid, 3-(4-hydroxy-3-methoxyphenyl)-, 4-hydroxy-3-methoxycinnamic acid, 537-98-4, 1135-24-6, 24276-84-4, (E)-3-(4-hydroxy-3-methoxyphenyl)prop-2-enoic acid, C10H10O4, sodium ferulate	chemical - endogenous non-mammalian	445858	HMDB0000954	24276-84-4
2	gallic acid	3,4,5-trihydroxybenzoic acid, 149-91-7, benzoic acid, 3,4,5-trihydroxy-, C7H6O5, gallate	chemical - endogenous non-mammalian	370	HMDB0005807	149-91-7
5	hydroxysafflor yellow a		biologic drug			
5	kaempferol	3,5,7-trihydroxy-2-(4-hydroxyphenyl)chromen-4-one, 520-18-3, C15H10O6	chemical toxicant	5280863	HMDB0005801	520-18-3
5	lauric acid	1-undecanecarboxylic acid, 12:0 fatty acid, 143-07-7, 629-25-4, 10124-65-9, C12 fatty acid, C12:0, C12:0 free fatty acid, C12H24O2, dodecanoate, dodecanoic acid, dodecylcarboxylate, laurate, n-dodecanoic	chemical - endogenous mammalian	3893	HMDB0000638	10124-65-9

		acid, sodium dodecanoate, sodium laurate				
1,5	linoleic acid	9,12-linoleic acid, 9,12-octadecadienoic acid (9Z,12Z)-, 9,12-octadecadienoic acid, (Z,Z)-, 9Z,12Z-linoleic acid, 60-33-3, 2197-37-7, (9Z,12Z)-octadeca-9,12-dienoic acid, alpha-linoleic acid, C18:2w6, C18H32O2, cis,cis-9,12-octadecadienoic acid, linoleate, linolelaidic acid, linolic acid, n-6 PUFA 18:2,6, octadecadienoic acid, α-linoleic acid	chemical - endogenous mammalian	5280450	HMDB0000673	60-33-3
3,5	luteolin	2-(3,4-dihydroxyphenyl)-5,7-dihydroxy-4-benzopyrone, 2-(3,4-dihydroxyphenyl)-5,7-dihydroxychromen-4-one, 3',4',5,7-tetrahydroxyflavone, 4H-1-benzopyran-4-one, 2-(3,4-dihydroxyphenyl)-5,7-dihydroxy- (9CI), 491-70-3, C15H10O6, cyanidenon 1470, digitoflavone, flacitran, luteolol	chemical - endogenous non-mammalian	5280445	HMDB0005800	491-70-3
3	luteoloside		biologic drug			
5	myricetin	3,5,7-trihydroxy-2-(3,4,5-trihydroxyphenyl)chromen-4-one, 529-44-2, C15H10O8	chemical - endogenous non-mammalian	5281672	HMDB0002755	529-44-2
5	oleanolic acid	3-beta-hydroxyolean-12-en-28-oic acid, 3-β-hydroxyolean-12-en	chemical - endogenous	10494	HMDB0002364	508-02-1

		-28-oic acid, 508-02-1, (4aS,6aR,6aS,6bR,8aR,10S,12aR,14bS)-10-hydroxy-2,2,6a,6b,9,9,12a-heptamethyl-1,3,4,5,6,6a,7,8,8a,10,11,12,13,14b-tetradecahydropicene-4a-carboxylic acid, astrantiagenin C, C30H48O3, caryophyllin, giganteumgenin C, olean-12-en-28-oic acid, 3-hydroxy-, (3beta)-, olean-12-en-28-oic acid, 3beta-hydroxy-, olean-12-en-28-oic acid, 3beta-hydroxy- (8CI), virgaureagenin B	non-mammalian			
2	oxypaeoniflorin		biologic drug			
1,2,3,4,5	palmitic acid	16:0 fatty acid, 57-10-3, 143-20-4, c16 fatty acid, C16:0, C16:0 fatty acid, C16:0 free fatty acid, C16H32O2, cetylic acid, hexadecanoate, hexadecanoic acid, hexadecylic acid, n-hexadecanoic acid, palmitate, palmitate acid, sodium palmitate	chemical - endogenous mammalian	985	HMDB0000220	143-20-4
2	peoniflorin	5b-((benzoyloxy)methyl)tetrahydro-5-hydroxy-2-methyl-2,5-methano-1H-3,4-dioxacyclobuta(cd) pentalen-1a(2H)-yl-beta-D-glucopyranoside, 5b-((benzoyloxy)methyl)tetrahydro-5-hydroxy-2-methyl-2,5-methano-1H-3,4-dioxacyclobuta(cd) pentalen-1a(2H)-yl-β-D-	chemical drug	5458396	--	23180-57-6

		glucopyranoside, 5b-((benzoyloxy)methyl)tetrahydro-5-hydroxy-2-methyl-2,5-methano-lH-3,4-dioxacyclobuta(cd)pentalen-1a(2H)-yl-beta-D-glucopyranoside, 5b-((benzoyloxy)methyl)tetrahydro-5-hydroxy-2-methyl-2,5-methano-lH-3,4-dioxacyclobuta(cd)pentalen-1a(2H)-yl-β-D-glucopyranoside, 23180-57-6, beta-D-glucopyranoside, 5b-((benzoyloxy)methyl)tetrahydro-5-hydroxy-2-methyl-2,5-methano-1H-3,4-dioxacyclobuta(cd)pentalen-1a(2H)-yl, (1aR-(1a-alpha,2-beta,3a-alpha,5-alpha,5a-alpha,5b-alpha))-, C23H28O11, paeonia moutan, paeoniflorin, paeony root, β-D-glucopyranoside, 5b-((benzoyloxy)methyl)tetrahydro-5-hydroxy-2-methyl-2,5-methano-1H-3,4-dioxacyclobuta(cd)pentalen-1a(2H)-yl, (1aR-(1a-α,2-β,3a-α,5-α,5a-α,5b-α))-				
3	protocatechualdehyde		biologic drug			
5	quercetin	2-(3,4-dihydroxyphenyl)-3,5,7-trihydroxychromen-4-one, 3,3',4,5,7-pentahydroxyflavone, 3,5,7,3',4'-pentahydroxyflavone, 4H-1-benzopyran-4-one, 2-(3,4-dihydroxyphenyl)	chemical drug	5280343	HMDB0005794	117-39-5

		-3,5,7-trihydroxy-, 117-39-5, C15H10O7, cyanidanol, flavin meletin, meletin, quercetin dihydrate, quercitin				
3	rosmarinic acid	3-(3,4-dihydroxyphenyl)-2-[(E)-3-(3,4-dihydroxyphenyl)prop-2-enoyl]oxypropanoic acid, 537-15-5, benzenepropanoic acid, alpha-((3-(3,4-dihydroxyphenyl)-1-oxo-2-propenyl)oxy)-3,4-dihydroxy-, benzenepropanoic acid, α-((3-(3,4-dihydroxyphenyl)-1-oxo-2-propenyl)oxy)-3,4-dihydroxy-, C18H16O8, rosmarinate	chemical - endogenous non-mammalian	5315615	--	537-15-5
3,5	rutin	2-(3,4-dihydroxyphenyl)-5,7-dihydroxy-3-[(2S,3R,4S,5S,6R)-3,4,5-trihydroxy-6-[[(2R,3R,4R,5R,6S)-3,4,5-trihydroxy-6-methyloxan-2-yl]oxymethyl]oxan-2-yl]oxychromen-4-one, 3,3',4',5,5',7-hexahydroxyflavone (6-O-alpha-L-rhamnosyl-beta-D-glucoside), 3,3',4',5,5',7-hexahydroxyflavone (6-O-α-L-rhamnosyl-β-D-glucoside), 3,3',4',5,7-pentahydroxyflavone-3-rutinoside, 4H-1-benzopyran-4-one, 3-((6-O-(6-deoxy-alpha-L-mannopyranosyl)-beta-D-glucopyranosyl)oxy)-2-(3,4-dihydroxyphenyl)-5,7-dihydroxy-,	chemical toxicant	5280805	HMDB0003249	153-18-4

		4H-1-benzopyran-4-one, 3-((6-O-(6-deoxy-α-L-mannopyranosyl)-β-D-glucopyranosyl)oxy)-2-(3,4-dihydroxyphenyl)-5,7-dihydroxy-, 153-18-4, C27H30O16, glucopyranoside, quercetin-3 6-O-alpha-L-rhamnopyranosyl-, beta-D, glucopyranoside, quercetin-3 6-O-α-L-rhamnopyranosyl-, β-D, phytomelin, quercetin-3-rutinoside, rutoside				
5	safflor yellow A		biologic drug			
3	salvianolic acid A		biologic drug			
3	salvianolic acid B		biologic drug			
2	sitosterol	3beta-sitosterol, 5779-62-4, (3S,8S,9S,10R,13R,14S,17R)-17-[(2R,5R)-5-ethyl-6-methylheptan-2-yl]-10,13-dimethyl-2,3,4,7,8,9,11,12,14,15,16,17-dodecahydro-1H-cyclopenta[a]phenanthren-3-ol, C29H50O	chemical - endogenous non-mammalian	222284	--	5779-62-4
2	syringin	118-34-3, (2R,3S,4S,5R,6S)-2-(hydroxymethyl)-6-[4-[(E)-3-hydroxyprop-1-enyl]-2,6-dimethoxyphenoxy]oxane-3,4,5-triol, C17H24O9, eleutheroside B, lilacin, methoxyconiferine, syringenin	chemical - endogenous non-mammalian	5316860	--	118-34-3
3	ursolic acid	77-52-1, (1S,2R,4aS,6aR,6aS,6bR,8aR,10S,12aR,14bS)-10-hydroxy-1,2,6a,6b,9,	chemical drug	64945	HMDB0002395	77-52-1

		9,12a-heptamethyl-2,3,4,5,6,6a,7,8,8a,10,11,12,13,14b-tetradecahydro-1H-picene-4a-carboxylic acid, C30H48O3				

Note: *1. Ligusticum chuanxiong rhizomes (Chuanxiong); 2. Paeonia lactiflora roots (Chishao); 3. Salvia miltiorrhiza roots (Danshen); 4. Angelica sinensis roots (Danggui); 5. Carthamus tinctorius flowers (Honghua).*
